# Global Burden of Early‐Onset Esophageal Cancer From 1990 to 2021: A Systematic Analysis of the Global Burden of Disease Study 2021

**DOI:** 10.1111/1759-7714.70082

**Published:** 2025-05-21

**Authors:** Guotian Pei, Yuqing Huang, Yingshun Yang, Shuai Wang, Shushi Meng, Jun Liu, Hongjing Jiang

**Affiliations:** ^1^ Department of Minimally Invasive Esophageal Surgery Tianjin Medical University Cancer Institute and Hospital, National Clinical Research Center for Cancer, Key Laboratory of Cancer Prevention and Therapy, Tianjin's Clinical Research Center for Cancer Tianjin China; ^2^ Department of Thoracic Surgery Beijing Haidian Hospital (Haidian Section of Peking University Third Hospital) Beijing China; ^3^ Department of Thoracic Surgery Peking University People's Hospital Beijing China

**Keywords:** esophageal cancer, frontier analysis, global burden of disease study, joinpoint regression, socio‐demographic index

## Abstract

**Objective:**

To estimate the global burden of early‐onset esophageal cancer (EOEC) and its associated risk factors from 1990 to 2021.

**Methods:**

We utilized data from the Global Burden of Disease study 2021 to assess EOEC incidence, mortality, and disability‐adjusted life years (DALYs) across 204 countries. Statistical modeling techniques, including age‐period‐cohort analysis and joinpoint regression, were employed to assess trends.

**Results:**

In 2021, the global age‐standardized incidence rate (ASIR), age‐standardized mortality rate (ASMR), and age‐standardized DALYs rate (ASDR) for EOEC were 1.24 (95% CI: 1.19–1.40), 0.95 (95% CI: 0.85–1.08), and 45.07 (95% CI: 40.03–50.89) per 100 000 population, respectively. The highest burden was observed in countries with high‐middle SDI, while high‐SDI countries had the lowest rates. Regionally, Southern Sub‐Saharan Africa had the highest ASIR and ASMR, whereas Andean Latin America reported the lowest. Men consistently exhibited higher incidence and mortality rates than women, with rates approximately three times greater. Between 1990 and 2021, significant global reductions in ASIR, ASMR, and ASDR were observed, particularly between 2003 and 2007, driven primarily by improvements in high‐SDI countries. Smoking and alcohol consumption emerged as predominant risk factors, contributing substantially to DALYs in high‐ and middle‐SDI countries, while low vegetable intake was a key risk factor in low‐SDI countries.

**Conclusions:**

While EOEC incidence and mortality have declined globally, persistent disparities in low‐SDI countries demand urgent attention. Prioritizing risk factor interventions will be essential in high‐risk populations. Coordinated global efforts, aligned with international health goals, are crucial to closing the gap and addressing regional needs.

## Graphical abstract

This study reveals a significant global decline in early‐onset esophageal cancer (EOEC) incidence, mortality, and DALYs from 1990 to 2021, with marked disparities across socio‐demographic index (SDI) categories. Southern Sub‐Saharan Africa exhibits the highest burden, while high‐SDI countries show the lowest rates. Smoking, alcohol use, and low vegetable intake are key risk factors, with regional variations influencing EOEC outcomes.

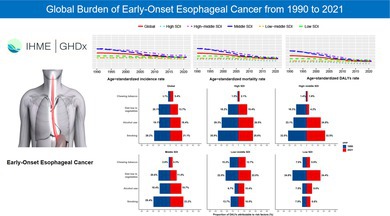

## 1 Introduction

Esophageal cancer is one of the most prevalent and lethal malignancies globally, ranking as the 11th most commonly diagnosed cancer and the 7th leading cause of cancer death worldwide, with an estimated 511 000 new cases and 445 000 deaths reported in 2022 [1]. The incidence, mortality, and prognosis of esophageal cancer show significant variation across age groups, sexes, and geographical regions [2]. While the disease is predominantly diagnosed in older adults, there has been a concerning rise in early‐onset esophageal cancer (EOEC) [3, 4, 5], defined as occurring in individuals under 50 years of age [6, 7]. This trend has been observed in several regions, including both high and low socio‐economic settings, and has been linked to a complex interplay of risk factors, including smoking, alcohol consumption, obesity, and gastroesophageal reflux disease [8, 9]. Despite growing awareness of EOEC, the global burden remains inadequately addressed, with a notable gap in studies that focus on younger populations [10].

Although there is substantial literature on esophageal cancer, several key gaps remain in our understanding of EOEC. First, the risk factors associated with esophageal cancer are well documented for older populations, but there is limited research on how these factors impact younger cohorts differently [11]. Second, while regional disparities in the burden of EOEC have been reported, the influence of socio‐economic factors on the disease burden has not been adequately explored [12]. Socio‐economic development plays a crucial role in healthcare access, lifestyle behaviors, and cancer prevention strategies. For example, regions with lower socio‐economic status often lack the infrastructure and resources for early screening, contributing to a higher burden of EOEC. Additionally, socio‐economic factors, such as income and education levels, have been shown to influence lifestyle behaviors that contribute to the development of EOEC. Understanding these socio‐economic influences is essential to designing targeted interventions and reducing disparities in EOEC outcomes globally.

To address these knowledge gaps, this study provides a comprehensive evaluation of the global, regional, and national burden of EOEC from 1990 to 2021, utilizing data from the Global Burden of Disease (GBD) 2021 database. By assessing the incidence, mortality, and disability‐adjusted life years (DALYs) associated with EOEC, stratified by age, sex, and socio‐demographic index (SDI), this study aims to elucidate global patterns and socio‐economic influences on EOEC outcomes. Using advanced analytical methods such as joinpoint regression, age‐period‐cohort modeling, and frontier analysis, we seek to deepen our understanding of EOEC trends and inform policy decisions aimed at improving cancer prevention, early screening, and control strategies among younger individuals. This study is timely and essential, especially in light of the projected increase in EOEC burden due to ongoing socio‐economic disparities and insufficient healthcare resources in certain regions.

## 2 Methods

### 2.1 Overview

This study is part of GBD 2021, which assessed the burden of 371 diseases, injuries, and 88 risk factors across 204 countries and territories from 1990 to 2021 [13]. Detailed descriptions of the methodologies for assessing disease burden and evaluating risk factors are provided elsewhere [14, 15, 16]. The study adhered to the Guidelines for Accurate and Transparent Health Estimates Reporting (GATHER) [17]. Ethical approval was not required since the study utilized publicly available data and did not involve personal information.

### 2.2 Data Source

Data for the GBD 2021 were derived from multiple validated sources, including vital registration systems (23 111 site‐years), sample registrations (825 site‐years), verbal autopsy records (420 site‐years), and cancer registries (5206 site‐years) [15]. In accordance with the International Classification of Diseases, 10th edition (ICD‐10), all cancers coded C15.0–C15.9, D00.1, and D13.0 were classified as esophageal cancers [14]. Four sequelae with associated disability weights were used: diagnosis, controlled, metastatic, and terminal phases (Table S1). Disability weights ranged from 0 (representing full health) to 1 (representing death). Given that GBD 2021 includes esophageal cancer cases only for individuals aged 20 and above, and EOEC is typically defined as occurring in individuals under 50, our study focuses specifically on individuals aged 20–49 (defined as early‐onset esophageal cancer, EOEC). We obtained data on incidence, mortality, and DALYs for EOEC from the Global Health Data Exchange (https://ghdx.healthdata.org/gbd‐results‐tool), stratified by age, sex, and region, along with DALYs attributable to various risk factors, including their 95% uncertainty intervals (UI).

### 2.3 Estimation of Mortality, Incidence, and DALYs

GBD 2021 employed a comprehensive and systematic approach to estimate mortality, incidence, and DALYs associated with esophageal cancer. The GBD database first consolidates mortality data from various sources, including vital registration systems, verbal autopsy, and cancer registry data, which are standardized to ensure comparability across sources. Cause‐specific models, such as the Cause of Death Ensemble model (CODEm), are used to estimate esophageal cancer deaths by age, sex, year, and geographic location. Incidence data are derived from cancer registries and other relevant sources, and modeled alongside death data to calculate DALYs. Detailed methodologies used in GBD 2021 are provided in the Supporting Information.

### 2.4 Risk Factor Estimation and SDI

We calculated and reported the proportion of esophageal cancer DALYs attributable to smoking, alcohol consumption, low vegetable intake, and chewing tobacco. The definitions of these risk factors and their relative risk for esophageal cancer have been detailed previously [16]. Additionally, the SDI was used to assess the relationship between a country's socioeconomic development and the age‐standardized incidence and mortality rates of EOEC. Details about SDI are provided in the Supporting Information.

### 2.5 Statistics Analysis

Age‐standardized rates and corresponding 95% confidence intervals (CI) were calculated using standardization based on the world standard population reported in the GBD Study 2021 [18]. All rates are presented per 100 000 population. Details of the standardization methods are provided in the Supporting Information. To comprehensively assess the burden of EOEC, we analyzed trends in incidence, mortality, and DALYs at global, regional, and national levels from 1990 to 2021. Annual percent change (APC) and average annual percent change (AAPC), along with their corresponding 95% CI, were calculated using joinpoint regression analysis (see the Supporting Information) [19, 20]. Conversely, an APC or AAPC estimate with a 95% CI upper bound below zero suggests a downward trend. If the 95% CI for APC or AAPC includes zero, it indicates a stable trend.

The association between EOEC burden and SDI across 21 regions and 204 countries and territories was assessed using smoothing spline models. The computation accounting for SDI and disease rates in each location produced the predicted values. The Locally Weighted Scatterplot Smoothing method, which automatically calculates the degree, number, and placement of nodes based on data and span parameters, was used to fit smooth splines. *R* indices and *p* values for the age‐standardized rate and SDI association were calculated using Spearman correlation analysis. Frontier analysis was employed to compare the performance of countries and territories with the best‐performing ones, establishing benchmarks for EOEC burden [21]. This method identifies top‐performing countries and territories, setting standards and objectives for others. The “effective difference” (the difference between current and potential EOEC burden) for each country and territory was computed, accounting for SDI.

Lastly, the age‐period‐cohort model within the maximum likelihood framework, along with the Bayesian age‐period‐cohort (BAPC) model using nested Laplace approximations, was employed to forecast the future disease burden from 2022 to 2050 [22, 23]. All statistical analyses were performed using R software (version 4.2.2). Statistical significance was set at *p* < 0.05.

## 3 Results

### 3.1 Global Distribution of EOEC in 2021

**FIGURE 1:**
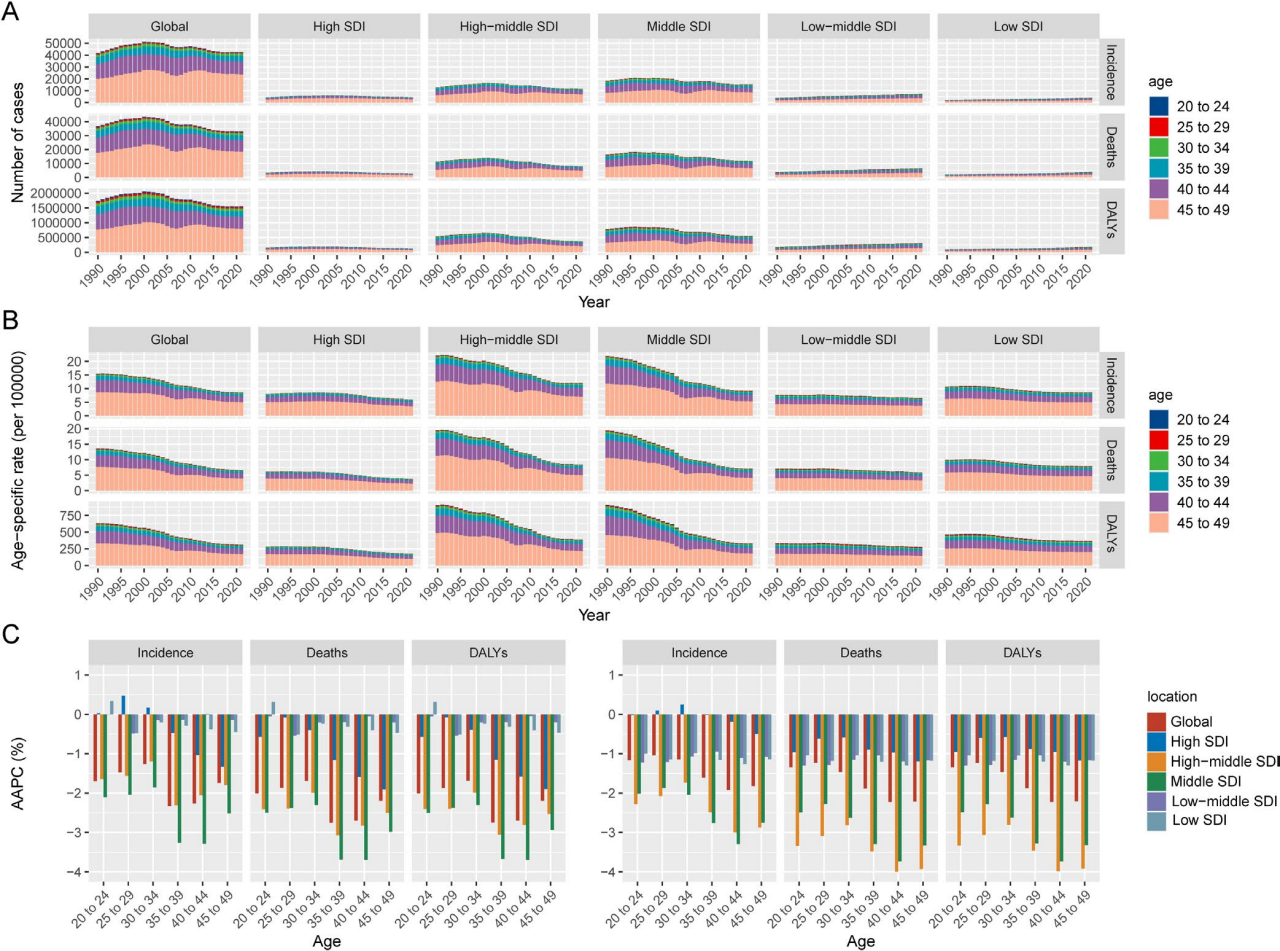
Temporal trends in the age‐specific burden of early‐onset esophageal cancer globally and across SDI categories from 1990 to 2021. (A) The number of cases (incidence, deaths, and DALYs) by year, age group (20–49 years), and SDI quintile. (B) Age‐specific rates for incidence, deaths, and DALYs across the same time period, stratified by SDI region and age group. (C) AAPC for incidence, deaths, and DALYs by age group, stratified by SDI quintile, from 1990 to 2021. The left panels represent data for men, while the right panels represent data for women. AAPC = average annual percentage change, DALYs = disability‐adjusted life years, SDI = socio‐demographic index.

In 2021, the global age‐standardized incidence rate (ASIR), mortality rate (ASMR), and DALYs rate (ASDR) per 100 000 population for EOEC were 1.24 (95% CI: 1.19–1.40), 0.95 (95% CI: 0.85–1.08), and 45.07 (95% CI: 40.03–50.89), respectively for both sexes (Table S2). By SDI category, countries with high‐middle SDI had the highest ASIR (1.73 per 100 000 population, 95% CI: 1.38–2.17), ASMR (1.19, 95% CI: 0.96–1.50), and ASDR (56.10, 95% CI: 45.03–70.42), while high‐SDI countries had the lowest ASIR (0.85, 95% CI: 0.81–0.90), ASMR (0.54, 95% CI: 0.51–0.57), and ASDR (25.35, 95% CI: 24.06–26.72) (Table S2). Regionally, Southern Sub‐Saharan Africa exhibited the highest burden of EOEC, with the highest ASIR (2.72, 95% CI: 2.31–3.18), ASMR (2.46, 95% CI: 2.09–2.88), and ASDR (115.48, 95% CI: 97.92–135.05). The lowest burden was observed in Andean Latin America, with an ASIR of 0.23 (95% CI: 0.17–0.30), ASMR of 0.20 (95% CI: 0.15–0.26), and ASDR of 9.55 (95% CI: 7.16–12.67) (Table S2). Nationally, Malawi recorded the highest ASIR (6.27, 95% CI: 4.19–9.43), ASMR (5.76, 95% CI: 3.86–8.45), and ASDR (271.01, 95% CI: 180.94–399.36). The top 10 countries were predominantly from Eastern Sub‐Saharan Africa, while Tunisia recorded the lowest rates, with the lowest burden countries mainly from North Africa and the Middle East (Figure S1, Table S4). Globally, the ASIR, ASMR, and ASDR were higher for men (ASIR: 1.88, 95% CI: 1.26–1.68; ASMR: 1.45, 95% CI: 1.26–1.68; ASDR: 68.24, 95% CI: 59.12–78.56 per 100 000 population) than for women (ASIR: 0.58, 95% CI: 0.49–0.70; ASMR: 0.45, 95% CI: 0.38–0.54; ASDR: 21.58, 95% CI: 18.26–26.29 per 100 000 population). The rates for men were approximately three times higher than those for women (Table S2). Age‐specific incidence, mortality, and DALY rates all increased with age (Figure 1, Table S5).

### 3.2 Temporal Trend of EOEC From 1990 to 2021

**FIGURE 2:**
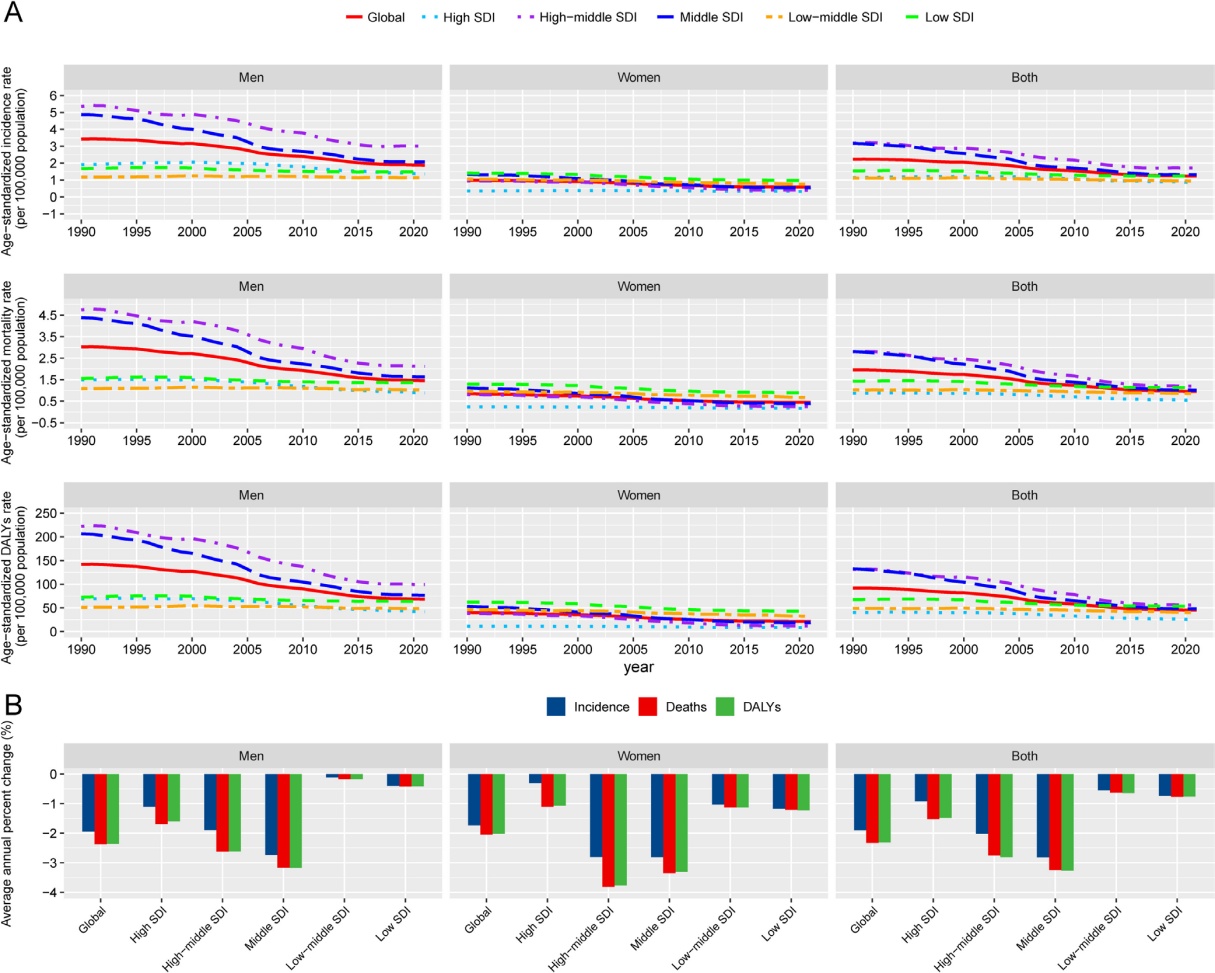
Temporal trends of the age‐standardized incidence rate, age‐standardized mortality rate, and age‐standardized DALYs rate for the burden of early‐onset esophageal cancer, globally and by SDI from 1990 to 2021. The SDI categories include five levels: high, high‐middle, middle, low‐middle, and low. The figure illustrates the trends across these categories, highlighting global patterns as well as differences among SDI countries (A). Additionally, the average annual percent change (AAPC) for each indicator, both globally and by SDI category, is presented for the same period (B). DALYs = disability‐adjusted life years, SDI = socio‐demographic index.

From 1990 to 2021, the number of cases, crude rate, and age‐standardized rates of incidence, mortality, and DALYs showed a global downward trend for both sexes, indicating a reduced disease burden. This decline was more pronounced in men than in women (Figure S2, Table S2). Globally, the ASIR decreased from 2.23 (95% UI: 2.51–1.98) in 1990 to 1.24 (95% UI: 1.40–1.09) in 2021(Figure 2, Table S2). Joinpoint regression analysis revealed a global downward trend in ASIR (AAPC = −1.90%, 95% CI: −2.05 to −1.75%, *p* < 0.001), with the most significant decline occurring between 2003 and 2007 (APC = −4.17%, 95% CI: −4.69 to −3.65%, *p* < 0.001). Similarly, both the ASMR and ASDR showed consistent declines from 1990 to 2021, with an AAPC of −2.33% (95% CI: −2.51 to −2.14, *p* < 0.001) for ASMR and − 2.31% (95% CI: −2.49 to −2.14, *p* < 0.001) for ASDR. Additionally, the sharpest decline occurred between 2003 and 2007, with an APC of −5.03% (95% CI: −5.64% to −4.42%, *p* < 0.001) for ASMR and − 4.87% (95% CI: −5.46% to −4.28%, *p* < 0.001) for ASDR (Figure S3, Table S3). A consistent downward trend in age‐specific incidence, mortality, and DALY rates for EOEC was observed across global and SDI subgroups when analyzed by age. For men, the sharpest decline occurred in the 35–39 age group, where the incidence rate fell from 2.54 to 1.24 per 100 000, with an AAPC of −2.33%, indicating a significant reduction. For women, the steepest decline was in the 40–44 age group, where the incidence rate dropped from 1.82 to 1.00 per 100 000, with an AAPC of −1.92% (Figure 2, Table S5).

**FIGURE 3:**
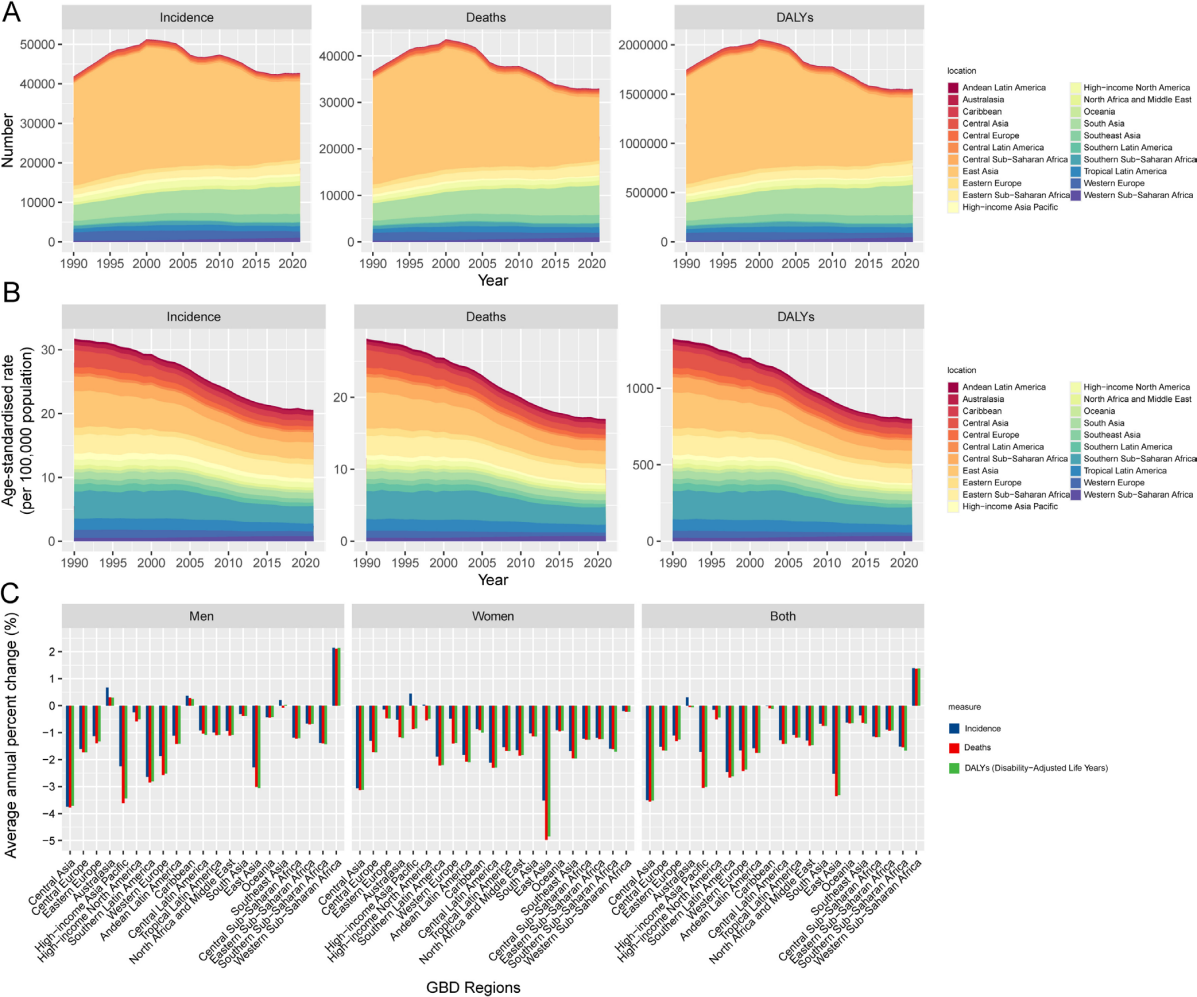
Temporal trends and average annual percent change (AAPC) in the burden of early‐onset esophageal cancer across different regions from 1990 to 2021. (A) The number of cases (incidence, deaths, and DALYs) due to early‐onset esophageal cancer from 1990 to 2021 across different regions. (B) The age‐standardized incidence rate, mortality rate, and DALYs per 100 000 population for early‐onset esophageal cancer, also stratified by regions. (C) The AAPC in incidence, mortality, and DALYs for early‐onset esophageal cancer from 1990 to 2021, stratified by region and gender (men, women, and both combined). DALYs = disability‐adjusted life years.

Regionally, the greatest reduction in incidence, mortality, and DALYs for EOEC from 1990 to 2021 was in Central Asia, with incidence decreasing from 2.69 to 0.89 per 100 000 (AAPC −3.51%), mortality from 2.97 to 0.89 (AAPC −3.77%), and DALYs from 139.33 to 42.52 (AAPC −3.71%) (Figure 3). The corresponding indicators and AAPC for other regions between 1990 and 2019 are provided in Table S3. In Central Asia, men showed the largest decline in ASIR, ASMR, and ASDR, while in East Asia, these rates dropped the most for women. Across GBD regions, the disease burden of EOEC varies significantly. To identify regions that exhibit similar patterns in disease burden, we conducted a hierarchical clustering analysis. The results are shown in Figure S4.

Nationally, Kazakhstan experienced the largest decline in ASIR, dropping from 3.64 per 100 000 in 1990 to 0.81 per 100 000 in 2021, with an AAPC of −4.96% (95% CI: −5.12 to −4.80). The Republic of Korea saw the sharpest decline in both ASMR and ASDR, with ASMR falling from 0.89 to 0.18, and ASDR from 40.93 to 8.40. The AAPCs were − 5.09% (95% CI: −5.55 to −4.63) for ASMR and − 5.03% (95% CI: −5.48 to −4.58) for ASDR (Figures S5 and S6). The relevant indicators and AAPC values for other countries are provided in Table S4.

**FIGURE 4:**
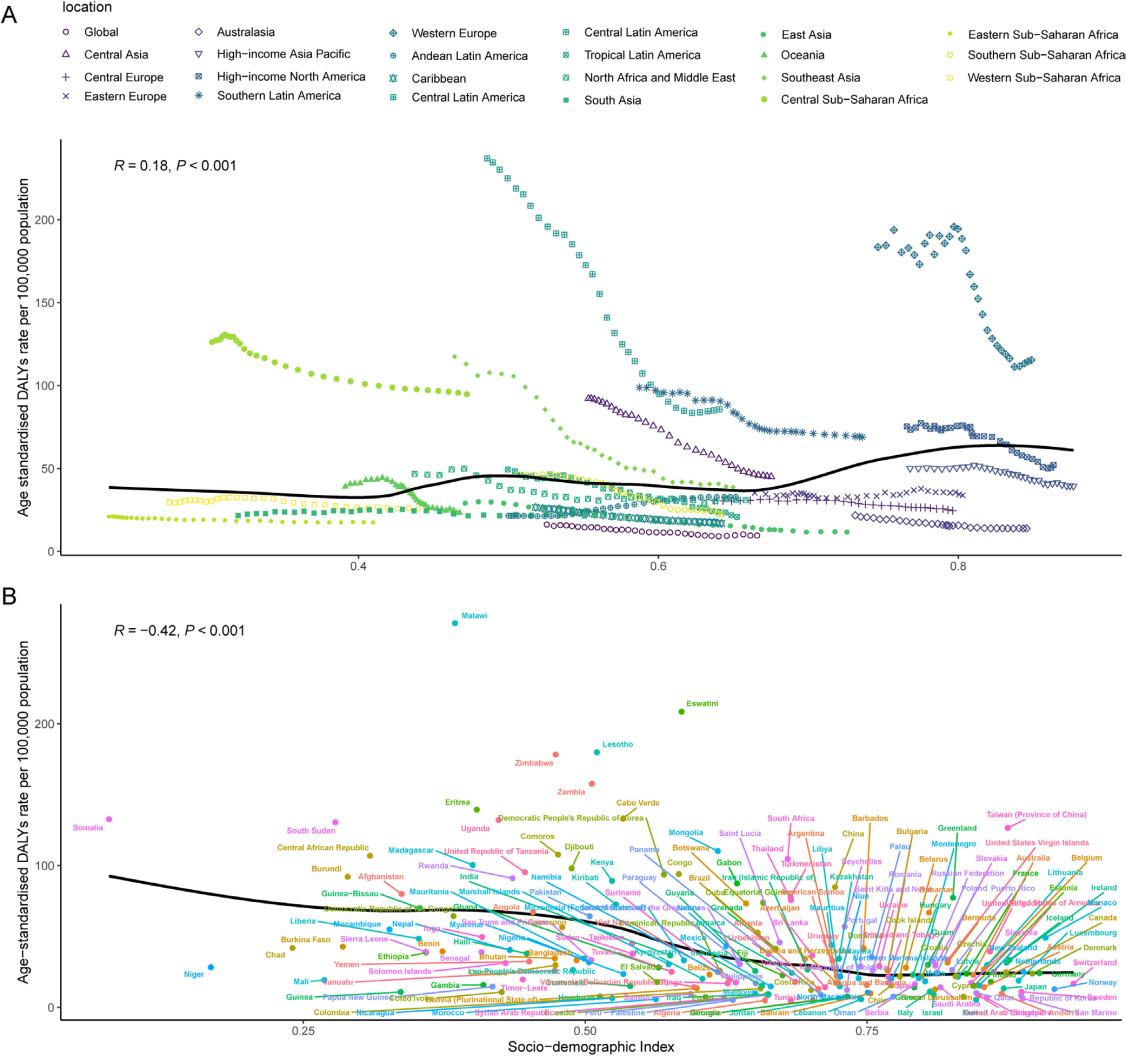
Age‐standardized DALYs rates for early‐onset esophageal cancer across 21 GBD regions (A) and 204 countries and territories (B) by SDI, 1990–2021. (A) Expected values, based on the SDI and disease rates across all locations, are represented by the solid line. These values are calculated to reflect the expected disease burden when accounting for SDI and disease rates globally. For each region, 32 points are plotted, corresponding to the observed age‐standardized disability‐adjusted life year (DALY) rates for each year from 1990 to 2021. Points located above the solid line indicate a higher‐than‐expected burden, while points below the line represent a lower‐than‐expected burden. (B) Countries and territories positioned above the solid line indicate a higher‐than‐expected burden, while those below the line reflect a lower‐than‐expected burden. SDI = socio‐demographic index.

Regional and national ASDR in relation to SDI, compared to expected levels based on SDI, are presented in Figure 4. Some high‐income regions, like Central Europe, Eastern Europe, and Tropical and Southern Latin America, closely followed the expected trends during the study period. In contrast, many middle‐SDI countries showed considerable variation. For example, some Central Asian countries had ASDR significantly lower than expected, with minimal change throughout the study period. Conversely, some Tropical Latin American countries, despite higher‐than‐expected ASDR, exhibited fluctuating or decreasing trends. Nationally, in 2021, an inverse relationship was observed between ASDR and SDI, where ASDR decreased as SDI increased in some countries. However, this inverse association was not present in all countries. The relationships between ASIR and ASMR with SDI showed similar patterns (Figure S7).

### 3.3 Frontier Analysis

A comprehensive frontier analysis based on SDI and ASR of EOEC from 1990 to 2021 across 204 countries and territories revealed distinct trends. For ASMR, as SDI increased from 0 to 1.0, an overall downward trend in mortality rates was observed, marked by a shift from darker to lighter shades over the years, indicating a general reduction in mortality. Similarly, as SDI increased, ASDR followed the same pattern, revealing that with social development, the burden of EOEC has significantly decreased. The 2021 frontier analysis visually highlighted differences among countries and territories. Regardless of ASDR or ASMR, countries like South Sudan, Zambia, and the Central African Republic had significantly higher rates, positioning them far from the frontier. In contrast, countries like Côte d'Ivoire, Guinea, and Gambia were closer to the frontier, indicating better outcomes relative to their level of development (Figure S8, Table S6).

### 3.4 Risk Factors

For level 2 risks, the global disease burden, measured in DALYs, was highest for tobacco in 2021, followed by dietary risks and alcohol use. In 2021, 0.4 million (95% UI: 0.32–0.5) DALYs were attributed to smoking, 0.21 million (0.43–0.45) to dietary risks, and 0.29 million (0.21–0.38) to alcohol use (Figure S9, Table S7). The ASDR attributable to the three leading global level 2 risk factors in 2021 varied by geography (Figure S10).

**FIGURE 5:**
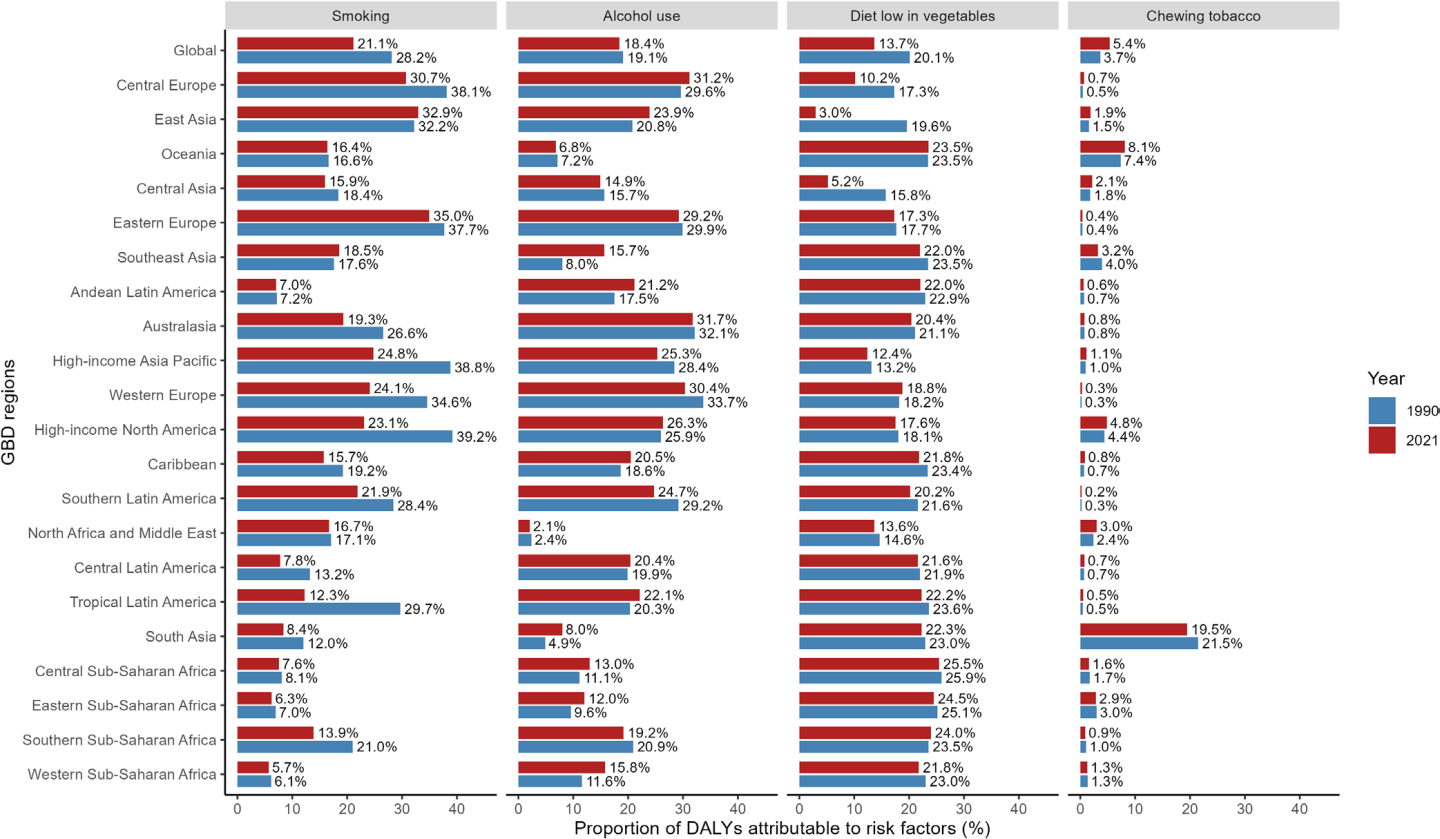
Proportion of early‐onset esophageal cancer DALYs attributable to four risk factors in 1990 and 2021 across 21 GBD regions. DALYs = disability‐adjusted life years, GBD = Global Burden of Diseases, Injuries, and Risk Factors Study.

The ASDR attributable to the top three global level 2 risk factors in 2021 varied by region. Globally, a substantial portion of DALYs is attributable to four major risk factors spanning three level 2 risks: tobacco (smoking and chewing), alcohol use, and dietary risks (low vegetable intake). In 2021, for DALYs, 21.1% was attributed to smoking, 18.4% to alcohol use, 13.7% to low vegetable intake, and 5.4% to chewing tobacco (Table S8). The impact of these risk factors varies across regions (Figure 5). For instance, smoking had the highest impact on DALYs in Eastern Europe and East Asia, while its impact was lowest in Western Sub‐Saharan Africa. Similarly, alcohol consumption had the highest impact in Australasia and Central Europe, and the lowest in North Africa and the Middle East, where alcohol consumption is relatively low. Regarding SDI, smoking and alcohol use were the top attributable risk factors in countries with high, high‐middle, and middle SDI. In low‐middle and low SDI countries, more DALYs were attributed to low vegetable intake (Table S9). From a gender perspective, in 2021, smoking was the top risk factor for DALYs in men, with its impact increasing with age. For women, low vegetable intake was the leading risk factor, with minimal variation by age (Figure S11, Table S10).

From 1990 to 2021, global DALYs due to level 2 risks, including tobacco (AAPC: −3.00%), dietary risks (−3.56%), and alcohol use (−2.44%), significantly declined, reflecting improvements in public health strategies (Table S7). For level 3 risks, the proportion of DALYs attributable to smoking, alcohol use, and low vegetable intake—excluding chewing tobacco—has declined globally (Figure S12). Across genders, the proportion of DALYs attributed to smoking and low vegetable intake has decreased, alcohol use has remained stable, while chewing tobacco has shown an upward trend. The proportion of DALYs attributable to alcohol use decreased only in high‐SDI countries (from 29.34% to 26.51%) but increased in countries with low SDI level. The proportion attributable to smoking remained stable in high‐middle SDI countries but declined in other SDI countries. The proportion of DALYs attributable to low vegetable intake has decreased across all SDI countries. The proportion attributable to chewing tobacco decreased in low‐middle and low SDI countries but increased in high‐SDI countries (Figure S13, Table S9).

### 3.5 Predictive Analysis on EOEC Burden to 2050

By 2050, an estimated 84 909 (95% UI: 0–286 787) cases and 63 692 (95% UI: 0–228 833) deaths from EOEC are expected globally, with a projected ASIR of 2 per 100 000 people (0–6.88 cases) and a projected ASMR of 1.5 per 100 000 people (0–5.5 cases). In 2050, Southern Sub‐Saharan Africa is projected to have the highest ASIR (1644 [0–179 483] per 100 000) and the highest ASMR (983 [0–100 321] per 100 000) for EOEC. Detailed values for case numbers and ASR of incidence and mortality are provided in Figure S14 and Table S11.

## 4 Discussion

This study comprehensively analyzed the global disease burden of EOEC from 1990 to 2021 using data from the GBD 2021. By concentrating on individuals aged 20–49, we aimed to provide specific insights into the dynamics and determinants of EOEC in younger populations. The analysis yielded several key findings: firstly, a significant overall decline in the incidence, mortality, and DALYs associated with EOEC was observed globally, with the most substantial reductions occurring between 2003 and 2007. Secondly, considerable geographic variation was noted in the burden of EOEC, with Southern Sub‐Saharan Africa exhibiting the highest incidence, mortality, and DALY rates, whereas Andean Latin America demonstrated the lowest. Thirdly, sex‐based disparities were evident, with men demonstrating consistently higher rates across all measures compared to women. The association between EOEC burden and the SDI highlighted the impact of socio‐economic development on disease outcomes. This research contributes novel insights by examining the intersection between EOEC burden and socio‐economic disparities, which has been less examined in prior studies. Furthermore, the temporal analysis of global, regional, and national trends offers a comprehensive understanding of how public health initiatives, socio‐economic development, and modifications in risk factors have shaped EOEC outcomes over the past three decades. Moreover, this study applied frontier and predictive analyses, providing essential perspectives on the future trajectory of EOEC burden, which could inform global cancer control strategies.

The observed decline in ASIR, ASMR, and DALYs for EOEC from 1990 to 2021 aligns with previous studies indicating reductions in esophageal cancer incidence in high‐income regions, attributable to advances in cancer screening, early detection, and the mitigation of high‐risk behaviors such as smoking and alcohol consumption [2, 6]. Enzinger and Mayer emphasized that early detection programs and improved treatments have contributed to reduced mortality, particularly in high‐SDI countries [6]. Our findings show that high‐SDI countries have indeed benefited from these improvements, exhibiting the lowest ASIR and ASMR in 2021. Similarly, Holmes and Vaughan indicated that esophageal cancer incidence in higher‐income regions has plateaued or decreased due to better healthcare access and awareness programs [8].

Conversely, regions with lower SDI, such as Southern Sub‐Saharan Africa and Central Asia, experienced higher EOEC burdens. This discrepancy is in line with the observations of Vizcaino et al., who found significant geographic differences in esophageal cancer incidence, with developing regions experiencing the highest rates due to insufficient healthcare infrastructure and limited access to cancer screening programs [24]. Our study extends these findings by highlighting that while the global trend is declining, there are regions still struggling with high burdens, possibly due to a lack of healthcare resources, poor cancer awareness, and continued exposure to risk factors such as tobacco and alcohol.

Our results indicated a significant disparity between men and women, with men showing consistently higher incidence, mortality, and DALY rates than women. This finding is consistent with the work of Schneider et al., who reported a predominance of esophageal cancer in males, which they attributed to the higher prevalence of tobacco and alcohol use among men [9]. Moreover, Lagergren et al. identified that lifestyle factors such as smoking and alcohol consumption, which are more prevalent among males, are major risk factors for esophageal adenocarcinoma, further reinforcing our findings [25]. Similarly, Patel et al. also reported that men are at higher risk of developing EOEC, and our findings corroborate this pattern globally [26].

Regarding age‐specific differences, our study revealed that the incidence, mortality, and DALYs rates for EOEC increased with age within the 20–49 age group, with men in the 35–39 age group and women in the 40–44 age group showing the steepest declines. These observations align with the findings of Hur et al., who reported that younger cohorts have benefited more from risk reduction interventions, particularly in high‐income regions where public health campaigns targeting smoking cessation have been effective [27].

The inverse relationship between SDI and EOEC burden observed in this study underscores the impact of socio‐economic development on reducing disease incidence and improving survival outcomes. This observation is in agreement with Holmes and Vaughan, who showed that regions with higher socio‐economic status generally experienced lower incidence and mortality rates due to better healthcare access, higher health literacy, and greater availability of cancer screening programs [8]. The significant reduction in EOEC burden in countries like Kazakhstan and the Republic of Korea, which demonstrated the sharpest declines in ASIR, ASMR, and ASDR, suggests that sustained socio‐economic improvements and effective public health policies have positively impacted these countries [27].

However, some regions, such as Central and Eastern Europe, exhibited higher‐than‐expected EOEC burdens despite having higher SDI, a finding consistent with the work of Vaughan et al. who attributed these elevated rates to cultural and lifestyle factors, including high alcohol consumption [11]. Additionally, Castro et al. reported that high alcohol consumption in these regions has historically contributed to high esophageal cancer rates, which explains why socio‐economic improvements alone have not sufficed in reducing the burden in these areas [28].

The current study identified smoking, alcohol use, low vegetable intake, and chewing tobacco as significant contributors to the global EOEC burden. These findings align with previous research emphasizing the role of lifestyle factors in the development of esophageal cancer [26, 28]. Recent studies have highlighted that obesity, often associated with poor diet and sedentary lifestyles, is becoming an increasingly important risk factor for EOEC, especially in high‐income regions, where rising incidence trends have been observed in younger populations [29]. Similarly, Lagergren and Holmes identified smoking and alcohol use as major risk factors, particularly for esophageal adenocarcinoma, which is more prevalent in younger individuals [8, 25].

Interestingly, our findings also indicated that the proportion of DALYs attributable to chewing tobacco has increased in high‐SDI countries, whereas it has decreased in low‐SDI countries. This divergence may reflect shifts in social norms and public health policies targeting tobacco use in different regions. The Centers for Disease Control and Prevention reported similar observations, noting that while cigarette smoking has decreased due to public health efforts, the use of alternative tobacco products, such as smokeless tobacco (including chewing tobacco), has remained prevalent, particularly in certain states and demographic groups [30].

The reduction in EOEC burden attributable to dietary risks and tobacco use from 1990 to 2021 is consistent with the findings of Hur et al. and Castro et al., both of whom reported that public health initiatives promoting healthy eating and smoking cessation have successfully reduced the prevalence of risk factors for esophageal cancer in high‐income regions [27, 28]. However, as our study shows, these improvements are not uniformly distributed, and some regions, particularly those with lower SDI, continue to face high burdens due to persistent risk factors and inadequate public health interventions.

Our predictive analysis suggests that by 2050, the global burden of EOEC is expected to increase significantly, with Southern Sub‐Saharan Africa projected to have the highest incidence and mortality rates. These projections highlight the urgency of addressing the modifiable risk factors contributing to EOEC, particularly in regions facing socio‐economic challenges. The projected increase in EOEC burden is in line with the observations of Schneider et al., who emphasized the potential impact of population growth and aging, coupled with ongoing exposure to high‐risk behaviors, on the future burden of esophageal cancer [9].

The frontier analysis conducted in this study offers valuable insights into the performance of different countries in managing EOEC burden relative to their level of socio‐economic development. Similar to the findings of Pohl and Welch, our analysis revealed significant disparities between countries, with some low‐ and middle‐income countries performing better than expected based on their SDI, while others lagged behind [31]. These findings suggest that effective public health interventions and cancer control programs can significantly improve outcomes, even in countries with limited resources.

This study is distinguished by several strengths. Firstly, it provides a comprehensive assessment of the global, regional, and national burden of EOEC over a 30‐year period, utilizing the robust and standardized GBD 2021 database. This long‐term analysis allowed us to identify critical trends and disparities in EOEC burden, providing valuable insights into the evolution of this disease across different socio‐economic contexts. Secondly, the use of advanced statistical methodologies, including joinpoint regression and age‐period‐cohort models, enabled a thorough evaluation of temporal trends and the effects of age, period, and cohort on EOEC outcomes. The inclusion of frontier analysis further added depth to our understanding by comparing the performance of various countries relative to their development, highlighting both successes and areas in need of improvement. Additionally, the predictive analysis to 2050 offers forward‐looking insights, which are crucial for informing policy decisions and preparing for future healthcare needs. The stratification of data by age, sex, and SDI provides nuanced details that can guide targeted public health interventions, making the study highly relevant for healthcare policymakers and practitioners focused on reducing the EOEC burden.

Despite these strengths, our study has several limitations. The primary limitation is the reliance on GBD data, which is derived from multiple data sources and modeling techniques, leading to potential biases and inaccuracies, especially in regions with limited data availability. Underreporting and misclassification of cancer cases, particularly in low‐ and middle‐income countries, may result in underestimation of the true EOEC burden. Additionally, the GBD database only includes individuals aged 20 and above, limiting our ability to analyze EOEC in younger populations and to compare trends across the full age spectrum of esophageal cancer. Another limitation is the attribution of risk factors, which are based on existing literature and assumptions that may not fully capture the complexity of risk exposure across different regions. This could lead to inaccuracies in estimating the relative contributions of individual risk factors to EOEC burden. Moreover, the study did not consider the potential interactions between multiple overlapping risk factors, such as smoking, alcohol consumption, and dietary habits, which often co‐occur and may collectively influence disease risk. Finally, while the study explores the relationship between SDI and EOEC burden, it does not account for other social determinants of health, such as access to healthcare, education, and urbanization, which could significantly impact disease outcomes.

In conclusion, our study provides a comprehensive assessment of the global burden of EOEC from 1990 to 2021, highlighting significant declines in incidence, mortality, and DALYs, alongside marked regional and socio‐economic disparities. These findings highlight the importance of addressing modifiable risk factors and improving healthcare access in low‐SDI countries. Future research should focus on understanding regional variations and developing targeted public health interventions to reduce the EOEC burden effectively.

## Author Contributions

All authors had full access to the data in the study and take responsibility for the integrity of the data and the accuracy of the data analysis. Conceptualization: G.P. and H.J. Methodology: G.P. and Y.H. Investigation: Y.Y., S.W., and S.M. Formal analysis: G.P. and Y.H. Resources: S.W. and S.M. Writing – original draft: G.P., Y.H., and H.J. Writing – review and editing: G.P. and H.J. Visualization: J.L. Supervision: JL.

## Conflicts of Interest

The authors declare no conflicts of interest.

## Supporting information


Data S1.

## Data Availability

The data that support the findings of this study are openly available from the Global Health Data Exchange Global Burden of Disease Results Tool (https://ghdx.healthdata.org/gbd‐results‐tool).
