# Supplementary material for: Global Burden of Early‐Onset Esophageal Cancer From 1990 to 2021: A Systematic Analysis of the Global Burden of Disease Study 2021
Source: Thorac Cancer. 2025 May 21;16(10):e70082. doi: 10.1111/1759-7714.70082 (PMC12093250; doi:10.1111/1759-7714.70082)
Supplement: Supplementary file 1 — Data S1. [file TCA-16-e70082-s001.docx]

**Global, Regional, and National Burden of Early-Onset Esophageal Cancer and Associated Risk Factors from 1990 to 2021: A Systematic Analysis with Forecasts to 2050**

**Supplementary Materials**

**Table of Contents**

1. **Overview**.....................................................................................................................................................................2
2. **Data sources**................................................................................................................................................................2
3. **Estimation of mortality, incidence, and DALYs**......................................................................................................2
4. **Socio-demographic index (SDI) definition**...............................................................................................................4
5. **Standardization Methods**..........................................................................................................................................4
6. **Joinpoint regression analysis**.....................................................................................................................................5
7. **Supplementary Table 1.** Sequelae for esophageal cancer and associated disability weights from Global Burden of Disease 2021................................................................................................................................................................8
8. **Supplementary Table 2.** The Burden of Early-Onset Esophageal Cancer in 2021: Global, Regional, and SDI-Level analysis.......................................................................................................................................................8
9. **Supplementary Table 3**. Global Burden of Early-Onset Esophageal Cancer by SDI Categories and GBD Regions from 1990 to 2021, Stratified by Sex.........................................................................................................................14
10. **Supplementary Table 4**. Early-Onset Esophageal Cancer Burden by Different Countries and Territories from 1990 to 2021 ..............................................................................................................................................................21
11. **Supplementary Table 5**. Age-Specific AAPC of the Global Burden of Early-Onset Esophageal Cancer from 1990 to 2021, by SDI Categories........................................................................................................................................35
12. **Supplementary Table 6**. Frontier Analysis of SDI and ASR for Early-Onset Esophageal Cancer Across 204 Countries and Territories from 1990 to 2021.............................................................................................................40
13. **Supplementary Table 7.** Global DALYs Attributable to Level 2 Risk Factors and AAPC in 1990 and 2021, for Both Sexes..................................................................................................................................................................55
14. **Supplementary Table 8.** Proportion of Attributable Risk Factors for Early-Onset Esophageal Cancer DALYs in 1990 and 2021, by Sex...............................................................................................................................................55
15. **Supplementary Table 9**. Proportion of Attributable Risk Factors for Early-Onset Esophageal Cancer DALYs in 1990 and 2021, by SDI Category and Sex.................................................................................................................57
16. **Supplementary Table 10**. Global Age-Specific DALYs Rates and Proportional DALY Burden of Early-Onset Esophageal Cancer by Age Group and Sex in 2021..................................................................................................59
17. **Supplementary Table 11.** Forecast of Global and Regional ASIR, ASMR (per 100,000), and Total Cases for Both Sexes, 2022-2050.......................................................................................................................................................60
18. **Supplementary Figure 1**. Age-standardized incidence rates (A), mortality rates (B), and DALY rates (C) of early-onset esophageal cancer per 100,000 population in 2021, presented by countries and territories....................................................................................................................................................................62
19. **Supplementary Figure 2.** Global temporal trends of early-onset esophageal cancer (1990-2021) ........................64
20. **Supplementary Figure 3.** Joinpoint regression analysis of the temporal trends in the burden of early-onset esophageal cancer from 1990 to 2021........................................................................................................................66
21. **Supplementary Figure 4**. Hierarchical clustering of the average annual percentage changes (AAPC) in age-standardized incidence rates (ASIR) (A), age-standardized mortality rates (ASMR) (B), and age-standardized DALY rates (ASDR) (C) for early-onset esophageal cancer across the 21 Global Burden of Disease (GBD) regions from 1990 to 2021......................................................................................................................................................67
22. **Supplementary Figure 5**. Joinpoint regression analysis of age-standardized incidence rates (A), mortality rates (B), and DALY rates (C) for early-onset esophageal cancer in the top three countries for each metric from 1990 to 2021............................................................................................................................................................................68
23. **Supplementary Figure 6**. The average annual percentage change (AAPC) in age-standardized incidence rates (A), mortality rates (B), and DALY rates (C) for early-onset esophageal cancer from 1990 to 2021............................................................................................................................................................................70
24. **Supplementary Figure 7**. Age-standardized incidence rates and mortality rates for early-onset esophageal cancer across 21 GBD regions (A, B) and 204 countries and territories (C, D) by SDI (Socio-Demographic Index) from 1990 to 2021...............................................................................................................................................................73
25. **Supplementary Figure 8**. Frontier analysis based on the SDI and age-standardized mortality rate (ASMR) and Disability-Adjusted Life Years (DALYs) rate for early-onset esophageal cancer in 2021........................................75
26. **Supplementary Figure 9**. Global trends in Disability-Adjusted Life Years (DALYs) attributed to Level 2 risk factors for early-onset esophageal cancer from 1990 to 2021...................................................................................76
27. **Supplementary Figure 10.** Global maps illustrating risk-attributable Disability-Adjusted Life Year (DALY) rates (per 100,000 population) for Level 2 risk factors contributing to early-onset esophageal cancer in 2021............................................................................................................................................................................77
28. **Supplementary Figure 11.** Changes in risk factors contributing to early-onset esophageal cancer DALYs across age groups globally from 1990 to 2021.....................................................................................................................79
29. **Supplementary Figure 12**. Global trends in the proportion of Disability-Adjusted Life Years (DALYs) attributable to four risk factors for early-onset esophageal cancer, stratified by sex (men, women, and both) from 1990 to 2021...............................................................................................................................................................81
30. **Supplementary Figure 13**.Trends in the proportion of Disability-Adjusted Life Years (DALYs) attributable to four risk factors for early-onset esophageal cancer, stratified by sex and Socio-Demographic Index (SDI) from 1990 to 2021. .............................................................................................................................................................82
31. **Supplementary Figure 14**. Projected number of cases and age-standardized rates for early-onset esophageal cancer globally from 2022 to 2050, by sex, using the Bayesian Age-Period-Cohort (BAPC) model..........................................................................................................................................................................84
32. **Overview**

The Global Burden of Disease (GBD) is an analytical framework for global descriptive epidemiology that comparably weights the combined impact of fatal and non-fatal health outcomes across diseases, injuries, and risk factors. It is a systematic scientific effort to quantify health loss caused by diseases, injuries, risk factors, impairments, and etiologies, based on age, sex, and geographic location at specific time points. The Institute for Health Metrics and Evaluation (IHME) leads the GBD study, providing regular estimates of key population health indicators. GBD 2021 examines global health trends.^1^ These studies, leveraging 328,938 data sources, reveal health disparities across age, sex, location, and socioeconomic groups, highlighting the impact of COVID-19 alongside other health challenges.^2-4^

1. **Data sources**

All data for this study were extracted from the Global Health Data Exchange (http://ghdx.healthdata.org/gbd-results-tool), including:

1. Global age- and sex-specific incidence, mortality, DALYs, and crude rates (per 100,000 persons) from 1990 to 2021;
2. Regional age- and sex-specific data by SDI categories;
3. National age- and sex-specific data;
4. The GBD 2021 world standard population;
5. Esophageal cancer DALYs and crude rates by age, sex, and risk factors (level 2 and level 3).
6. **Estimation of mortality, incidence, and DALYs**

The Global Burden of Disease (GBD) study uses a comprehensive approach to model cancer mortality rates by aggregating and processing data from vital registration systems, verbal autopsies, and cancer registries. This process includes standardizing data formats, recalculating subtotals, mapping to GBD causes, splitting by age and sex, disaggregating causes, redistributing "garbage codes," and removing duplicates. Mortality-to-incidence ratios (MIRs) are estimated through a three-step modeling process using spatiotemporal Gaussian process regression (ST-GPR), incorporating covariates such as sex, age group, and the Healthcare Access and Quality (HAQ) Index. For rare cancers, negative binomial regression is employed to account for overdispersion in the data. The GBD model also adjusts for biases such as underreporting in low-income countries or cancer type misclassification. Additionally, specific cancers, like liver cancer, use DisMod-MR 2.1 models to apportion cases by etiology. The final mortality estimates are derived by applying the estimated MIRs to the cleaned incidence data, ensuring consistency with all-cause mortality figures. This robust ensemble modeling approach enables cross-validation and determination of uncertainty intervals, ensuring reliable and globally consistent cancer mortality estimates.

To estimate incidence, the final GBD cancer mortality estimates (except for retinoblastoma) are converted into incidence estimates by using cancer-specific MIRs. Mortality estimates at the 1000-draw level are divided by the modeled MIRs at the same 1000-draw level, generating 1000 draws of incidence estimates, which provide the estimated mean incidence with a 95% uncertainty interval. The assumption is that the uncertainty in MIRs is independent of uncertainty in the mortality estimates.

To estimate DALYs, GBD 2021 begins by estimating cause-specific mortality and nonfatal health loss. For each year in which Years Lived with Disability (YLDs) are estimated, GBD 2021 calculates Disability-Adjusted Life Years (DALYs) by summing Years of Life Lost (YLLs) and YLDs for each age, sex, and location (Figure 1). It is assumed that uncertainty in YLLs is independent of uncertainty in YLDs. GBD 2021 computes 500 draws for DALYs by summing the corresponding draws for YLLs and YLDs, repeating this for each subsequent draw. The 95% uncertainty intervals (UIs) are determined using the 25th and 975th ordered draws of the DALY uncertainty distribution. Finally, GBD 2021 calculates DALYs as the sum of YLLs and YLDs for each cause, location, age group, sex, and year.

Figure 1. DALYs burden estimation for GBD 2021


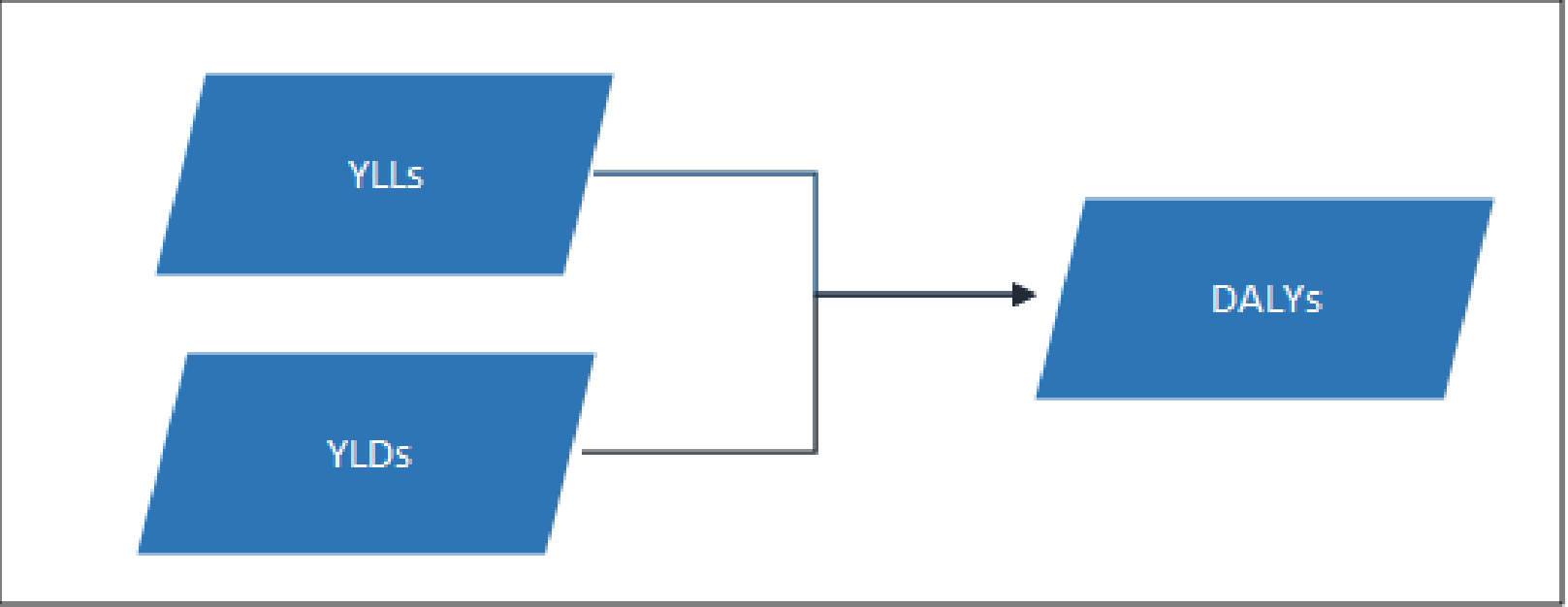


1. **Socio-demographic index (SDI) definition**

The Socio-demographic Index (SDI) is a composite measure of social and economic factors that influence health outcomes. It is calculated as the geometric mean of indices for total fertility rate, mean education level, and income per capita. SDI values range from 0 to 100, with locations divided into five quintiles: low, low-middle, middle, high-middle, and high SDI. Details can be seen on the page 1385-1448, Appendix 1 of the reference 5.

**SDI Reference Quintile Values**

| location_name | Lower bound | Upper bound |
| --- | --- | --- |
| Low SDI | 0.000 | 0.466 |
| Low-middle SDI | 0.466 | 0.619 |
| Middle SDI | 0.619 | 0.712 |
| High-middle SDI | 0.712 | 0.810 |
| High SDI | 0.810 | 1.000 |

1. **Standardization Methods**

To enable comparison of disease burden across years and regions, age-standardization was necessary to account for differences in population age structures. Although GBD 2021 provides age-standardized rates (ASR), this study recalculated age-specific ASR to ensure comparability of statistical indicators across populations. The direct standardization method was used,^6^ calculated with the formula:

$$ASR=\frac{\sum_{i=1}^{A} \alpha_{i}w_{i}}{\sum_{i=1}^{A} w_{i}}$$

Where *a_i_* is the age-specific rate in the *i^th^* age group and *w_i_* is the corresponding weight in the same age group of the reference standard population.

1. **Joinpoint regression analysis**
2. **Model Introduction**

The Joinpoint regression model includes two primary types: linear (y = xb) and log-linear (ln y = xb). The choice of model depends on the distribution of the dependent variable and data characteristics.

- Linear Model: Used when the dependent variable follows a normal distribution with a large sample size.
- Log-linear Model: Preferred when the dependent variable follows an exponential or Poisson distribution.

1. **Modeling Method**

The Grid Search Method (GSM) is the default approach used by Joinpoint software for modeling. GSM divides the study data into a grid, where each intersection represents a potential scenario. It then calculates performance metrics, such as the sum of squared errors (SSE) and mean squared errors (MSE), for the equations corresponding to each grid point within specified intervals using a fixed step size. The core purpose of using GSM in the Joinpoint model is to establish all possible segment function joinpoints and compute SSE and MSE for each potential scenario. The grid point with the smallest MSE is selected as the optimal joinpoint for the segment function, and parameters such as β0, β1, δ1, ..., δk are fitted according to the selected joinpoints and their respective interval functions. This method enables the identification of the optimal model that best describes the data trend in terms of the number and location of change points (joinpoints).^7^

1. **Model Optimization**

The Monte Carlo permutation test is the default method used for model optimization in Joinpoint software. Before modeling, it is necessary to define the range for the number of potential joinpoints, denoted as k,where *k∈(MIN, MAX)*. Here, MIN is typically set to 0 (the minimum number of joinpoints), and MAX represents the maximum number of joinpoints. Each permutation test evaluates the null hypothesis *H_0_*: the number of joinpoints is *k = k_a_*, against the alternative hypothesis *H_1_*: the number of joinpoints is *k = k_b_*. The testing process begins with *k_a_* = *MIN* and *k_b_* = *MAX*. If *H_0_* is rejected, *k* is increased to *k_a_ + 1* for further testing; if *H_0_* is not rejected, *k* is decreased to *k_b_ - 1* for another test. This iterative process continues until *k_a_* equals *k_b_*, indicating that *k = k_a_ = k_b_* is the preferred number of joinpoints selected by the permutation test, and the corresponding model is considered the optimal one.^8^

1. **Index Calculation**

Annual Percent Change (APC) and Average Annual Percent Change (AAPC), along with their 95% confidence intervals (CI), are the primary outcome measures in the Joinpoint model. As the name suggests, APC represents the yearly percentage change in the dependent variable. For example, in a log linear model, ln (*y*) =*β0 + β1 x*， where *y* represents the incidence rate and *x* represents the year of incidence, the formula for calculating APC in the fitted model is derived as follows:

$$APC=\left[\frac{\text{ y}_{\text{x}+1}\text{ - y}_{\text{x}}}{\text{ y}_{\text{x}}}\right]\times100=\left(e^{\beta1}-1\right)\times100$$

The lower and upper limits of the 100(1-α) % confidence interval are respectively:

$$APC_{U\left(\alpha\right)}=100\left(e^{\beta_{1}+s\times t_{d}^{-1}\left(1-\alpha/2\right)}-1\right)$$

In the formula above, *β*1 represents the regression coefficient, *s* is the standard error of *β*1, *d* is the degrees of freedom, and *t_d_(q)* corresponds to the qth percentile of the t-distribution with d degrees of freedom (e.g., 95%).

The APC is used to evaluate the internal trend within each independent interval of a segmented function or the overall trend when no connecting points are present. When assessing the overall average change trend across multiple intervals, the AAPC is required. The calculation of AAPC involves a weighted average of the regression coefficients for each interval, with the weights based on the width *w* of the segment intervals. The formula for AAPC is as follows:

$$AAPC=\left(e^{\sum\omega i\beta i/\sum\omega i}-1\right)\times100$$

The lower and upper limits of the 100(1-α)% confidence interval are, respectively:

$$AAPC_{L\left(\alpha\right)}=\left\{\exp\left[\ln\left(\left(AAPC/100\right)+1\right)-Z_{1-\alpha/2}\sqrt{\sum\mathop{w_{i}}\limits^{\sim2} \mathop{\sigma_{i}}\limits^{\sim2} }\right]-1\right\}$$

$$AAPC_{U\left(\alpha\right)}=\left\{\text{exp}\left[\ln\left(\left(AAPC/100\right)+1\right)-Z_{1-\alpha/2}\sqrt{\sum\mathop{w_{i}}\limits^{\sim2} \mathop{\sigma_{i}}\limits^{\sim2} }\right]-1\right\}$$

In the formula mentioned above, *w*_i_ represents the width of each segment function interval, which refers to number of years included in the interval. *β_i_* denotes the regression coefficient for each interval, *σ^2^i* is the variance of *β_i_*, and *Z_α_* represents the value corresponding to the *α* percentile in the normal distribution.

**Reference:**

1. Institute for Health Metrics and Evaluation (IHME). Global Burden of Disease 2021: findings from the GBD 2021 study. Seattle, WA: IHME, 2024.
2. GBD 2021 Diseases and Injuries Collaborators. Global incidence, prevalence, years lived with disability (YLDs), disability-adjusted life-years (DALYs), and healthy life expectancy (HALE) for 371 diseases and injuries in 204 countries and territories and 811 subnational locations, 1990-2021: a systematic analysis for the Global Burden of Disease Study 2021. Lancet 2024;403:2133-61.
3. GBD 2021 Risk Factors Collaborators. Global burden and strength of evidence for 88 risk factors in 204 countries and 811 subnational locations, 1990-2021: a systematic analysis for the Global Burden of Disease Study 2021. Lancet 2024;403:2162-2203.
4. GBD 2021 Causes of Death Collaborators. Global burden of 288 causes of death and life expectancy decomposition in 204 countries and territories and 811 subnational locations, 1990-2021: a systematic analysis for the Global Burden of Disease Study 2021. Lancet 2024;403:2100-32.
5. GBD 2021 Diseases and Injuries Collaborators. Global incidence, prevalence, years lived with disability (YLDs), disability-adjusted life-years (DALYs), and healthy life expectancy (HALE) for 371 diseases and injuries in 204 countries and territories and 811 subnational locations, 1990-2021: a systematic analysis for the Global Burden of Disease Study 2021. Lancet 2024;403:2133-61.
6. Inskip H. Standardization methods. In: Armitage P, Colton T, eds. Encyclopedia of biostatistics. Chichester: John Wiley & Sons, 2005.
7. Kim S, Lee S, Choi JI, Cho H. Binary genetic algorithm for optimal Joinpoint detection: application to cancer trend analysis. Stat Med 2021;40:799-822.
8. Yang JJ, Trucco EM, Buu A. A hybrid method of the sequential Monte Carlo and the Edgeworth expansion for computation of very small p-values in permutation tests. Stat Methods Med Res 2019;28:2937-51.

**Supplementary Table 1: Sequelae for esophageal cancer and associated disability weights from Global Burden of Disease 2021**

| **Sequela** | **Health State Name** | **Health State Lay Description** | **Disability weight (95% CI)** |
| --- | --- | --- | --- |
| Diagnosis and primary therapy phase | Cancer, diagnosis and primary therapy | Has pain, nausea, fatigue, weight loss and high anxiety. | 0.288  (0.193-0.399) |
| Controlled phase | Generic uncomplicated disease: worry and daily medication | Has a chronic disease that requires medication every day and causes some worry but minimal interference with daily activities. | 0.049  (0.031-0.072) |
| Metastatic phase | Cancer, metastatic | Has severe pain, extreme fatigue, weight loss and high anxiety. | 0.451  (0.307-0.600) |
| Terminal phase | Terminal phase, with medication (for cancers, end-stage kidney/liver disease) | Has lost a lot of weight and regularly uses strong medication to avoid constant pain. The person has no appetite, feels nauseous, and needs to spend most of the day in bed. | 0.540  (0.377-0.687) |

CI=confidence interval.

**Supplementary Table 2. The Burden of Early-Onset Esophageal Cancer in 2021: Global, Regional, and SDI-Level Analysis**

| **Locations** | **Both sexes** | | | **Women** | | | **Men** | | |
| --- | --- | --- | --- | --- | --- | --- | --- | --- | --- |
|  | **Incidence** | **Deaths** | **DALYs** | **Incidence** | **Deaths** | **DALYs** | **Incidence** | **Deaths** | **DALYs** |
|  | **Number of cases in 2021 (95% UI)** | | | | | | | | |
| Global | 42698.14 (37736.66-48265.84) | 32921.65 (29239.29-37214.65) | 1551915.12 (1378422.37-1752980.35) | 9911.05 (8345.28-11904.20) | 7619.84 (6455.46-9250.80) | 367041.38 (310509.23-446596.77) | 32787.09 (28124.42-38002.18) | 25301.81 (21875.95-29176.66) | 1184873.75 (1026014.38-1364581.58) |
| High SDI | 4427.90 (4200.99-4653.39) | 2783.59 (2640.71-2934.82) | 130325.21 (123674.10-137383.76) | 805.79 (762.08-871.96) | 422.66 (398.62-467.52) | 20039.04 (18877.95-22199.64) | 3622.11 (3406.96-3842.02) | 2360.94 (2223.69-2508.51) | 110286.18 (103999.10-117182.75) |
| High-middle SDI | 11556.00 (9225.14-14567.46) | 8017.99 (6429.34-10085.26) | 372791.84 (299012.33-468352.81) | 1314.04 (1025.06-1688.11) | 802.97 (649.77-1003.32) | 37990.66 (30665.35-47418.00) | 10241.97 (7936.66-13202.13) | 7215.02 (5660.00-9261.65) | 334801.18 (262501.37-429321.73) |
| Middle SDI | 15321.46 (12918.85-18150.08) | 11796.82 (10020.04-13928.89) | 554398.57 (471878.18-652498.65) | 3186.92 (2516.88-3825.97) | 2266.60 (1837.64-2664.21) | 108184.73 (87611.12-127077.99) | 12134.54 (9800.34-14938.30) | 9530.22 (7776.73-11563.06) | 446213.84 (365135.72-539842.12) |
| Low-middle SDI | 7198.22 (6361.13-8507.72) | 6478.57 (5718.14-7695.53) | 310181.93 (273424.96-369663.20) | 2900.78 (2379.72-3973.25) | 2579.37 (2109.93-3538.07) | 125235.96 (102064.47-172643.47) | 4297.44 (3783.49-4902.13) | 3899.21 (3427.76-4443.77) | 184945.97 (162753.01-210665.63) |
| Low SDI | 4174.95 (3447.39-5006.69) | 3827.91 (3157.45-4605.60) | 183433.21 (151073.19-221215.53) | 1698.91 (1194.86-2237.77) | 1544.37 (1083.65-2042.71) | 75404.03 (52979.85-100031.32) | 2476.03 (2022.93-3031.53) | 2283.54 (1861.98-2793.23) | 108029.18 (88087.09-132216.24) |
| Central Asia | 365.16 (313.16-423.00) | 331.32 (284.64-383.75) | 15997.42 (13729.37-18579.03) | 168.62 (138.49-204.55) | 151.64 (124.62-183.87) | 7368.39 (6041.50-8954.47) | 196.53 (169.95-226.01) | 179.68 (155.26-206.41) | 8629.03 (7445.94-9933.59) |
| Central Europe | 353.42 (318.06-391.19) | 308.65 (278.35-341.37) | 14122.01 (12747.04-15617.06) | 47.74 (42.82-53.47) | 39.75 (35.67-44.35) | 1850.94 (1660.18-2064.97) | 305.67 (271.54-341.36) | 268.90 (239.42-300.21) | 12271.07 (10926.11-13691.04) |
| Eastern Europe | 878.03 (781.96-983.92) | 704.36 (629.44-788.67) | 32595.41 (29159.45-36457.17) | 123.19 (107.65-139.03) | 95.94 (83.84-108.06) | 4529.59 (3963.44-5100.92) | 754.85 (660.64-859.76) | 608.43 (533.63-692.54) | 28065.82 (24632.10-31935.93) |
| Australasia | 92.47 (76.97-110.57) | 64.97 (53.97-78.31) | 3032.22 (2518.53-3660.15) | 18.36 (14.64-22.94) | 10.53 (8.54-13.00) | 496.44 (402.20-613.86) | 74.11 (59.36-92.50) | 54.43 (43.63-67.50) | 2535.78 (2030.08-3149.14) |
| High-income Asia Pacific | 578.69 (531.21-632.43) | 242.28 (225.98-263.31) | 11183.03 (10429.61-12165.54) | 151.69 (137.81-165.92) | 60.53 (56.90-65.17) | 2820.21 (2643.62-3038.32) | 426.99 (382.58-479.00) | 181.76 (166.41-202.63) | 8362.82 (7660.83-9345.44) |
| High-income North America | 1283.11 (1225.81-1341.47) | 830.14 (795.63-865.87) | 39411.91 (37762.91-41116.55) | 271.26 (255.55-288.21) | 136.75 (130.07-144.25) | 6559.71 (6236.21-6935.07) | 1011.85 (958.83-1065.48) | 693.39 (658.66-727.65) | 32852.20 (31212.94-34488.97) |
| Southern Latin America | 165.67 (142.03-193.69) | 136.40 (116.38-159.55) | 6455.83 (5506.24-7550.63) | 46.74 (38.23-56.38) | 36.04 (29.61-43.44) | 1720.02 (1414.49-2072.99) | 118.93 (97.43-144.00) | 100.36 (81.87-121.92) | 4735.81 (3862.58-5758.29) |
| Western Europe | 1546.16 (1445.90-1660.18) | 966.52 (910.73-1028.90) | 45063.74 (42413.81-48041.94) | 297.29 (274.99-320.80) | 163.09 (153.33-173.76) | 7670.79 (7214.13-8179.14) | 1248.87 (1153.88-1357.02) | 803.43 (748.24-864.54) | 37392.95 (34837.26-40300.59) |
| Andean Latin America | 63.98 (48.03-85.50) | 55.53 (41.54-73.77) | 2702.62 (2027.40-3583.21) | 20.66 (14.52-29.22) | 17.34 (12.15-24.43) | 842.05 (590.62-1185.52) | 43.32 (31.30-59.71) | 38.19 (27.57-52.71) | 1860.56 (1345.68-2563.58) |
| Caribbean | 172.01 (142.01-208.52) | 150.17 (124.19-183.20) | 6966.91 (5749.55-8510.79) | 34.50 (25.19-46.34) | 29.71 (21.61-40.50) | 1424.00 (1029.02-1945.62) | 137.52 (112.63-169.29) | 120.46 (98.32-148.98) | 5542.91 (4519.71-6874.85) |
| Central Latin America | 368.34 (326.05-416.18) | 319.81 (282.71-361.37) | 15546.77 (13751.29-17527.40) | 97.47 (81.48-113.06) | 81.45 (67.98-94.49) | 3982.11 (3324.26-4621.95) | 270.87 (234.85-311.76) | 238.36 (206.73-273.98) | 11564.66 (10057.37-13260.31) |
| Tropical Latin America | 1380.73 (1277.33-1486.07) | 1214.54 (1124.75-1306.73) | 56931.58 (52745.85-61271.06) | 225.50 (207.17-244.25) | 190.36 (175.54-206.34) | 8996.34 (8290.76-9750.38) | 1155.23 (1050.79-1256.37) | 1024.19 (932.59-1112.31) | 47935.24 (43669.65-52088.59) |
| North Africa and Middle East | 1158.15 (923.11-1408.32) | 989.43 (778.59-1205.72) | 47596.45 (37437.99-57968.88) | 521.81 (305.80-685.44) | 437.07 (243.30-582.31) | 21115.94 (11771.87-28092.96) | 636.34 (535.91-760.14) | 552.37 (464.77-661.84) | 26480.51 (22311.45-31711.46) |
| South Asia | 7131.69 (6145.57-8684.63) | 6395.67 (5506.97-7771.79) | 306269.74 (263514.68-373940.46) | 3241.62 (2622.93-4553.99) | 2870.39 (2310.98-4052.59) | 138776.91 (111421.28-196770.45) | 3890.08 (3318.77-4518.19) | 3525.29 (3005.41-4092.20) | 167492.84 (142720.60-194218.98) |
| East Asia | 19779.66 (15474.84-24890.89) | 13630.64 (10587.28-17360.69) | 634795.32 (493917.53-807771.32) | 2260.94 (1597.17-3092.69) | 1186.61 (864.94-1624.81) | 56052.72 (40795.20-76677.41) | 17518.72 (13162.49-22522.62) | 12444.03 (9378.82-16086.46) | 578742.60 (436167.79-747674.59) |
| Oceania | 20.87 (14.71-29.65) | 18.70 (13.24-26.63) | 920.08 (649.44-1314.07) | 5.57 (3.37-9.57) | 4.97 (3.03-8.64) | 254.62 (155.00-439.45) | 15.30 (10.46-22.77) | 13.72 (9.38-20.61) | 665.46 (453.03-1000.29) |
| Southeast Asia | 2039.41 (1650.56-2501.18) | 1693.70 (1381.57-2071.24) | 80190.22 (65448.85-98104.44) | 561.12 (419.29-706.05) | 462.51 (344.97-587.13) | 22524.16 (16728.27-28593.00) | 1478.28 (1144.71-1886.01) | 1231.19 (965.95-1559.35) | 57666.06 (45242.66-73035.41) |
| Central Sub-Saharan Africa | 643.07 (425.58-900.22) | 591.33 (389.06-828.28) | 28037.41 (18410.95-39327.22) | 224.28 (101.46-361.90) | 204.57 (92.05-333.59) | 9923.27 (4468.62-16176.64) | 418.79 (269.40-618.11) | 386.76 (247.91-564.84) | 18114.14 (11598.87-26529.99) |
| Eastern Sub-Saharan Africa | 2729.72 (2174.63-3411.55) | 2501.08 (1985.96-3135.20) | 119795.34 (94934.37-150933.80) | 1090.12 (714.19-1701.95) | 990.52 (645.48-1551.74) | 48485.26 (31601.96-76332.13) | 1639.60 (1287.47-2089.68) | 1510.56 (1187.73-1938.53) | 71310.08 (56010.43-91640.66) |
| Southern Sub-Saharan Africa | 893.73 (759.26-1043.85) | 806.75 (683.85-942.97) | 38137.02 (32328.99-44648.86) | 283.00 (224.59-363.94) | 250.78 (198.31-324.34) | 12020.51 (9487.30-15588.15) | 610.73 (510.09-734.53) | 555.97 (465.08-668.17) | 26116.51 (21825.61-31454.28) |
| Western Sub-Saharan Africa | 1054.06 (755.96-1308.82) | 969.64 (700.30-1209.12) | 46164.09 (33218.85-57606.60) | 219.57 (91.83-319.27) | 199.28 (82.92-289.91) | 9627.39 (4026.01-14047.83) | 834.49 (642.93-1058.27) | 770.36 (594.84-974.66) | 36536.71 (28163.47-46225.77) |
| **Crude rate in 2021 (95% UI)** | | | | | | | | | |
| Global | 1.28 (1.14-1.45) | 0.99 (0.88-1.12) | 46.68 (41.46-52.73) | 0.60 (0.51-0.72) | 0.46 (0.39-0.56) | 22.31 (18.87-27.15) | 1.95 (1.67-2.26) | 1.51 (1.30-1.74) | 70.55 (61.09-81.25) |
| High SDI | 1.00 (0.95-1.05) | 0.63 (0.60-0.66) | 29.48 (27.98-31.08) | 0.38 (0.36-0.41) | 0.20 (0.19-0.22) | 9.36 (8.82-10.37) | 1.59 (1.49-1.69) | 1.04 (0.98-1.10) | 48.38 (45.62-51.40) |
| High-middle SDI | 2.07 (1.66-2.61) | 1.44 (1.15-1.81) | 66.92 (53.67-84.07) | 0.49 (0.38-0.62) | 0.30 (0.24-0.37) | 14.03 (11.33-17.52) | 3.58 (2.77-4.61) | 2.52 (1.98-3.23) | 116.90 (91.65-149.90) |
| Middle SDI | 1.43 (1.20-1.69) | 1.10 (0.93-1.30) | 51.68 (43.99-60.83) | 0.60 (0.47-0.72) | 0.43 (0.35-0.50) | 20.39 (16.51-23.95) | 2.24 (1.81-2.76) | 1.76 (1.43-2.13) | 82.31 (67.36-99.58) |
| Low-middle SDI | 0.87 (0.76-1.02) | 0.78 (0.69-0.93) | 37.29 (32.88-44.45) | 0.70 (0.57-0.96) | 0.62 (0.51-0.85) | 30.11 (24.54-41.51) | 1.03 (0.91-1.18) | 0.94 (0.82-1.07) | 44.48 (39.14-50.66) |
| Low SDI | 1.00 (0.82-1.20) | 0.91 (0.75-1.10) | 43.84 (36.11-52.87) | 0.80 (0.56-1.05) | 0.73 (0.51-0.96) | 35.46 (24.91-47.04) | 1.20 (0.98-1.47) | 1.11 (0.90-1.36) | 52.51 (42.81-64.26) |
| Central Asia | 0.87 (0.75-1.01) | 0.79 (0.68-0.92) | 38.26 (32.84-44.44) | 0.81 (0.66-0.98) | 0.73 (0.60-0.88) | 35.26 (28.91-42.85) | 0.94 (0.81-1.08) | 0.86 (0.74-0.99) | 41.26 (35.60-47.50) |
| Central Europe | 0.75 (0.68-0.84) | 0.66 (0.59-0.73) | 30.15 (27.22-33.35) | 0.21 (0.19-0.23) | 0.17 (0.16-0.19) | 8.08 (7.25-9.01) | 1.28 (1.14-1.43) | 1.12 (1.00-1.26) | 51.30 (45.68-57.24) |
| Eastern Europe | 1.03 (0.91-1.15) | 0.82 (0.74-0.92) | 38.12 (34.10-42.63) | 0.29 (0.25-0.32) | 0.22 (0.19-0.25) | 10.53 (9.21-11.86) | 1.78 (1.55-2.02) | 1.43 (1.26-1.63) | 66.06 (57.98-75.17) |
| Australasia | 0.73 (0.61-0.88) | 0.51 (0.43-0.62) | 24.01 (19.94-28.98) | 0.29 (0.23-0.36) | 0.17 (0.13-0.21) | 7.83 (6.34-9.68) | 1.18 (0.94-1.47) | 0.87 (0.69-1.07) | 40.32 (32.28-50.07) |
| High-income Asia Pacific | 0.83 (0.76-0.91) | 0.35 (0.32-0.38) | 16.01 (14.93-17.42) | 0.45 (0.41-0.49) | 0.18 (0.17-0.19) | 8.30 (7.78-8.94) | 1.19 (1.07-1.34) | 0.51 (0.46-0.56) | 23.32 (21.36-26.05) |
| High-income North America | 0.89 (0.85-0.93) | 0.57 (0.55-0.60) | 27.23 (26.09-28.41) | 0.38 (0.35-0.40) | 0.19 (0.18-0.20) | 9.07 (8.62-9.59) | 1.40 (1.32-1.47) | 0.96 (0.91-1.01) | 45.38 (43.11-47.64) |
| Southern Latin America | 0.56 (0.48-0.65) | 0.46 (0.39-0.54) | 21.76 (18.56-25.45) | 0.31 (0.26-0.38) | 0.24 (0.20-0.29) | 11.49 (9.45-13.85) | 0.81 (0.66-0.98) | 0.68 (0.56-0.83) | 32.20 (26.26-39.15) |
| Western Europe | 0.94 (0.88-1.01) | 0.59 (0.55-0.62) | 27.32 (25.72-29.13) | 0.36 (0.34-0.39) | 0.20 (0.19-0.21) | 9.38 (8.83-10.01) | 1.50 (1.39-1.63) | 0.97 (0.90-1.04) | 44.95 (41.88-48.45) |
| Andean Latin America | 0.22 (0.16-0.29) | 0.19 (0.14-0.25) | 9.19 (6.90-12.19) | 0.14 (0.10-0.20) | 0.12 (0.08-0.17) | 5.70 (4.00-8.03) | 0.30 (0.21-0.41) | 0.26 (0.19-0.36) | 12.71 (9.20-17.52) |
| Caribbean | 0.85 (0.70-1.03) | 0.74 (0.62-0.91) | 34.51 (28.48-42.16) | 0.34 (0.25-0.46) | 0.29 (0.21-0.40) | 14.00 (10.12-19.13) | 1.37 (1.12-1.69) | 1.20 (0.98-1.49) | 55.34 (45.13-68.64) |
| Central Latin America | 0.33 (0.29-0.37) | 0.29 (0.25-0.32) | 13.96 (12.35-15.74) | 0.17 (0.14-0.20) | 0.14 (0.12-0.16) | 6.94 (5.79-8.05) | 0.50 (0.44-0.58) | 0.44 (0.38-0.51) | 21.44 (18.65-24.59) |
| Tropical Latin America | 1.33 (1.23-1.44) | 1.17 (1.09-1.26) | 55.04 (50.99-59.24) | 0.43 (0.39-0.46) | 0.36 (0.33-0.39) | 17.12 (15.78-18.56) | 2.27 (2.06-2.47) | 2.01 (1.83-2.19) | 94.19 (85.80-102.35) |
| North Africa and Middle East | 0.41 (0.33-0.50) | 0.35 (0.28-0.43) | 16.93 (13.32-20.62) | 0.39 (0.23-0.51) | 0.33 (0.18-0.44) | 15.80 (8.81-21.02) | 0.43 (0.36-0.52) | 0.37 (0.32-0.45) | 17.95 (15.13-21.50) |
| South Asia | 0.86 (0.74-1.05) | 0.77 (0.66-0.94) | 36.89 (31.74-45.04) | 0.79 (0.64-1.11) | 0.70 (0.57-0.99) | 33.93 (27.24-48.11) | 0.92 (0.79-1.07) | 0.84 (0.71-0.97) | 39.76 (33.88-46.11) |
| East Asia | 3.24 (2.53-4.07) | 2.23 (1.73-2.84) | 103.91 (80.85-132.23) | 0.77 (0.54-1.05) | 0.40 (0.29-0.55) | 19.01 (13.83-26.00) | 5.54 (4.17-7.13) | 3.94 (2.97-5.09) | 183.14 (138.02-236.60) |
| Oceania | 0.36 (0.25-0.51) | 0.32 (0.23-0.46) | 15.95 (11.26-22.78) | 0.20 (0.12-0.34) | 0.17 (0.11-0.30) | 8.93 (5.44-15.42) | 0.52 (0.36-0.78) | 0.47 (0.32-0.71) | 22.80 (15.52-34.27) |
| Southeast Asia | 0.65 (0.53-0.80) | 0.54 (0.44-0.66) | 25.52 (20.83-31.23) | 0.36 (0.27-0.45) | 0.30 (0.22-0.38) | 14.48 (10.75-18.38) | 0.93 (0.72-1.19) | 0.78 (0.61-0.98) | 36.36 (28.53-46.05) |
| Central Sub-Saharan Africa | 1.28 (0.85-1.79) | 1.18 (0.77-1.65) | 55.72 (36.59-78.15) | 0.89 (0.40-1.43) | 0.81 (0.36-1.32) | 39.29 (17.69-64.05) | 1.67 (1.07-2.47) | 1.54 (0.99-2.25) | 72.27 (46.27-105.84) |
| Eastern Sub-Saharan Africa | 1.70 (1.36-2.13) | 1.56 (1.24-1.96) | 74.76 (59.24-94.19) | 1.32 (0.87-2.06) | 1.20 (0.78-1.88) | 58.75 (38.29-92.49) | 2.11 (1.66-2.69) | 1.94 (1.53-2.49) | 91.76 (72.07-117.92) |
| Southern Sub-Saharan Africa | 2.48 (2.11-2.90) | 2.24 (1.90-2.62) | 106.02 (89.87-124.12) | 1.56 (1.24-2.01) | 1.38 (1.09-1.79) | 66.30 (52.33-85.98) | 3.42 (2.86-4.12) | 3.12 (2.61-3.75) | 146.38 (122.33-176.30) |
| Western Sub-Saharan Africa | 0.60 (0.43-0.75) | 0.55 (0.40-0.69) | 26.30 (18.93-32.82) | 0.24 (0.10-0.35) | 0.22 (0.09-0.31) | 10.44 (4.36-15.23) | 1.00 (0.77-1.27) | 0.93 (0.71-1.17) | 43.89 (33.83-55.52) |
| **Age-standardized rate in 2021 (95% UI)** | | | | | | | | | |
| Global | 1.24 (1.09-1.40) | 0.95 (0.85-1.08) | 45.07 (40.03-50.89) | 0.58 (0.49-0.70) | 0.45 (0.38-0.54) | 21.58 (18.26-26.29) | 1.88 (1.62-2.18) | 1.45 (1.26-1.68) | 68.24 (59.12-78.56) |
| High SDI | 0.85 (0.81-0.90) | 0.54 (0.51-0.57) | 25.35 (24.06-26.73) | 0.32 (0.30-0.35) | 0.17 (0.16-0.19) | 8.09 (7.61-8.96) | 1.36 (1.28-1.44) | 0.89 (0.83-0.94) | 41.74 (39.37-44.35) |
| High-middle SDI | 1.72 (1.38-2.17) | 1.19 (0.96-1.50) | 56.10 (45.03-70.42) | 0.40 (0.31-0.52) | 0.25 (0.20-0.31) | 11.81 (9.52-14.73) | 3.01 (2.34-3.89) | 2.12 (1.66-2.72) | 99.32 (77.88-127.33) |
| Middle SDI | 1.32 (1.11-1.56) | 1.01 (0.86-1.20) | 47.96 (40.87-56.37) | 0.55 (0.44-0.67) | 0.39 (0.32-0.46) | 18.97 (15.36-22.28) | 2.07 (1.68-2.55) | 1.63 (1.33-1.97) | 76.64 (62.79-92.59) |
| Low-middle SDI | 0.94 (0.83-1.11) | 0.85 (0.75-1.00) | 40.22 (35.48-47.85) | 0.75 (0.62-1.03) | 0.67 (0.55-0.91) | 32.20 (26.28-44.29) | 1.13 (0.99-1.29) | 1.02 (0.90-1.17) | 48.27 (42.48-54.98) |
| Low SDI | 1.23 (1.02-1.47) | 1.13 (0.93-1.36) | 53.30 (43.98-64.15) | 0.98 (0.69-1.29) | 0.89 (0.62-1.17) | 42.64 (29.91-56.40) | 1.48 (1.21-1.82) | 1.37 (1.12-1.67) | 64.01 (52.19-78.34) |
| Central Asia | 0.89 (0.76-1.03) | 0.81 (0.69-0.93) | 38.74 (33.26-44.98) | 0.81 (0.66-0.98) | 0.73 (0.60-0.88) | 35.15 (28.83-42.68) | 0.97 (0.84-1.12) | 0.89 (0.77-1.02) | 42.52 (36.72-48.91) |
| Central Europe | 0.58 (0.52-0.64) | 0.50 (0.45-0.56) | 23.29 (21.03-25.74) | 0.16 (0.15-0.18) | 0.13 (0.12-0.15) | 6.39 (5.73-7.13) | 0.98 (0.87-1.09) | 0.86 (0.77-0.96) | 39.69 (35.36-44.26) |
| Eastern Europe | 0.83 (0.74-0.93) | 0.67 (0.60-0.75) | 30.98 (27.72-34.64) | 0.23 (0.20-0.26) | 0.18 (0.16-0.20) | 8.54 (7.48-9.62) | 1.48 (1.29-1.68) | 1.19 (1.04-1.35) | 54.99 (48.27-62.57) |
| Australasia | 0.66 (0.55-0.79) | 0.46 (0.38-0.56) | 21.67 (17.98-26.18) | 0.26 (0.21-0.32) | 0.15 (0.12-0.18) | 7.01 (5.67-8.67) | 1.07 (0.85-1.33) | 0.78 (0.63-0.97) | 36.74 (29.38-45.69) |
| High-income Asia Pacific | 0.59 (0.54-0.65) | 0.25 (0.23-0.27) | 11.62 (10.82-12.65) | 0.33 (0.29-0.36) | 0.13 (0.12-0.14) | 6.10 (5.70-6.59) | 0.85 (0.76-0.96) | 0.36 (0.33-0.40) | 16.91 (15.46-18.93) |
| High-income North America | 0.81 (0.78-0.85) | 0.52 (0.50-0.55) | 25.08 (24.03-26.17) | 0.34 (0.32-0.36) | 0.17 (0.16-0.18) | 8.30 (7.89-8.78) | 1.29 (1.23-1.36) | 0.89 (0.84-0.93) | 42.19 (40.09-44.30) |
| Southern Latin America | 0.53 (0.46-0.62) | 0.44 (0.37-0.51) | 20.82 (17.76-24.35) | 0.30 (0.24-0.36) | 0.23 (0.19-0.27) | 10.92 (8.98-13.16) | 0.78 (0.64-0.94) | 0.66 (0.54-0.80) | 31.12 (25.37-37.84) |
| Western Europe | 0.76 (0.71-0.82) | 0.48 (0.45-0.51) | 22.46 (21.14-23.95) | 0.29 (0.27-0.32) | 0.16 (0.15-0.17) | 7.72 (7.26-8.23) | 1.23 (1.13-1.33) | 0.79 (0.73-0.85) | 37.21 (34.66-40.12) |
| Andean Latin America | 0.23 (0.17-0.30) | 0.20 (0.15-0.26) | 9.55 (7.16-12.67) | 0.14 (0.10-0.20) | 0.12 (0.08-0.17) | 5.86 (4.11-8.25) | 0.31 (0.23-0.43) | 0.28 (0.20-0.38) | 13.37 (9.66-18.43) |
| Caribbean | 0.83 (0.69-1.01) | 0.73 (0.60-0.89) | 33.83 (27.90-41.35) | 0.33 (0.24-0.45) | 0.29 (0.21-0.39) | 13.74 (9.91-18.78) | 1.35 (1.11-1.66) | 1.18 (0.97-1.46) | 54.59 (44.49-67.74) |
| Central Latin America | 0.33 (0.29-0.37) | 0.29 (0.25-0.32) | 13.88 (12.28-15.65) | 0.17 (0.14-0.19) | 0.14 (0.12-0.16) | 6.84 (5.71-7.94) | 0.50 (0.44-0.58) | 0.44 (0.38-0.51) | 21.54 (18.73-24.69) |
| Tropical Latin America | 1.26 (1.17-1.36) | 1.11 (1.03-1.19) | 52.12 (48.29-56.09) | 0.40 (0.37-0.44) | 0.34 (0.31-0.37) | 16.11 (14.84-17.46) | 2.17 (1.97-2.36) | 1.93 (1.75-2.09) | 90.21 (82.19-98.02) |
| North Africa and Middle East | 0.41 (0.33-0.50) | 0.35 (0.28-0.43) | 16.84 (13.23-20.53) | 0.39 (0.23-0.52) | 0.33 (0.18-0.44) | 15.94 (8.87-21.24) | 0.42 (0.36-0.51) | 0.37 (0.31-0.44) | 17.63 (14.85-21.12) |
| South Asia | 0.92 (0.79-1.12) | 0.83 (0.71-1.00) | 39.38 (33.90-48.00) | 0.85 (0.69-1.19) | 0.75 (0.60-1.06) | 36.01 (28.95-50.95) | 1.00 (0.85-1.16) | 0.90 (0.77-1.05) | 42.67 (36.36-49.49) |
| East Asia | 2.64 (2.07-3.31) | 1.81 (1.41-2.31) | 85.70 (66.83-108.77) | 0.62 (0.44-0.85) | 0.33 (0.24-0.45) | 15.72 (11.40-21.48) | 4.57 (3.44-5.86) | 3.24 (2.45-4.18) | 152.62 (115.26-196.78) |
| Oceania | 0.40 (0.28-0.56) | 0.36 (0.25-0.51) | 17.38 (12.29-24.77) | 0.21 (0.13-0.36) | 0.19 (0.11-0.33) | 9.43 (5.75-16.35) | 0.58 (0.40-0.87) | 0.52 (0.36-0.79) | 25.23 (17.19-37.90) |
| Southeast Asia | 0.62 (0.50-0.76) | 0.52 (0.42-0.63) | 24.60 (20.08-30.09) | 0.35 (0.26-0.44) | 0.29 (0.21-0.36) | 13.97 (10.36-17.74) | 0.90 (0.70-1.15) | 0.75 (0.59-0.95) | 35.22 (27.64-44.59) |
| Central Sub-Saharan Africa | 1.60 (1.06-2.24) | 1.47 (0.97-2.06) | 68.90 (45.36-96.48) | 1.09 (0.50-1.76) | 1.00 (0.45-1.63) | 47.50 (21.38-77.49) | 2.11 (1.36-3.10) | 1.95 (1.25-2.84) | 90.31 (57.89-131.91) |
| Eastern Sub-Saharan Africa | 2.19 (1.75-2.73) | 2.01 (1.60-2.51) | 94.80 (75.41-118.66) | 1.68 (1.10-2.62) | 1.53 (1.00-2.39) | 73.25 (47.76-114.70) | 2.72 (2.14-3.46) | 2.51 (1.97-3.21) | 116.96 (91.95-150.19) |
| Southern Sub-Saharan Africa | 2.72 (2.31-3.18) | 2.46 (2.09-2.88) | 115.48 (97.92-135.05) | 1.68 (1.34-2.16) | 1.49 (1.18-1.93) | 70.99 (56.10-91.95) | 3.80 (3.18-4.57) | 3.47 (2.90-4.16) | 161.53 (135.10-194.11) |
| Western Sub-Saharan Africa | 0.76 (0.55-0.95) | 0.70 (0.51-0.87) | 32.84 (23.72-40.93) | 0.30 (0.13-0.44) | 0.27 (0.11-0.40) | 12.99 (5.40-18.88) | 1.26 (0.97-1.59) | 1.16 (0.90-1.46) | 54.14 (41.83-68.46) |

Estimates are for individuals aged 20–49 years. Values in parentheses represent the 95% uncertainty intervals (UI). Rates are reported per 100,000 population. UI = Uncertainty Interval, SDI = Socio-demographic Index, DALY = Disability-Adjusted Life Year.

**Supplementary Table 3. Global Burden of Early-Onset Esophageal Cancer by SDI Categories and GBD Regions from 1990 to 2021, Stratified by Sex**

| **Categories** | **Both sexes** | | | | **Women** | | | | **Men** | | | |
| --- | --- | --- | --- | --- | --- | --- | --- | --- | --- | --- | --- | --- |
|  | **Rates in 1990**  **(95% UI)** | **Rates in 2021**  **(95% UI)** | **AAPC, %**  **(95% CI)** | ***P*** | **Rates in 1990**  **(95% UI)** | **Rates in 2021**  **(95% UI)** | **AAPC, %**  **(95% CI)** | ***P*** | **Rates in 1990**  **(95% UI)** | **Rates in 2021**  **(95% UI)** | **AAPC, %**  **(95% CI)** | ***P*** |
| **Age-standardized incidence rate** | | | | | | | | | | | | |
| Global | 2.23  (1.98-2.51) | 1.24  (1.09-1.40) | **-1.90**  **(-2.05--1.75)** | <0.001 | 0.99  (0.66-1.15) | 0.58  (0.49-0.70) | **-1.73**  **(-1.89--1.57)** | <0.001 | 3.42  (2.99-3.94) | 1.88  (1.62-2.18) | **-1.94**  **(-2.12--1.76)** | <0.001 |
| High SDI | 1.13  (1.09-1.18) | 0.85  (0.81-0.90) | **-0.92**  **(-1.13--0.70)** | <0.001 | 0.35  (0.34-0.37) | 0.32  (0.30-0.35) | **-0.31**  **(-0.54--0.07)** | 0.010 | 1.91  (1.82-2.00) | 1.36  (1.28-1.44) | **-1.10**  **(-1.33--0.87)** | <0.001 |
| High-middle SDI | 3.18  (2.69-3.73) | 1.72  (1.38-2.17) | **-2.02**  **(-2.24--1.79)** | <0.001 | 0.95  (0.53-1.21) | 0.40  (0.31-0.52) | **-2.81**  **(-2.99--2.62)** | <0.001 | 5.36  (4.41-6.43) | 3.01  (2.34-3.89) | **-1.89**  **(-2.12--1.66)** | <0.001 |
| Middle SDI | 3.16  (2.63-3.74) | 1.32  (1.11-1.56) | **-2.82**  **(-2.95--2.69)** | <0.001 | 1.34  (0.74-1.67) | 0.55  (0.44-0.67) | **-2.81**  **(-3.07--2.56)** | <0.001 | 4.87  (3.94-5.99) | 2.07  (1.68-2.55) | **-2.74**  **(-2.94--2.53)** | <0.001 |
| Low-middle SDI | 1.12  (0.99-1.29) | 0.94  (0.83-1.11) | **-0.55**  **(-0.61--0.49)** | <0.001 | 1.05  (0.85-1.36) | 0.75  (0.62-1.03) | **-1.04**  **(-1.21--0.86)** | <0.001 | 1.17  (1.03-1.36) | 1.13  (0.99-1.29) | -0.12  (-0.26-0.03) | 0.117 |
| Low SDI | 1.54  (1.26-1.80) | 1.23  (1.02-1.47) | **-0.73**  **(-0.81--0.65)** | <0.001 | 1.41  (0.93-1.71) | 0.98  (0.69-1.29) | **-1.17**  **(-1.28--1.06)** | <0.001 | 1.67  (1.38-2.01) | 1.48  (1.21-1.82) | **-0.39**  **(-0.47--0.32)** | <0.001 |
| Central Asia | 2.69  (2.51-2.87) | 0.89  (0.76-1.03) | **-3.51**  **(-4.01--3.01)** | <0.001 | 2.18  (2.00-2.39) | 0.81  (0.66-0.98) | **-3.06**  **(-3.80--2.32)** | <0.001 | 3.22  (2.97-3.47) | 0.97  (0.84-1.12) | **-3.74**  **(-4.32--3.16)** | <0.001 |
| Central Europe | 0.93  (0.87-0.99) | 0.58  (0.52-0.64) | **-1.52**  **(-1.84--1.20)** | <0.001 | 0.25  (0.23-0.27) | 0.16  (0.15-0.18) | **-1.30**  **(-1.57--1.04)** | <0.001 | 1.62  (1.51-1.74) | 0.98  (0.87-1.09) | **-1.61**  **(-1.95--1.27)** | <0.001 |
| Eastern Europe | 1.18  (1.11-1.25) | 0.83  (0.74-0.93) | **-1.11**  **(-1.85--0.36)** | 0.004 | 0.25  (0.24-0.27) | 0.23  (0.20-0.26) | -0.14  (-1.16-0.90) | 0.794 | 2.18  (2.05-2.32) | 1.48  (1.29-1.68) | **-1.13**  **(-1.75--0.50)** | <0.001 |
| Australasia | 0.59  (0.51-0.68) | 0.66  (0.55-0.79) | 0.31  (-0.12-0.74) | 0.162 | 0.31  (0.25-0.37) | 0.26  (0.21-0.32) | **-0.52**  **(-0.88--0.16)** | 0.005 | 0.86  (0.72-1.03) | 1.07  (0.85-1.33) | **0.67**  **(0.21-1.14)** | 0.004 |
| High-income Asia Pacific | 0.96  (0.89-1.05) | 0.59  (0.54-0.65) | **-1.71**  **(-1.90--1.51)** | <0.001 | 0.30  (0.27-0.34) | 0.33  (0.29-0.36) | 0.45  (-0.48-1.39) | 0.342 | 1.61  (1.47-1.78) | 0.85  (0.76-0.96) | **-2.24**  **(-2.43--2.06)** | <0.001 |
| High-income North America | 0.87  (0.85-0.90) | 0.81  (0.78-0.85) | -0.16  (-0.35-0.04) | 0.125 | 0.33  (0.32-0.34) | 0.34  (0.32-0.36) | 0.03  (-0.30-0.35) | 0.862 | 1.43  (1.38-1.47) | 1.29  (1.23-1.36) | -0.25  (-0.49-0.00) | 0.051 |
| Southern Latin America | 1.18  (1.04-1.36) | 0.53  (0.46-0.62) | **-2.45**  **(-2.83--2.08)** | <0.001 | 0.54  (0.47-0.63) | 0.30  (0.24-0.36) | **-1.89**  **(-2.65--1.12)** | <0.001 | 1.84  (1.58-2.18) | 0.78  (0.64-0.94) | **-2.64**  **(-3.04--2.24)** | <0.001 |
| Western Europe | 1.26  (1.18-1.35) | 0.76  (0.71-0.82) | **-1.66**  **(-1.97--1.34)** | <0.001 | 0.34  (0.33-0.37) | 0.29  (0.27-0.32) | **-0.48**  **(-0.92--0.03)** | 0.036 | 2.17  (2.00-2.34) | 1.23  (1.13-1.33) | **-1.86**  **(-2.20--1.52)** | <0.001 |
| Andean Latin America | 0.37  (0.30-0.44) | 0.23  (0.17-0.30) | **-1.58**  **(-2.45--0.69)** | <0.001 | 0.26  (0.20-0.33) | 0.14  (0.10-0.20) | **-1.83**  **(-3.04--0.60)** | 0.004 | 0.48  (0.37-0.60) | 0.31  (0.23-0.43) | **-1.12**  **(-1.48--0.75)** | <0.001 |
| Caribbean | 0.84  (0.74-0.95) | 0.83  (0.69-1.01) | -0.02  (-0.46-0.43) | 0.947 | 0.46  (0.37-0.56) | 0.33  (0.24-0.45) | **-0.86**  **(-1.54--0.18)** | 0.013 | 1.24  (1.07-1.43) | 1.35  (1.11-1.66) | 0.37  (-0.05-0.78) | 0.084 |
| Central Latin America | 0.49  (0.47-0.52) | 0.33  (0.29-0.37) | **-1.28**  **(-1.69--0.86)** | <0.001 | 0.32  (0.30-0.35) | 0.17  (0.14-0.19) | **-2.11**  **(-2.51--1.71)** | <0.001 | 0.67  (0.62-0.73) | 0.50  (0.44-0.58) | **-0.91**  **(-1.16--0.67)** | <0.001 |
| Tropical Latin America | 1.77  (1.66-1.89) | 1.26  (1.17-1.36) | **-1.08**  **(-1.49--0.67)** | <0.001 | 0.61  (0.56-0.65) | 0.40  (0.37-0.44) | **-1.54**  **(-1.78--1.30)** | <0.001 | 2.97  (2.75-3.21) | 2.17  (1.97-2.36) | **-0.99**  **(-1.42--0.57)** | <0.001 |
| North Africa and Middle East | 0.61  (0.44-0.74) | 0.41  (0.33-0.50) | **-1.29(-1.37--1.21)** | <0.001 | 0.66  (0.34-0.86) | 0.39  (0.23-0.52) | **-1.64**  **(-1.79--1.50)** | <0.001 | 0.57  (0.48-0.68) | 0.42  (0.36-0.51) | **-0.94**  **(-1.06--0.81)** | <0.001 |
| South Asia | 1.14  (1.00-1.38) | 0.92  (0.79-1.12) | **-0.67**  **(-0.77--0.56)** | <0.001 | 1.18  (0.95-1.63) | 0.85  (0.69-1.19) | **-1.03**  **(-1.16--0.89)** | <0.001 | 1.11  (0.95-1.30) | 1.00  (0.85-1.16) | **-0.32**  **(-0.60--0.03)** | 0.030 |
| East Asia | 5.72  (4.70-6.85) | 2.64  (2.07-3.31) | **-2.52**  **(-2.75--2.29)** | <0.001 | 1.87  (0.85-2.47) | 0.62  (0.44-0.85) | **-3.52**  **(-3.90--3.13)** | <0.001 | 9.22  (7.40-11.36) | 4.57  (3.44-5.86) | **-2.29**  **(-2.52--2.06)** | <0.001 |
| Oceania | 0.48  (0.32-0.70) | 0.40  (0.28-0.56) | **-0.62**  **(-0.66--0.58)** | <0.001 | 0.28  (0.15-0.46) | 0.21  (0.13-0.36) | **-0.91**  **(-0.96--0.85)** | <0.001 | 0.67  (0.45-1.04) | 0.58  (0.40-0.87) | **-0.43**  **(-0.56--0.29)** | <0.001 |
| Southeast Asia | 0.71  (0.56-0.87) | 0.62  (0.50-0.76) | **-0.36**  **(-0.51--0.20)** | <0.001 | 0.59  (0.39-0.74) | 0.35  (0.26-0.44) | **-1.69**  **(-1.87--1.50)** | <0.001 | 0.84  (0.67-1.09) | 0.90  (0.70-1.15) | 0.20  (-0.23-0.64) | 0.362 |
| Central Sub-Saharan Africa | 2.28  (1.48-3.20) | 1.60  (1.06-2.24) | **-1.14**  **(-1.25--1.03)** | <0.001 | 1.59  (0.59-2.50) | 1.09  (0.50-1.76) | **-1.22**  **(-1.35--1.09)** | <0.001 | 3.04  (2.06-4.40) | 2.11  (1.36-3.10) | **-1.19**  **(-1.32--1.06)** | <0.001 |
| Eastern Sub-Saharan Africa | 2.90  (2.27-3.49) | 2.19  (1.75-2.73) | **-0.89**  **(-0.99--0.78)** | <0.001 | 2.45  (1.48-3.13) | 1.68  (1.10-2.62) | **-1.19**  **(-1.28--1.11)** | <0.001 | 3.36  (2.68-4.18) | 2.72  (2.14-3.46) | **-0.67**  **(-0.75--0.59)** | <0.001 |
| Southern Sub-Saharan Africa | 4.25  (3.67-4.91) | 2.72  (2.31-3.18) | **-1.51**  **(-1.85--1.18)** | <0.001 | 2.80  (1.70-3.64) | 1.68  (1.34-2.16) | **-1.59**  **(-2.77--0.40)** | 0.009 | 5.78  (4.85-6.94) | 3.80  (3.18-4.57) | **-1.38**  **(-1.92--0.84)** | <0.001 |
| Western Sub-Saharan Africa | 0.50  (0.39-0.63) | 0.76  (0.55-0.95) | **1.39**  **(1.22-1.56)** | <0.001 | 0.32  (0.19-0.42) | 0.30  (0.13-0.44) | **-0.20**  **(-0.37--0.02)** | 0.030 | 0.65  (0.51-0.86) | 1.26  (0.97-1.59) | **2.14**  **(1.99-2.29)** | <0.001 |
| **Age-standardized mortality rate** | | | | | | | | | | | | |
| Global | 1.96  (1.73-2.20) | 0.95  (0.85-1.08) | **-2.33**  **(-2.51--2.14)** | <0.001 | 0.85  (0.57-0.98) | 0.45  (0.38-0.54) | **-2.05**  **(-2.20--1.91)** | <0.001 | 3.02  (2.64-3.49) | 1.45  (1.26-1.68) | **-2.37**  **(-2.55--2.19)** | <0.001 |
| High SDI | 0.87  (0.83-0.91) | 0.54  (0.51-0.57) | **-1.53**  **(-1.77--1.28)** | <0.001 | 0.24  (0.23-0.25) | 0.17  (0.16-0.19) | **-1.10**  **(-1.32--0.89)** | <0.001 | 1.49  (1.42-1.57) | 0.89  (0.83-0.94) | **-1.69**  **(-1.84--1.54)** | <0.001 |
| High-middle SDI | 2.80  (2.37-3.29) | 1.19  (0.96-1.50) | **-2.75**  **(-2.99--2.51)** | <0.001 | 0.80  (0.45-1.02) | 0.25  (0.20-0.31) | **-3.81**  **(-3.96--3.66)** | <0.001 | 4.75  (3.92-5.71) | 2.12  (1.66-2.72) | **-2.62**  **(-2.87--2.38)** | <0.001 |
| Middle SDI | 2.81  (2.33-3.32) | 1.01  (0.86-1.20) | **-3.24**  **(-3.46--3.02)** | <0.001 | 1.12  (0.64-1.39) | 0.39  (0.32-0.46) | **-3.35**  **(-3.65--3.05)** | <0.001 | 4.39  (3.55-5.38) | 1.63  (1.33-1.97) | **-3.16**  **(-3.38--2.94)** | <0.001 |
| Low-middle SDI | 1.03  (0.91-1.19) | 0.85  (0.75-1.00) | **-0.63**  **(-0.75--0.52)** | <0.001 | 0.96  (0.77-1.25) | 0.67  (0.55-0.91) | **-1.12**  **(-1.30--0.95)** | <0.001 | 1.09  (0.95-1.26) | 1.02  (0.90-1.17) | **-0.18**  **(-0.32--0.03)** | 0.020 |
| Low SDI | 1.43  (1.16-1.66) | 1.13  (0.93-1.36) | **-0.76**  **(-0.84--0.68)** | <0.001 | 1.30  (0.86-1.58) | 0.89  (0.62-1.17) | **-1.21**  **(-1.33--1.10)** | <0.001 | 1.56  (1.29-1.88) | 1.37  (1.12-1.67) | **-0.42**  **(-0.50--0.34)** | <0.001 |
| Central Asia | 2.48  (2.31-2.64) | 0.81  (0.69-0.93) | **-3.55**  **(-4.05--3.05)** | <0.001 | 2.00  (1.83-2.19) | 0.73  (0.60-0.88) | **-3.13**  **(-3.86--2.40)** | <0.001 | 2.97  (2.75-3.21) | 0.89  (0.77-1.02) | **-3.77**  **(-4.35--3.19)** | <0.001 |
| Central Europe | 0.85  (0.79-0.90) | 0.50  (0.45-0.56) | **-1.65**  **(-1.97--1.34)** | <0.001 | 0.22  (0.21-0.24) | 0.13  (0.12-0.15) | **-1.72**  **(-2.12--1.31)** | <0.001 | 1.47  (1.38-1.59) | 0.86  (0.77-0.96) | **-1.72**  **(-2.06--1.38)** | <0.001 |
| Eastern Europe | 1.02  (0.97-1.08) | 0.67  (0.60-0.75) | **-1.31**  **(-1.89--0.74)** | <0.001 | 0.22  (0.21-0.23) | 0.18  (0.16-0.20) | -0.46  (-1.50-0.59) | 0.386 | 1.90  (1.79-2.03) | 1.19  (1.04-1.35) | **-1.39**  **(-2.00--0.78)** | <0.001 |
| Australasia | 0.46  (0.40-0.53) | 0.46  (0.38-0.56) | -0.04  (-0.48-0.39) | 0.839 | 0.21  (0.17-0.25) | 0.15  (0.12-0.18) | **-1.17**  **(-1.45--0.90)** | <0.001 | 0.70  (0.59-0.84) | 0.78  (0.63-0.97) | 0.31  (-0.10-0.73) | 0.141 |
| High-income Asia Pacific | 0.63  (0.58-0.70) | 0.25  (0.23-0.27) | **-3.05**  **(-3.39--2.71)** | <0.001 | 0.17  (0.15-0.19) | 0.13  (0.12-0.14) | **-0.87**  **(-1.14--0.59)** | <0.001 | 1.09  (0.99-1.21) | 0.36  (0.33-0.40) | **-3.61**  **(-3.98--3.24)** | <0.001 |
| High-income North America | 0.63  (0.62-0.65) | 0.52  (0.50-0.55) | -0.51  (-1.04-0.02) | 0.061 | 0.20  (0.19-0.20) | 0.17  (0.16-0.18) | **-0.55**  **(-0.90--0.19)** | 0.002 | 1.08  (1.05-1.12) | 0.89  (0.84-0.93) | **-0.58**  **(-0.82--0.34)** | <0.001 |
| Southern Latin America | 1.05  (0.93-1.21) | 0.44  (0.37-0.51) | **-2.66**  **(-3.09--2.23)** | <0.001 | 0.46  (0.40-0.54) | 0.23  (0.19-0.27) | **-2.22**  **(-2.79--1.64)** | <0.001 | 1.66  (1.42-1.97) | 0.66  (0.54-0.80) | **-2.85**  **(-3.25--2.44)** | <0.001 |
| Western Europe | 1.00  (0.93-1.07) | 0.48  (0.45-0.51) | **-2.42**  **(-2.71--2.12)** | <0.001 | 0.25  (0.24-0.27) | 0.16  (0.15-0.17) | **-1.41**  **(-1.74--1.07)** | <0.001 | 1.74  (1.61-1.88) | 0.79  (0.73-0.85) | **-2.57**  **(-2.89--2.25)** | <0.001 |
| Andean Latin America | 0.33  (0.28-0.40) | 0.20  (0.15-0.26) | **-1.75**  **(-2.61--0.89)** | <0.001 | 0.23  (0.18-0.30) | 0.12  (0.08-0.17) | **-2.07**  **(-3.26--0.87)** | 0.001 | 0.44  (0.34-0.56) | 0.28  (0.20-0.38) | **-1.42**  **(-2.28--0.56)** | 0.001 |
| Caribbean | 0.75  (0.66-0.85) | 0.73  (0.60-0.89) | -0.09  (-0.54-0.36) | 0.685 | 0.40  (0.32-0.49) | 0.29  (0.21-0.39) | **-0.91**  **(-1.56--0.25)** | 0.007 | 1.12  (0.96-1.29) | 1.18  (0.97-1.46) | 0.28  (-0.14-0.69) | 0.191 |
| Central Latin America | 0.45  (0.42-0.47) | 0.29  (0.25-0.32) | **-1.42**  **(-1.79--1.06)** | <0.001 | 0.29  (0.27-0.31) | 0.14  (0.12-0.16) | **-2.30**  **(-2.65--1.95)** | <0.001 | 0.61  (0.57-0.66) | 0.44  (0.38-0.51) | **-1.04**  **(-1.28--0.79)** | <0.001 |
| Tropical Latin America | 1.61  (1.51-1.72) | 1.11  (1.03-1.19) | **-1.19**  **(-1.60--0.78)** | <0.001 | 0.54  (0.50-0.58) | 0.34  (0.31-0.37) | **-1.68**  **(-1.90--1.45)** | <0.001 | 2.72  (2.52-2.94) | 1.93  (1.75-2.09) | **-1.09**  **(-1.52--0.67)** | <0.001 |
| North Africa and Middle East | 0.56  (0.40-0.67) | 0.35  (0.28-0.43) | **-1.48**  **(-1.57--1.40)** | <0.001 | 0.59  (0.30-0.78) | 0.33  (0.18-0.44) | **-1.85**  **(-1.99--1.71)** | <0.001 | 0.52  (0.43-0.63) | 0.37  (0.31-0.44) | **-1.11**  **(-1.24--0.99)** | <0.001 |
| South Asia | 1.05  (0.92-1.27) | 0.83  (0.71-1.00) | **-0.75**  **(-0.86--0.65)** | <0.001 | 1.08  (0.87-1.50) | 0.75  (0.60-1.06) | **-1.13**  **(-1.27--1.00)** | <0.001 | 1.03  (0.88-1.21) | 0.90  (0.77-1.05) | **-0.39**  **(-0.67--0.11)** | 0.007 |
| East Asia | 5.05  (4.16-6.05) | 1.81  (1.41-2.31) | **-3.35**  **(-3.65--3.06)** | <0.001 | 1.54  (0.71-2.03) | 0.33  (0.24-0.45) | **-4.97**  **(-5.51--4.43)** | <0.001 | 8.23  (6.62-10.15) | 3.24  (2.45-4.18) | **-3.01**  **(-3.42--2.60)** | <0.001 |
| Oceania | 0.43  (0.29-0.64) | 0.36  (0.25-0.51) | **-0.66**  **(-0.70--0.62)** | <0.001 | 0.25  (0.13-0.43) | 0.19  (0.11-0.33) | **-0.95**  **(-1.01--0.89)** | <0.001 | 0.60  (0.40-0.94) | 0.52  (0.36-0.79) | **-0.45**  **(-0.67--0.23)** | <0.001 |
| Southeast Asia | 0.64  (0.51-0.78) | 0.52  (0.42-0.63) | **-0.64**  **(-0.82--0.45)** | <0.001 | 0.53  (0.35-0.66) | 0.29  (0.21-0.36) | **-1.95**  **(-2.12--1.78)** | <0.001 | 0.76  (0.61-0.98) | 0.75  (0.59-0.95) | -0.08  (-0.50-0.35) | 0.726 |
| Central Sub-Saharan Africa | 2.12  (1.38-2.99) | 1.47  (0.97-2.06) | **-1.17**  **(-1.28--1.06)** | <0.001 | 1.47  (0.52-2.34) | 1.00  (0.45-1.63) | **-1.27**  **(-1.40--1.13)** | <0.001 | 2.84  (1.92-4.12) | 1.95  (1.25-2.84) | **-1.22**  **(-1.35--1.09)** | <0.001 |
| Eastern Sub-Saharan Africa | 2.69  (2.10-3.24) | 2.01  (1.60-2.51) | **-0.92**  **(-1.03--0.82)** | <0.001 | 2.25  (1.35-2.88) | 1.53  (1.00-2.39) | **-1.24**  **(-1.33--1.15)** | <0.001 | 3.13  (2.51-3.88) | 2.51  (1.97-3.21) | **-0.70**  **(-0.78--0.62)** | <0.001 |
| Southern Sub-Saharan Africa | 3.86  (3.34-4.47) | 2.46  (2.09-2.88) | **-1.54**  **(-1.87--1.22)** | <0.001 | 2.50  (1.53-3.24) | 1.49  (1.18-1.93) | **-1.62**  **(-2.78--0.43)** | 0.007 | 5.30  (4.45-6.37) | 3.47  (2.90-4.16) | **-1.40**  **(-1.93--0.86)** | <0.001 |
| Western Sub-Saharan Africa | 0.46  (0.36-0.58) | 0.70  (0.51-0.87) | **1.36**  **(1.20-1.53)** | <0.001 | 0.29  (0.18-0.39) | 0.27  (0.11-0.40) | **-0.23**  **(-0.41--0.05)** | 0.014 | 0.61  (0.47-0.80) | 1.16  (0.90-1.46) | **2.11**  **(1.96-2.25)** | <0.001 |
| **Age-standardized DALYs rate** | | | | | | | | | | | | |
| Global | 92.16(81.70-103.77) | 45.07(40.03-50.89) | **-2.31**  **(-2.49--2.14)** | <0.001 | 40.42(27.12-46.69) | 21.58(18.26-26.29) | **-2.01**  **(-2.16--1.87)** | <0.001 | 141.95(123.82-163.75) | 68.24(59.12-78.56) | **-2.36**  **(-2.54--2.18)** | <0.001 |
| High SDI | 40.31  (38.58-42.18) | 25.35  (24.06-26.73) | **-1.48**  **(-1.65--1.30)** | <0.001 | 11.22  (10.71-11.78) | 8.09  (7.61-8.96) | **-1.07**  **(-1.29--0.84)** | <0.001 | 68.98  (65.64-72.63) | 41.74  (39.37-44.35) | **-1.60**  **(-1.79--1.42)** | <0.001 |
| High-middle SDI | 131.25  (111.23-154.42) | 56.10  (45.03-70.42) | **-2.81**  **(-3.02--2.60)** | <0.001 | 38.11  (21.45-48.29) | 11.81  (9.52-14.73) | **-3.76**  **(-3.91--3.62)** | <0.001 | 222.24  (183.34-267.90) | 99.32  (77.88-127.33) | **-2.62**  **(-2.85--2.39)** | <0.001 |
| Middle SDI | 132.50  (110.07-156.35) | 47.96  (40.87-56.37) | **-3.26**  **(-3.42--3.10)** | <0.001 | 53.36  (30.60-66.01) | 18.97  (15.36-22.28) | **-3.30**  **(-3.59--3.01)** | <0.001 | 206.59  (167.26-253.18) | 76.64  (62.79-92.59) | **-3.17**  **(-3.41--2.93)** | <0.001 |
| Low-middle SDI | 48.85  (43.11-56.53) | 40.22  (35.48-47.85) | **-0.64**  **(-0.76--0.52)** | <0.001 | 46.34  (37.02-59.97) | 32.20  (26.28-44.29) | **-1.12**  **(-1.26--0.99)** | <0.001 | 51.17  (44.76-59.20) | 48.27  (42.48-54.98) | **-0.17**  **(-0.32--0.03)** | 0.018 |
| Low SDI | 67.42  (54.72-78.56) | 53.30  (43.98-64.15) | **-0.76**  **(-0.85--0.67)** | <0.001 | 61.96  (40.77-75.62) | 42.64  (29.91-56.40) | **-1.22**  **(-1.28--1.15)** | <0.001 | 72.64  (60.06-87.71) | 64.01  (52.19-78.34) | **-0.41**  **(-0.49--0.33)** | <0.001 |
| Central Asia | 117.52  (109.60-125.34) | 38.74  (33.26-44.98) | **-3.52**  **(-4.02--3.02)** | <0.001 | 96.48  (88.18-105.42) | 35.15  (28.83-42.68) | **-3.12**  **(-3.86--2.37)** | <0.001 | 139.33  (128.65-150.47) | 42.52  (36.72-48.91) | **-3.71**  **(-4.28--3.13)** | <0.001 |
| Central Europe | 39.08  (36.70-41.80) | 23.29  (21.03-25.74) | **-1.66**  **(-1.98--1.34)** | <0.001 | 10.57  (9.88-11.35) | 6.39  (5.73-7.13) | **-1.72**  **(-2.12--1.32)** | <0.001 | 67.89  (63.32-73.09) | 39.69  (35.36-44.26) | **-1.73**  **(-2.07--1.39)** | <0.001 |
| Eastern Europe | 46.70  (44.31-49.42) | 30.98  (27.72-34.64) | **-1.26**  **(-1.84--0.67)** | <0.001 | 10.36  (9.85-10.92) | 8.54  (7.48-9.62) | -0.47  (-1.48-0.54) | 0.362 | 86.15  (81.17-91.80) | 54.99  (48.27-62.57) | **-1.33**  **(-1.95--0.70)** | <0.001 |
| Australasia | 21.62  (18.63-25.15) | 21.67  (17.98-26.18) | -0.06  (-0.49-0.38) | 0.798 | 9.99  (8.31-11.98) | 7.01  (5.67-8.67) | **-1.20**  **(-1.47--0.94)** | <0.001 | 32.98  (27.44-39.58) | 36.74  (29.38-45.69) | 0.30  (-0.18-0.78) | 0.217 |
| High-income Asia Pacific | 29.08  (26.63-32.19) | 11.62  (10.82-12.65) | **-3.01**  **(-3.36--2.67)** | <0.001 | 8.20  (7.13-9.17) | 6.10  (5.70-6.59) | **-0.84**  **(-1.11--0.57)** | <0.001 | 49.80  (45.09-55.59) | 16.91  (15.46-18.93) | **-3.44**  **(-3.92--2.95)** | <0.001 |
| High-income North America | 29.68  (28.87-30.48) | 25.08  (24.03-26.17) | -0.44  (-0.98-0.11) | 0.118 | 9.30  (8.97-9.65) | 8.30  (7.89-8.78) | **-0.48**  **(-0.85--0.11)** | 0.010 | 50.57  (49.02-52.15) | 42.19  (40.09-44.30) | **-0.50**  **(-0.76--0.25)** | <0.001 |
| Southern Latin America | 49.35  (43.40-56.61) | 20.82  (17.76-24.35) | **-2.62**  **(-3.03--2.20)** | <0.001 | 22.22  (18.95-25.99) | 10.92  (8.98-13.16) | **-2.20**  **(-2.97--1.43)** | <0.001 | 77.49  (65.87-91.68) | 31.12  (25.37-37.84) | **-2.80**  **(-3.19--2.40)** | <0.001 |
| Western Europe | 46.45  (43.30-49.73) | 22.46  (21.14-23.95) | **-2.36**  **(-2.66--2.06)** | <0.001 | 11.86  (11.27-12.55) | 7.72  (7.26-8.23) | **-1.38**  **(-1.71--1.05)** | <0.001 | 80.59  (74.34-87.05) | 37.21  (34.66-40.12) | **-2.52**  **(-2.84--2.19)** | <0.001 |
| Andean Latin America | 16.21  (13.44-19.55) | 9.55  (7.16-12.67) | **-1.75**  **(-2.61--0.89)** | <0.001 | 11.29  (8.71-14.42) | 5.86  (4.11-8.25) | **-2.09**  **(-3.29--0.88)** | 0.001 | 21.25  (16.33-26.93) | 13.37  (9.66-18.43) | **-1.41**  **(-2.28--0.53)** | 0.002 |
| Caribbean | 35.31  (30.90-40.15) | 33.83  (27.90-41.35) | -0.12  (-0.56-0.32) | 0.601 | 19.14  (15.19-23.60) | 13.74  (9.91-18.78) | -0.99  (-1.75--0.24) | 0.010 | 52.13  (44.68-60.38) | 54.59  (44.49-67.74) | 0.24  (-0.16-0.65) | 0.237 |
| Central Latin America | 21.74  (20.58-23.01) | 13.88  (12.28-15.65) | **-1.42**  **(-1.77--1.06)** | <0.001 | 14.01  (13.13-15.10) | 6.84  (5.71-7.94) | **-2.29**  **(-2.64--1.94)** | <0.001 | 29.81  (27.66-32.28) | 21.54  (18.73-24.69) | **-1.08**  **(-1.50--0.66)** | <0.001 |
| Tropical Latin America | 75.42  (70.67-80.49) | 52.12  (48.29-56.09) | **-1.18**  **(-1.58--0.78)** | <0.001 | 25.65  (23.79-27.58) | 16.11  (14.84-17.46) | **-1.68**  **(-1.91--1.46)** | <0.001 | 126.80  (117.38-136.84) | 90.21  (82.19-98.02) | **-1.09**  **(-1.50--0.67)** | <0.001 |
| North Africa and Middle East | 26.55  (19.08-32.09) | 16.84  (13.23-20.53) | **-1.46**  **(-1.55--1.38)** | <0.001 | 28.39  (14.47-37.31) | 15.94  (8.87-21.24) | **-1.83**  **(-1.99--1.68)** | <0.001 | 24.79  (20.63-29.81) | 17.63  (14.85-21.12) | **-1.08**  **(-1.20--0.96)** | <0.001 |
| South Asia | 50.08  (43.77-60.75) | 39.38  (33.90-48.00) | **-0.75**  **(-0.87--0.64)** | <0.001 | 51.75  (41.71-71.93) | 36.01  (28.95-50.95) | **-1.14**  **(-1.27--1.01)** | <0.001 | 48.49  (41.75-57.00) | 42.67  (36.36-49.49) | **-0.38**  **(-0.68--0.08)** | 0.013 |
| East Asia | 237.02  (195.53-283.99) | 85.70  (66.83-108.77) | **-3.31**  **(-3.65--2.97)** | <0.001 | 72.43  (33.65-95.44) | 15.72  (11.40-21.48) | **-4.84**  **(-5.14--4.54)** | <0.001 | 386.88  (311.53-476.03) | 152.62  (115.26-196.78) | **-3.06**  **(-3.35--2.76)** | <0.001 |
| Oceania | 21.03  (14.00-30.88) | 17.38  (12.29-24.77) | **-0.65**  **(-0.69--0.60)** | <0.001 | 12.46  (6.60-21.34) | 9.43  (5.75-16.35) | **-0.92**  **(-0.98--0.86)** | <0.001 | 28.98  (19.29-44.96) | 25.23  (17.19-37.90) | **-0.42**  **(-0.66--0.18)** | 0.001 |
| Southeast Asia | 30.75  (24.25-37.34) | 24.60  (20.08-30.09) | **-0.67**  **(-0.84--0.49)** | <0.001 | 25.80  (16.83-32.41) | 13.97  (10.36-17.74) | **-1.96**  **(-2.12--1.79)** | <0.001 | 35.88  (28.66-46.18) | 35.22  (27.64-44.59) | 0.02  (-0.25-0.29) | 0.874 |
| Central Sub-Saharan Africa | 98.89  (63.99-139.54) | 68.90  (45.36-96.48) | **-1.17**  **(-1.27--1.06)** | <0.001 | 69.99  (24.72-111.61) | 47.50  (21.38-77.49) | **-1.26**  **(-1.40--1.13)** | <0.001 | 131.02  (88.63-190.07) | 90.31  (57.89-131.91) | **-1.21**  **(-1.33--1.08)** | <0.001 |
| Eastern Sub-Saharan Africa | 126.21  (98.57-152.07) | 94.80  (75.41-118.66) | **-0.91**  **(-1.00--0.82)** | <0.001 | 107.69  (64.53-137.71) | 73.25  (47.76-114.70) | **-1.24**  **(-1.32--1.15)** | <0.001 | 144.97  (116.13-180.17) | 116.96  (91.95-150.19) | **-0.68**  **(-0.76--0.60)** | <0.001 |
| Southern Sub-Saharan Africa | 183.48  (158.09-212.92) | 115.48  (97.92-135.05) | **-1.67**  **(-2.07--1.26)** | <0.001 | 121.75  (73.98-157.81) | 70.99  (56.10-91.95) | **-1.70**  **(-2.85--0.55)** | 0.004 | 248.83  (209.26-298.70) | 161.53  (135.10-194.11) | **-1.43**  **(-1.97--0.88)** | <0.001 |
| Western Sub-Saharan Africa | 21.51  (16.66-27.17) | 32.84(23.72-40.93) | **1.38**  **(1.21-1.55)** | <0.001 | 13.92(8.31-18.31) | 12.99  (5.40-18.88) | **-0.23**  **(-0.40--0.05)** | 0.011 | 28.12  (21.85-37.23) | 54.14  (41.83-68.46) | **2.13**  **(1.99-2.28)** | <0.001 |

Estimates are for individuals aged 20–49 years. Values in parentheses are 95% uncertainty intervals (UI) or confidence intervals (CI). Rates are reported per 100000 population. Bold within the table means the average annual percent change (AAPC) is statistically significant (*P*≤0.05). UI=uncertainty interval. CI=confidence interval. AAPC=average annual percent change. SDI=Socio-demographic Index. GBD=Global Burden of Disease. DALY=disability-adjusted life year. *P*=*P* value for the significant test of AAPC.

**Supplementary Table 4. Early-Onset Esophageal Cancer Burden by Different Countries and Territories from 1990 to 2021**

| **Categories** | **Age-standardized incidence rate** | | | | **Age-standardized mortality rate** | | | | **Age-standardized DALYs rate** | | | |
| --- | --- | --- | --- | --- | --- | --- | --- | --- | --- | --- | --- | --- |
|  | **Rates in 1990**  **(95% UI)** | **Rates in 2021 (95% UI)** | **AAPC, %**  **(95% CI)** | ***P*** | **Rates in 1990**  **(95% UI)** | **Rates in 2021 (95% UI)** | **AAPC, %**  **(95% CI)** | ***P*** | **Rates in 1990**  **(95% UI)** | **Rates in 2021 (95% UI)** | **AAPC, %**  **(95% CI)** | ***P*** |
| Afghanistan | 2.57  (4.37-0.93) | 1.83  (3.17-0.77) | **-1.07**  **(-1.27--0.87)** | <0.001 | 2.37  (4.05-0.87) | 1.68  (2.91-0.70) | **-1.09**  **(-1.29--0.89)** | <0.001 | 112.98  (193.68-40.94) | 79.98  (138.48-33.31) | **-1.04**  **(-1.15--0.93)** | <0.001 |
| Albania | 0.37  (0.49-0.27) | 0.32  (0.46-0.21) | -0.66  (-1.72-0.40) | 0.220 | 0.34  (0.45-0.25) | 0.28  (0.41-0.18) | -0.79  (-1.82-0.26) | 0.14 | 16.06  (21.43-11.93) | 13.11  (19.40-8.61) | -0.82  (-1.85-0.21) | 0.119 |
| Algeria | 0.15  (0.20-0.10) | 0.12  (0.18-0.08) | **-0.49**  **(-0.61--0.37)** | <0.001 | 0.13  (0.18-0.09) | 0.10  (0.15-0.07) | **-0.71**  **(-0.82--0.60)** | <0.001 | 6.23  (8.58-4.37) | 4.96  (7.01-3.32) | **-0.70**  **(-0.82--0.58)** | <0.001 |
| American Samoa | 0.28  (0.43-0.18) | 0.40  (0.61-0.24) | **1.12**  **(0.18-2.07)** | 0.019 | 0.25  (0.39-0.16) | 0.35  (0.53-0.21) | **1.07**  **(0.30-1.84)** | 0.006 | 12.20  (18.89-7.74) | 16.90  (25.72-10.05) | **1.09**  **(0.31-1.87)** | 0.006 |
| Andorra | 0.47  (0.75-0.28) | 0.32  (0.52-0.17) | **-1.41**  **(-1.75--1.07)** | <0.001 | 0.37  (0.59-0.22) | 0.20  (0.34-0.11) | **-2.06**  **(-2.56--1.55)** | <0.001 | 17.05  (27.47-10.32) | 9.56  (15.71-5.37) | **-2.04**  **(-2.51--1.56)** | <0.001 |
| Angola | 2.58  (3.88-1.52) | 1.56  (2.41-0.92) | **-1.63**  **(-2.25--1.01)** | <0.001 | 2.40  (3.63-1.40) | 1.43  (2.20-0.84) | **-1.68**  **(-2.29--1.06)** | <0.001 | 111.88  (169.42-65.08) | 67.15  (103.70-39.30) | **-1.65**  **(-2.26--1.04)** | <0.001 |
| Antigua and Barbuda | 0.62  (0.71-0.54) | 0.40  (0.46-0.34) | **-0.56**  **(-1.11--0.01)** | 0.046 | 0.55  (0.63-0.48) | 0.34  (0.39-0.29) | **-0.67**  **(-1.22--0.11)** | 0.019 | 25.95  (29.71-22.61) | 15.82  (18.33-13.47) | **-0.67**  **(-1.20--0.13)** | 0.014 |
| Argentina | 1.25  (1.49-1.05) | 0.56  (0.68-0.45) | **-2.48**  **(-2.99--1.97)** | <0.001 | 1.12  (1.34-0.93) | 0.47  (0.57-0.38) | **-2.68**  **(-3.18--2.18)** | <0.001 | 52.17  (62.41-43.70) | 22.12  (27.09-17.97) | **-2.64**  **(-3.12--2.15)** | <0.001 |
| Armenia | 0.51  (0.54-0.47) | 0.12  (0.14-0.11) | **-4.34**  **(-5.70--2.97)** | <0.001 | 0.46  (0.49-0.43) | 0.11  (0.12-0.09) | **-4.41**  **(-5.78--3.02)** | <0.001 | 21.83  (23.19-20.41) | 5.05  (5.74-4.42) | **-4.46**  **(-5.78--3.11)** | <0.001 |
| Australia | 0.58  (0.69-0.49) | 0.68  (0.84-0.55) | **0.47**  **(0.01-0.93)** | 0.047 | 0.46  (0.55-0.39) | 0.49  (0.60-0.40) | 0.15  (-0.32-0.62) | 0.536 | 21.82  (25.85-18.25) | 22.87  (28.34-18.57) | 0.13  (-0.33-0.60) | 0.577 |
| Austria | 0.77  (0.95-0.63) | 0.46  (0.58-0.37) | **-1.45**  **(-1.84--1.07)** | <0.001 | 0.61  (0.76-0.49) | 0.30  (0.38-0.24) | **-2.12**  **(-2.55--1.69)** | <0.001 | 28.27  (35.03-22.78) | 14.26  (17.81-11.29) | **-2.10**  **(-2.52--1.68)** | <0.001 |
| Azerbaijan | 1.90  (2.28-1.56) | 0.79  (1.02-0.59) | **-2.90**  **(-3.40--2.39)** | <0.001 | 1.75  (2.11-1.44) | 0.72  (0.92-0.54) | **-2.95**  **(-3.45--2.44)** | <0.001 | 82.92  (99.77-68.30) | 34.12  (43.73-25.51) | **-2.73**  **(-3.10--2.36)** | <0.001 |
| Bahamas | 1.67  (1.96-1.42) | 1.60  (2.11-1.20) | 0.02  (-1.31-1.37) | 0.976 | 1.51  (1.77-1.28) | 1.42  (1.89-1.06) | -0.06  (-1.35-1.24) | 0.922 | 70.60  (82.50-59.95) | 66.90  (88.63-50.31) | -0.02  (-1.35-1.32) | 0.974 |
| Bahrain | 0.42  (0.59-0.30) | 0.25  (0.37-0.17) | **-1.57**  **(-1.92--1.23)** | <0.001 | 0.38  (0.53-0.27) | 0.20  (0.30-0.13) | **-1.92**  **(-2.26--1.57)** | <0.001 | 17.98  (25.23-12.72) | 9.85  (14.66-6.53) | **-1.83**  **(-2.12--1.52)** | <0.001 |
| Bangladesh | 1.34  (1.98-0.89) | 0.78  (1.25-0.47) | **-1.66**  **(-1.92--1.41)** | <0.001 | 1.24  (1.83-0.82) | 0.70  (1.13-0.42) | **-1.78**  **(-2.03--1.52)** | <0.001 | 58.60  (86.67-38.83) | 33.28  (54.48-19.91) | **-1.74**  **(-2.00--1.49)** | <0.001 |
| Barbados | 1.33  (1.53-1.14) | 1.04  (1.39-0.75) | -0.49  (-1.37-0.41) | 0.285 | 1.19  (1.38-1.03) | 0.89  (1.19-0.65) | -0.87  (-2.09-0.36) | 0.166 | 55.59  (64.11-48.01) | 41.81  55.66-30.73) | -0.61  (-1.49-0.27) | 0.173 |
| Belarus | 0.83  (1.02-0.67) | 0.76  (1.02-0.55) | -0.27  (-1.46-0.93) | 0.659 | 0.74  (0.91-0.60) | 0.61  (0.82-0.45) | -0.57  (-1.73-0.61) | 0.346 | 33.97  (41.70-27.40) | 28.04  (37.56-20.36) | -0.58  (-1.76-0.63) | 0.346 |
| Belgium | 1.18  (1.42-0.97) | 0.87  (1.07-0.70) | **-1.09**  **(-1.60--0.57)** | <0.001 | 0.95  (1.14-0.78) | 0.57  (0.71-0.46) | **-1.73**  **(-2.24--1.22)** | <0.001 | 44.23  (52.95-36.28) | 26.90  (33.13-21.50) | **-1.71**  **(-2.22--1.21)** | <0.001 |
| Belize | 0.39  (0.45-0.34) | 0.55  (0.67-0.46) | **1.19**  **(0.45-1.94)** | 0.002 | 0.36  (0.40-0.31) | 0.49  (0.59-0.40) | **1.13**  **(0.40-1.86)** | 0.002 | 16.63  (18.84-14.55) | 23.12  (27.71-19.00) | **1.16**  **(0.37-1.95)** | 0.004 |
| Benin | 0.57  (0.81-0.39) | 0.92  (1.39-0.54) | **1.52**  **(1.38-1.66)** | <0.001 | 0.53  (0.75-0.36) | 0.84  (1.29-0.50) | **1.50**  **(1.36-1.64)** | <0.001 | 24.78  (34.89-16.86) | 39.50  (60.32-23.44) | **1.52**  **(1.38-1.65)** | <0.001 |
| Bermuda | 1.36  (1.73-1.07) | 0.90  (1.21-0.65) | **-1.34**  **(-1.64--1.04)** | <0.001 | 1.19  (1.51-0.93) | 0.68  (0.92-0.49) | **-1.81**  **(-2.09--1.53)** | <0.001 | 55.19  (69.97-42.98) | 31.59  (42.95-22.72) | **-1.79**  **(-2.08--1.50)** | <0.001 |
| Bhutan | 1.31  (1.95-0.83) | 0.81  (1.25-0.50) | **-1.55**  **(-1.70--1.40)** | <0.001 | 1.21  (1.80-0.78) | 0.73  (1.13-0.45) | **-1.64**  **(-1.79--1.50)** | <0.001 | 57.26  (85.08-36.81) | 34.56  (54.01-21.18) | **-1.62**  **(-1.77--1.47)** | <0.001 |
| Bolivia (Plurinational State of) | 0.49  (0.71-0.34) | 0.31  (0.47-0.19) | **-1.51**  **(-1.66--1.35)** | <0.001 | 0.46  (0.66-0.31) | 0.28  (0.43-0.17) | **-1.58**  **(-1.75--1.42)** | <0.001 | 21.83  (31.77-14.76) | 13.39  (20.63-8.23) | **-1.56**  **(-1.72--1.41)** | <0.001 |
| Bosnia and Herzegovina | 0.56  (0.69-0.46) | 0.42  (0.56-0.27) | **-1.04**  **(-1.79--0.29)** | 0.006 | 0.52  (0.64-0.42) | 0.37  (0.50-0.24) | **-1.14**  **(-1.92--0.36)** | 0.004 | 23.99  (29.58-19.64) | 17.19  (23.28-11.25) | **-1.15**  **(-1.91--0.38)** | 0.003 |
| Botswana | 2.90  (4.64-1.71) | 1.73  (2.63-1.04) | **-1.67**  **(-2.23--1.10)** | <0.001 | 2.67  (4.31-1.57) | 1.57  (2.36-0.97) | **-1.70**  **(-2.25--1.14)** | <0.001 | 123.96  (200.28-72.93) | 73.25  (110.05-44.78) | **-1.69**  **(-2.25--1.13)** | <0.001 |
| Brazil | 1.80  (1.92-1.68) | 1.27  (1.37-1.18) | **-1.10**  **(-1.51--0.68)** | <0.001 | 1.64  (1.75-1.53) | 1.12  (1.21-1.04) | **-1.20**  **(-1.62--0.79)** | <0.001 | 76.53  (81.72-71.69) | 52.65  (56.69-48.69) | **-1.20**  **(-1.60--0.79)** | <0.001 |
| Brunei Darussalam | 0.39  (0.54-0.27) | 0.37  (0.51-0.26) | -0.15  (-0.62-0.33) | 0.546 | 0.33  (0.46-0.23) | 0.27  (0.38-0.19) | **-0.51**  **(-0.98--0.04)** | 0.035 | 15.45  (21.61-10.71) | 12.68  (17.79-8.97) | **-0.57**  **(-1.01--0.12)** | 0.013 |
| Bulgaria | 0.77  (0.87-0.68) | 0.65  (0.79-0.53) | -0.49  (-1.01-0.02) | 0.06 | 0.69  (0.78-0.61) | 0.56  (0.68-0.45) | **-0.62**  **(-1.13--0.11)** | 0.018 | 32.31  (36.41-28.57) | 26.13  (31.56-21.19) | **-0.63**  **(-1.13--0.12)** | 0.015 |
| Burkina Faso | 0.58  (0.84-0.38) | 0.99  (1.55-0.59) | **1.75**  **(1.54-1.96)** | <0.001 | 0.54  (0.78-0.35) | 0.92  (1.43-0.54) | **1.74**  **(1.53-1.96)** | <0.001 | 25.15  (36.27-16.47) | 42.83  (66.75-25.11) | **1.74**  **(1.52-1.96)** | <0.001 |
| Burundi | 4.41  (6.60-2.67) | 2.11  (3.16-1.35) | **-2.38**  **(-2.55--2.22)** | <0.001 | 4.09  (6.20-2.49) | 1.95  (2.97-1.25) | **-2.40**  **(-2.56--2.24)** | <0.001 | 192.10  (291.60-116.38) | 92.12  (140.51-58.64) | **-2.38**  **(-2.55--2.22)** | <0.001 |
| Cabo Verde | 1.94  (2.76-1.34) | 3.18  (4.96-1.92) | **1.63**  **(1.27-1.99)** | <0.001 | 1.77  (2.52-1.23) | 2.85  (4.42-1.75) | **1.57**  **(1.20-1.94)** | <0.001 | 84.71  (121.03-58.39) | 133.00  (206.10-81.51) | **1.48**  **(0.92-2.05)** | <0.001 |
| Cambodia | 1.13  (1.60-0.74) | 0.70  (1.06-0.44) | **-1.52**  **(-1.64--1.40)** | <0.001 | 1.04  (1.47-0.68) | 0.62  (0.94-0.40) | **-1.65**  **(-1.77--1.53)** | <0.001 | 50.06  (70.98-32.34) | 29.78  (45.31-19.00) | **-1.65**  **(-1.76--1.54)** | <0.001 |
| Cameroon | 0.69  (1.01-0.46) | 1.31  (2.13-0.74) | **2.10**  **(1.97-2.23)** | <0.001 | 0.64  (0.93-0.42) | 1.21  (2.00-0.68) | **2.09**  **(1.98-2.20)** | <0.001 | 29.82  (43.70-19.44) | 56.50(93.38-31.61) | **2.09(1.98-2.20)** | <0.001 |
| Canada | 0.61  (0.71-0.51) | 0.86  (1.06-0.69) | **1.05**  **(0.71-1.40)** | <0.001 | 0.43  (0.51-0.37) | 0.52  (0.64-0.42) | **0.44**  **(0.11-0.77)** | 0.010 | 20.43  (24.10-17.33) | 24.76  30.55-19.97) | **0.64**  **(0.01-1.28)** | 0.047 |
| Central African Republic | 3.33  (5.01-1.86) | 2.47  (3.96-1.38) | **-0.96**  **(-1.09--0.83)** | <0.001 | 3.10  (4.69-1.73) | 2.30  (3.65-1.30) | **-0.96**  **(-1.09--0.83)** | <0.001 | 144.20  (218.13-80.35) | 106.92  (170.53-60.31) | **-0.96**  **(-1.09--0.82)** | <0.001 |
| Chad | 0.45  (0.65-0.31) | 0.97  (1.51-0.59) | **2.57**  **(2.15-2.99)** | <0.001 | 0.42  (0.60-0.29) | 0.90  (1.40-0.55) | **2.54**  **(2.27-2.82)** | <0.001 | 19.66  (27.92-13.35) | 41.86  (65.32-25.74) | **2.54**  **(2.26-2.81)** | <0.001 |
| Chile | 0.99  (1.17-0.83) | 0.42  (0.51-0.35) | **-2.81**  **(-3.43--2.19)** | <0.001 | 0.87  (1.03-0.73) | 0.32  (0.39-0.27) | **-3.26**  **(-3.90--2.62)** | <0.001 | 41.20  (48.97-34.40) | 15.59  (18.98-12.86) | **-3.22**  **(-3.85--2.59)** | <0.001 |
| China | 5.84  (7.01-4.78) | 2.61  (3.32-2.03) | **-2.61**  **(-2.85--2.38)** | <0.001 | 5.15  (6.20-4.23) | 1.79  (2.30-1.38) | **-3.43**  **(-3.77--3.08)** | <0.001 | 241.92  (290.94-199.00) | 84.81  (108.68-65.25) | **-3.40**  **(-3.74--3.06)** | <0.001 |
| Colombia | 0.72  (0.85-0.61) | 0.32  (0.42-0.24) | **-2.61**  **(-2.99--2.23)** | <0.001 | 0.65  (0.77-0.55) | 0.27  (0.35-0.20) | **-2.86**  **(-3.25--2.48)** | <0.001 | 31.81  (37.46-26.79) | 13.24  (17.21-9.98) | **-2.86**  **(-3.24--2.49)** | <0.001 |
| Comoros | 3.72  (5.61-2.07) | 2.49  (3.75-1.60) | **-1.22**  **(-1.85--0.58)** | <0.001 | 3.43  (5.17-1.92) | 2.28  (3.44-1.48) | **-1.24**  **(-1.87--0.61)** | <0.001 | 162.11  (244.46-89.35) | 107.76  (162.61-69.87) | **-1.23**  **(-1.89--0.57)** | <0.001 |
| Congo | 3.96  (6.11-2.32) | 2.19  (3.61-1.29) | **-1.87**  **(-2.32--1.42)** | <0.001 | 3.67  (5.74-2.21) | 2.01  (3.29-1.18) | **-1.92**  **(-2.38--1.46)** | <0.001 | 170.86  (267.01-102.28) | 94.03  (153.96-55.10) | **-1.89**  **(-2.34--1.43)** | <0.001 |
| Cook Islands | 0.57  (0.89-0.35) | 0.58  (0.92-0.34) | 0.03  (-0.39-0.45) | 0.890 | 0.50  (0.78-0.31) | 0.45  (0.72-0.27) | -0.35  (-0.77-0.07) | 0.100 | 24.09  (37.60-14.66) | 21.72  (34.87-12.83) | -0.33  (-0.74-0.07) | 0.108 |
| Costa Rica | 0.41  (0.50-0.34) | 0.31  (0.39-0.24) | **-1.21**  **(-1.76--0.67)** | <0.001 | 0.36  (0.43-0.30) | 0.25  (0.32-0.20) | **-1.28**  **(-1.79--0.78)** | <0.001 | 17.07  (20.57-14.05) | 12.22  (15.46-9.54) | **-1.22**  **(-1.72--0.72)** | <0.001 |
| Coted'Ivoire | 0.24  (0.34-0.16) | 0.25  (0.39-0.15) | 0.19  (-0.07-0.45) | 0.156 | 0.22  (0.32-0.15) | 0.23  (0.36-0.14) | 0.16  (-0.09-0.42) | 0.214 | 10.29  (14.78-6.87) | 10.78  (16.82-6.49) | 0.17  (-0.09-0.44) | 0.203 |
| Croatia | 1.02  (1.23-0.81) | 0.57  (0.74-0.43) | **-1.87**  **(-3.51--0.20)** | 0.028 | 0.90  (1.11-0.73) | 0.47  (0.62-0.36) | **-2.08**  **(-3.72--0.42)** | 0.014 | 41.55  (50.79-33.33) | 21.92  (28.71-16.70) | **-2.08**  **(-4.04--0.08)** | 0.042 |
| Cuba | 0.76  (0.90-0.64) | 1.09  (1.42-0.83) | **1.27**  **(0.06-2.50)** | 0.039 | 0.66  (0.79-0.56) | 0.91  (1.20-0.68) | 1.13  (-0.09-2.37) | 0.070 | 31.07  (37.14-26.16) | 41.52  (54.71-31.27) | 1.04  (-0.06-2.17) | 0.065 |
| Cyprus | 0.15  (0.21-0.10) | 0.24  (0.35-0.16) | **1.42**  **(0.99-1.85)** | <0.001 | 0.13  (0.18-0.09) | 0.16  (0.24-0.11) | **0.79**  **(0.11-1.48)** | 0.023 | 5.91  (8.32-4.03) | 7.49  (11.03-4.98) | **0.81**  **(0.14-1.49)** | 0.018 |
| Czechia | 0.89  (1.06-0.74) | 0.59  (0.75-0.45) | **-1.34**  **(-2.05--0.63)** | <0.001 | 0.79  (0.95-0.65) | 0.47  (0.60-0.36) | **-1.72**  **(-2.43--1.00)** | <0.001 | 36.36  (43.60-30.08) | 21.96  (28.01-16.83) | **-1.57**  **(-2.29--0.84)** | <0.001 |
| Democratic People's Republic of Korea | 2.57  (4.19-1.53) | 2.38  (3.90-1.36) | **-0.23**  **(-0.38--0.08)** | 0.003 | 2.30  (3.83-1.38) | 1.99  (3.25-1.13) | **-0.45**  **(-0.57--0.33)** | <0.001 | 108.04  (180.03-64.56) | 93.63  (153.46-52.85) | **-0.45**  **(-0.56--0.33)** | <0.001 |
| Democratic Republic of the Congo | 1.97  (3.03-1.20) | 1.50  (2.31-0.90) | **-0.88**  **(-1.08--0.67)** | <0.001 | 1.83  (2.83-1.11) | 1.38  (2.12-0.82) | **-0.90**  **(-1.11--0.70)** | <0.001 | 85.73  (132.59-51.64) | 64.42  (99.48-38.27) | **-0.91**  **(-1.11--0.71)** | <0.001 |
| Denmark | 0.99  (1.18-0.83) | 0.75  (0.91-0.61) | -0.71  (-2.14-0.76) | 0.342 | 0.83  (0.99-0.69) | 0.51  (0.63-0.41) | -1.36  (-2.78-0.09) | 0.065 | 38.47  (46.04-31.98) | 23.89  (29.38-19.22) | -1.34  (-2.75-0.09) | 0.066 |
| Djibouti | 3.10  (4.83-1.81) | 2.29  (3.68-1.36) | **-1.01**  **(-1.18--0.84)** | <0.001 | 2.86  (4.50-1.67) | 2.10  (3.38-1.23) | **-1.03**  **(-1.19--0.86)** | <0.001 | 133.49  (210.21-77.82) | 98.03  (158.38-57.46) | **-1.02**  **(-1.19--0.85)** | <0.001 |
| Dominica | 0.85  (1.14-0.61) | 0.98  (1.47-0.65) | **0.48**  **(0.33-0.63)** | <0.001 | 0.77  (1.04-0.56) | 0.89  (1.33-0.59) | **0.45**  **(0.30-0.59)** | <0.001 | 35.70  (48.21-25.87) | 41.15  (61.97-27.41) | **0.45**  **(0.29-0.60)** | <0.001 |
| Dominican Republic | 0.49  (0.66-0.36) | 0.61  (0.90-0.40) | 0.75  (-0.27-1.78) | 0.152 | 0.45  (0.60-0.32) | 0.55  (0.80-0.36) | 0.69  (-0.31-1.69) | 0.179 | 21.36  (28.61-15.35) | 25.77  (37.47-16.88) | 0.65  (-0.36-1.66) | 0.207 |
| Ecuador | 0.37  (0.45-0.31) | 0.21  (0.28-0.15) | **-1.95**  **(-3.09--0.80)** | 0.001 | 0.34  (0.41-0.28) | 0.18  (0.24-0.13) | **-1.97**  **(-3.17--0.76)** | 0.001 | 16.63  (19.94-13.73) | 8.97  (11.95-6.38) | **-1.94**  **(-3.15--0.72)** | 0.002 |
| Egypt | 0.31  (0.42-0.23) | 0.17  (0.25-0.12) | **-1.83**  **(-2.30--1.36)** | <0.001 | 0.28  (0.38-0.21) | 0.15  (0.22-0.10) | **-1.98**  **(-2.46--1.51)** | <0.001 | 13.45  (18.14-9.82) | 7.27  (10.36-4.91) | **-1.96**  **(-2.44--1.48)** | <0.001 |
| El Salvador | 0.46  (0.56-0.38) | 0.43  (0.57-0.31) | -0.31  (-1.27-0.67) | 0.536 | 0.42  (0.52-0.35) | 0.37  (0.49-0.27) | -0.48  (-1.46-0.51) | 0.342 | 20.59  (25.04-16.79) | 17.88  (23.85-13.01) | -0.51  (-1.48-0.47) | 0.309 |
| Equatorial Guinea | 3.06  (4.87-1.79) | 1.74  (2.88-0.97) | **-1.69**  **(-2.04--1.33)** | <0.001 | 2.85  (4.48-1.67) | 1.57  (2.64-0.85) | **-1.77**  **(-2.12--1.42)** | <0.001 | 132.32  (208.17-77.05) | 73.89  (124.05-39.83) | **-1.74**  **(-2.08--1.40)** | <0.001 |
| Eritrea | 5.21  (7.85-3.02) | 3.23  (5.26-1.87) | **-1.56**  **(-1.80--1.33)** | <0.001 | 4.84  (7.29-2.80) | 2.98  (4.86-1.72) | **-1.59**  **(-1.82--1.35)** | <0.001 | 224.78  (339.17-129.81) | 139.48  (227.59-80.39) | **-1.56**  **(-1.79--1.33)** | <0.001 |
| Estonia | 0.87  (1.06-0.70) | 0.42  (0.55-0.32) | **-2.19**  **(-2.97--1.40)** | <0.001 | 0.77  (0.94-0.62) | 0.33  (0.43-0.25) | **-2.58**  **(-3.33--1.81)** | <0.001 | 35.38  (43.07-28.33) | 15.12  (19.47-11.26) | **-2.62**  **(-3.37--1.87)** | <0.001 |
| Eswatini | 3.77  (5.55-2.34) | 4.90  (7.71-2.81) | **0.87**  **(0.51-1.24)** | <0.001 | 3.48  (5.09-2.15) | 4.51  (7.16-2.49) | **0.86**  **(0.49-1.24)** | <0.001 | 160.44  (235.08-99.33) | 208.56  (330.71-115.09) | **0.87**  **(0.50-1.24)** | <0.001 |
| Ethiopia | 1.98  (2.70-1.23) | 0.89  (1.23-0.66) | **-2.54**  **(-2.70--2.37)** | <0.001 | 1.85  (2.53-1.15) | 0.82  (1.12-0.61) | **-2.59**  **(-2.73--2.46)** | <0.001 | 86.43  (118.54-53.66) | 38.71  (53.18-28.79) | **-2.56**  **(-2.68--2.43)** | <0.001 |
| Fiji | 0.51  (0.75-0.33) | 0.53  (0.83-0.32) | 0.08  (-0.19-0.36) | 0.555 | 0.46  (0.67-0.30) | 0.47  (0.75-0.28) | 0.02  (-0.24-0.29) | 0.866 | 22.07  (32.47-14.21) | 22.57  (35.99-13.51) | 0.04  (-0.22-0.31) | 0.756 |
| Finland | 0.92  (1.03-0.81) | 0.70  (0.80-0.61) | -0.63  (-1.34-0.07) | 0.079 | 0.65  (0.73-0.58) | 0.38  (0.43-0.33) | **-1.56**  **(-2.19--0.93)** | <0.001 | 30.75  (34.70-27.42) | 17.96  (20.35-15.76) | **-1.55**  **(-2.16--0.94)** | <0.001 |
| France | 2.50  (2.98-2.09) | 0.99  (1.25-0.78) | **-2.84**  **(-3.48--2.20)** | <0.001 | 1.95  (2.33-1.62) | 0.50  (0.63-0.40) | **-4.25**  **(-4.94--3.56)** | <0.001 | 90.31  (107.70-74.66) | 23.85  (29.93-18.88) | **-4.16**  **(-4.86--3.46)** | <0.001 |
| Gabon | 2.80  (4.22-1.78) | 2.06  (3.34-1.19) | **-0.96**  **(-1.23--0.68)** | <0.001 | 2.59  (3.89-1.66) | 1.88  (3.02-1.06) | **-1.00**  **(-1.27--0.73)** | <0.001 | 120.40  (181.23-76.65) | 87.46  (141.09-49.30) | **-0.94**  **(-1.28--0.60)** | <0.001 |
| Gambia | 0.28  (0.41-0.18) | 0.37  (0.55-0.23) | 0.89  (-0.27-2.07) | 0.134 | 0.26  (0.38-0.17) | 0.34  (0.51-0.21) | 0.91  (-0.36-2.18) | 0.161 | 12.06  (17.87-7.69) | 15.79  (23.81-9.97) | 0.87  (-0.32-2.08) | 0.152 |
| Georgia | 0.46  (0.52-0.41) | 0.18  (0.21-0.15) | **-2.72**  **(-5.14--0.23)** | 0.033 | 0.42  (0.47-0.37) | 0.16  (0.19-0.14) | **-2.74**  **(-5.10--0.32)** | 0.027 | 19.88  (22.51-17.68) | 7.57  (8.79-6.51) | **-2.87**  **(-5.06--0.62)** | 0.013 |
| Germany | 1.31  (1.57-1.07) | 0.89  (1.09-0.72) | **-1.24**  **(-2.34--0.13)** | 0.029 | 1.02  (1.22-0.84) | 0.52  (0.64-0.42) | **-2.19**  **(-3.22--1.16)** | <0.001 | 46.82  (56.04-38.49) | 24.19  (29.98-19.53) | **-2.14**  **(-3.17--1.10)** | <0.001 |
| Ghana | 0.53  (0.77-0.34) | 0.65  (0.97-0.40) | **0.66**  **(0.46-0.86)** | <0.001 | 0.49  (0.71-0.31) | 0.59  (0.88-0.36) | **0.63**  **(0.43-0.82)** | <0.001 | 23.21  (33.95-14.76) | 28.14  (42.05-17.19) | **0.62**  **(0.43-0.82)** | <0.001 |
| Greece | 0.36  (0.41-0.32) | 0.43  (0.50-0.38) | 0.63  (-0.04-1.30) | 0.065 | 0.28  (0.32-0.25) | 0.30  (0.34-0.26) | 0.27  (-0.43-0.97) | 0.457 | 13.31  (15.05-11.77) | 14.07  (16.08-12.29) | 0.24  (-0.45-0.93) | 0.495 |
| Greenland | 3.69  (5.22-2.56) | 1.99  (2.87-1.29) | **-1.96**  **(-2.03--1.89)** | <0.001 | 3.30  (4.65-2.30) | 1.67  (2.42-1.08) | **-2.18**  **(-2.25--2.12)** | <0.001 | 153.97  (217.48-107.46) | 77.39  (112.37-49.92) | **-2.14**  **(-2.29--1.99)** | <0.001 |
| Grenada | 1.58  (1.99-1.24) | 0.99  (1.31-0.73) | **-1.77**  **(-3.36--0.15)** | 0.032 | 1.44  (1.81-1.13) | 0.88  (1.17-0.65) | **-1.84**  **(-3.41--0.24)** | 0.024 | 67.82  (85.04-53.35) | 41.15  (54.65-30.34) | **-1.88**  **(-3.44--0.29)** | 0.021 |
| Guam | 0.45  (0.59-0.34) | 0.76  (1.00-0.56) | **1.62**  **(0.84-2.40)** | <0.001 | 0.38  (0.50-0.29) | 0.62  (0.82-0.46) | **1.45**  **(0.52-2.38)** | 0.002 | 18.54  (24.11-14.10) | 30.00  (39.32-22.19) | **1.59**  **(0.51-2.68)** | 0.004 |
| Guatemala | 0.63  (0.69-0.57) | 0.35  (0.42-0.28) | **-1.89**  **(-2.86--0.92)** | <0.001 | 0.58  (0.63-0.52) | 0.31  (0.37-0.25) | **-1.97**  **(-2.94--0.99)** | <0.001 | 28.30  (31.06-25.53) | 15.04  (17.99-12.28) | **-1.95**  **(-2.99--0.90)** | <0.001 |
| Guinea | 0.22  (0.31-0.15) | 0.25  (0.39-0.15) | **0.40**  **(0.21-0.59)** | <0.001 | 0.21  (0.29-0.14) | 0.23  (0.36-0.14) | **0.38**  **(0.20-0.57)** | <0.001 | 9.58  (13.61-6.37) | 10.81  (16.79-6.67) | **0.39**  **(0.20-0.58)** | <0.001 |
| Guinea-Bissau | 1.04  (1.54-0.65) | 1.61  (2.49-0.98) | **1.49**  **(1.30-1.67)** | <0.001 | 0.97  (1.45-0.62) | 1.50  (2.30-0.91) | **1.47**  **(1.29-1.65)** | <0.001 | 44.99  (67.65-28.65) | 69.98  (107.58-42.56) | **1.48**  **(1.31-1.65)** | <0.001 |
| Guyana | 0.48  (0.59-0.38) | 0.60  (0.85-0.41) | **0.74**  **(0.06-1.43)** | 0.034 | 0.44  (0.55-0.35) | 0.55  (0.78-0.37) | **0.70**  **(0.01-1.40)** | 0.048 | 20.50  (25.70-16.29) | 25.63  (36.45-17.44) | **0.73**  **(0.03-1.43)** | 0.040 |
| Haiti | 1.21  (1.80-0.74) | 0.87  (1.35-0.53) | **-0.98**  **(-1.09--0.87)** | <0.001 | 1.13  (1.67-0.68) | 0.81  (1.24-0.49) | **-1.01**  **(-1.12--0.90)** | <0.001 | 52.97  (78.56-31.88) | 37.91  (58.33-22.85) | **-1.01**  **(-1.12--0.89)** | <0.001 |
| Honduras | 0.23  (0.32-0.17) | 0.18  (0.29-0.11) | **-0.85**  **(-1.04--0.66)** | <0.001 | 0.21  (0.29-0.15) | 0.16  (0.26-0.10) | **-0.90**  **(-1.09--0.71)** | <0.001 | 10.42  (14.23-7.46) | 7.70  (12.25-4.49) | **-0.99**  **(-1.19--0.80)** | <0.001 |
| Hungary | 1.93  (2.33-1.58) | 0.57  (0.71-0.44) | **-3.97**  **(-4.66--3.28)** | <0.001 | 1.75  (2.13-1.43) | 0.49  (0.62-0.39) | **-4.11**  **(-4.80--3.41)** | <0.001 | 80.64  (98.28-66.09) | 22.97  (28.82-18.04) | **-4.08**  **(-4.76--3.40)** | <0.001 |
| Iceland | 0.88  (1.04-0.73) | 1.17  (1.48-0.91) | **1.05**  **(0.66-1.43)** | <0.001 | 0.66  (0.79-0.55) | 0.71  (0.89-0.56) | 0.30  (-0.04-0.65) | 0.086 | 30.71  (36.63-25.38) | 33.47  (41.93-26.32) | **0.38**  **(0.04-0.73)** | 0.030 |
| India | 1.08  (1.32-0.94) | 0.86  (1.02-0.74) | **-0.71**  **(-0.86--0.56)** | <0.001 | 1.00  (1.22-0.86) | 0.77  (0.91-0.66) | **-0.81**  (-0.96--0.66) | <0.001 | 47.60  (58.26-41.20) | 36.59  (43.67-31.57) | **-0.82**  **(-0.98--0.65)** | <0.001 |
| Indonesia | 0.52  (0.64-0.38) | 0.36  (0.48-0.27) | **-1.13**  **(-1.22--1.05)** | <0.001 | 0.47  (0.58-0.34) | 0.32  (0.42-0.24) | **-1.26**  **(-1.34--1.18)** | <0.001 | 22.85  (28.24-16.37) | 15.22  (20.17-11.44) | **-1.29**  **(-1.37--1.21)** | <0.001 |
| Iran (Islamic Republic of) | 1.02  (1.17-0.83) | 0.66  (0.75-0.59) | **-1.43**  **(-1.52--1.34)** | <0.001 | 0.91  (1.05-0.74) | 0.55  (0.62-0.48) | **-1.68**  **(-1.76--1.61)** | <0.001 | 44.02  (50.60-35.74) | 26.68  (30.16-23.54) | **-1.61**  **(-1.79--1.42)** | <0.001 |
| Iraq | 0.31  (0.43-0.20) | 0.23  (0.35-0.15) | **-0.91**  **(-1.08--0.74)** | <0.001 | 0.28  (0.39-0.19) | 0.20  (0.30-0.13) | **-1.11**  **(-1.36--0.86)** | <0.001 | 12.91  (18.30-8.69) | 9.26  (14.03-6.09) | **-1.10**  **(-1.37--0.84)** | <0.001 |
| Ireland | 1.26  (1.53-1.05) | 1.09  (1.36-0.87) | -0.34  (-1.36-0.69) | 0.519 | 1.02  (1.23-0.84) | 0.67  (0.83-0.53) | **-1.28**  **(-2.28--0.27)** | 0.013 | 47.63  (57.65-39.35) | 31.49  (39.25-25.11) | **-1.25**  **(-2.23--0.27)** | 0.013 |
| Israel | 0.31  (0.38-0.26) | 0.27  (0.34-0.22) | -0.37  (-0.95-0.21) | 0.214 | 0.26  (0.31-0.21) | 0.19  (0.23-0.15) | **-0.95**  **(-1.50--0.39)** | 0.001 | 12.15  (14.69-10.00) | 8.94  (11.00-7.13) | **-0.95**  **(-1.50--0.39)** | 0.001 |
| Italy | 0.78  (0.84-0.73) | 0.35  (0.38-0.32) | **-2.71**  **(-3.28--2.13)** | <0.001 | 0.64  (0.68-0.60) | 0.24  (0.26-0.22) | **-3.27**  **(-3.82--2.72)** | <0.001 | 29.87  (32.05-27.92) | 11.24  (12.05-10.49) | **-3.25**  **(-3.80--2.71)** | <0.001 |
| Jamaica | 0.47  (0.57-0.38) | 0.60  (0.86-0.39) | 1.77  (-0.31-3.90) | 0.096 | 0.42  (0.51-0.34) | 0.52  (0.74-0.34) | 1.70  (-0.40-3.84) | 0.113 | 19.32  (23.47-15.82) | 24.29  (34.54-16.09) | 1.71  (-0.37-3.83) | 0.108 |
| Japan | 0.93  (0.98-0.88) | 0.69  (0.74-0.65) | **-1.00**  **(-1.57--0.42)** | 0.001 | 0.57  (0.59-0.55) | 0.28  (0.29-0.27) | **-2.33**  **(-2.61--2.05)** | <0.001 | 26.09  (27.05-25.14) | 13.36  (13.86-12.89) | **-2.26**  **(-2.53--1.98)** | <0.001 |
| Jordan | 0.23  (0.31-0.16) | 0.16  (0.24-0.11) | **-1.05**  **(-1.53--0.57)** | <0.001 | 0.20  (0.28-0.14) | 0.13  (0.20-0.09) | **-1.33**  **(-1.79--0.86)** | <0.001 | 9.58  (13.09-6.58) | 6.37  (9.37-4.14) | **-1.30**  **(-1.77--0.82)** | <0.001 |
| Kazakhstan | 3.64  (3.98-3.32) | 0.81  (0.92-0.70) | **-4.96**  **(-5.12--4.80)** | <0.001 | 3.35  (3.66-3.06) | 0.72  (0.82-0.63) | **-5.05**  **(-5.21--4.88)** | <0.001 | 157.86  (172.33-144.06) | 34.33  (38.75-29.88) | **-4.91**  **(-5.30--4.51)** | <0.001 |
| Kenya | 1.69  (2.43-1.22) | 2.08  (2.95-1.51) | **0.68**  **(0.47-0.88)** | <0.001 | 1.55  (2.23-1.12) | 1.89  (2.68-1.37) | **0.65**  **(0.44-0.86)** | <0.001 | 73.45  (105.64-53.07) | 89.22  (127.18-64.47) | **0.63**  **(0.42-0.84)** | <0.001 |
| Kiribati | 1.66  (2.53-1.03) | 1.63  (2.63-0.93) | -0.04  (-0.12-0.05) | 0.426 | 1.52  (2.31-0.93) | 1.49  (2.39-0.86) | -0.07  (-0.16-0.02) | 0.138 | 74.06  (112.28-45.28) | 72.67  (117.09-41.68) | -0.05  (-0.14-0.03) | 0.229 |
| Kuwait | 0.30  (0.36-0.25) | 0.15  (0.20-0.11) | **-1.98**  **(-3.96-0.05)** | 0.056 | 0.25  (0.30-0.21) | 0.11  (0.15-0.08) | **-2.33**  **(-4.32--0.30)** | 0.024 | 11.50  (13.84-9.47) | 5.38  (7.16-3.99) | **-2.35**  **(-3.73--0.96)** | 0.001 |
| Kyrgyzstan | 2.08  (2.51-1.71) | 0.47  (0.61-0.35) | **-4.72**  **(-5.54--3.89)** | <0.001 | 1.92  (2.31-1.57) | 0.42  (0.54-0.31) | **-4.80**  **(-5.26--4.34)** | <0.001 | 91.04  (109.73-74.40) | 20.15  (25.91-14.91) | **-4.72**  **(-5.39--4.04)** | <0.001 |
| Lao People's Democratic Republic | 1.26  (1.90-0.76) | 0.61  (0.95-0.38) | **-2.32**  **(-2.40--2.25)** | <0.001 | 1.16  (1.77-0.70) | 0.55  (0.85-0.34) | **-2.42**  **(-2.49--2.34)** | <0.001 | 55.47  (84.51-33.32) | 26.41  (41.28-16.39) | **-2.37**  **(-2.45--2.30)** | <0.001 |
| Latvia | 0.85  (1.05-0.68) | 0.54  (0.70-0.41) | -1.38  (-2.90-0.17) | 0.080 | 0.77  (0.95-0.62) | 0.47  (0.61-0.36) | -1.49  (-3.02-0.05) | 0.058 | 35.42  (43.78-28.31) | 21.58  (27.99-16.30) | -1.52  (-3.07-0.06) | 0.059 |
| Lebanon | 0.25  (0.37-0.16) | 0.15  (0.22-0.10) | **-1.47**  **(-1.80--1.14)** | <0.001 | 0.22  (0.32-0.15) | 0.12  (0.17-0.08) | **-1.82**  **(-2.15--1.50)** | <0.001 | 10.28  (15.20-6.83) | 5.74  (8.21-3.80) | **-1.81**  **(-2.14--1.49)** | <0.001 |
| Lesotho | 2.04  (2.99-1.33) | 4.22  (6.35-2.54) | **2.41**  **(2.08-2.74)** | <0.001 | 1.88  (2.75-1.23) | 3.90  (5.88-2.34) | **2.42**  **(2.08-2.75)** | <0.001 | 86.97  (127.27-56.65) | 179.93  (272.11-107.94) | **2.41**  **(2.08-2.74)** | <0.001 |
| Liberia | 0.55  (0.80-0.36) | 1.12  (1.80-0.65) | **2.39**  **(2.06-2.72)** | <0.001 | 0.51  (0.74-0.34) | 1.03  (1.67-0.60) | **2.36**  **(2.03-2.69)** | <0.001 | 23.76  (34.50-15.61) | 48.46  (78.23-27.94) | **2.37**  **(2.04-2.71)** | <0.001 |
| Libya | 0.28  (0.43-0.18) | 0.36  (0.55-0.23) | **0.78**  **(0.63-0.92)** | <0.001 | 0.26  (0.39-0.16) | 0.32  (0.48-0.20) | **0.67**  **(0.53-0.82)** | <0.001 | 12.03  (18.49-7.45) | 14.79  (22.54-9.18) | **0.66**  **(0.51-0.81)** | <0.001 |
| Lithuania | 0.96  (1.16-0.78) | 0.78  (1.01-0.60) | -0.59  (-2.30-1.16) | 0.508 | 0.84  (1.03-0.69) | 0.66  (0.86-0.51) | -0.72  (-2.47-1.06) | 0.424 | 38.85  (47.36-31.61) | 29.91  (38.90-23.12) | -0.78  (-2.51-0.99) | 0.387 |
| Luxembourg | 1.27  (1.47-1.10) | 0.62  (0.74-0.52) | **-2.40**  **(-2.95--1.84)** | <0.001 | 1.05  (1.22-0.91) | 0.41  (0.49-0.34) | **-3.13**  **(-3.64--2.63)** | <0.001 | 48.80  (56.44-42.31) | 18.83  (22.40-15.84) | **-3.18**  **(-3.65--2.71)** | <0.001 |
| Madagascar | 3.38  (4.89-2.24) | 2.30  (3.58-1.41) | **-1.22**  **(-1.37--1.08)** | <0.001 | 3.12  (4.53-2.06) | 2.12  (3.30-1.29) | **-1.24**  **(-1.38--1.10)** | <0.001 | 147.69  (214.90-96.79) | 100.37  (156.51-60.93) | **-1.23**  **(-1.38--1.09)** | <0.001 |
| Malawi | 5.39  (7.46-3.81) | 6.27  (9.43-4.19) | **0.50**  **(0.28-0.71)** | <0.001 | 4.99  (6.87-3.53) | 5.76  (8.45-3.86) | **0.47**  **(0.26-0.68)** | <0.001 | 235.89  (325.11-166.23) | 271.01  (399.36-180.94) | **0.45**  **(0.24-0.67)** | <0.001 |
| Malaysia | 0.45  (0.61-0.32) | 0.53  (0.75-0.37) | 0.57  (-0.29-1.44) | 0.192 | 0.40  (0.55-0.29) | 0.44  (0.62-0.31) | 0.30  (-0.58-1.18) | 0.507 | 18.88  (25.60-13.40) | 20.45  (28.82-14.23) | 0.39  (-0.13-0.92) | 0.142 |
| Maldives | 0.84  (1.24-0.48) | 0.26  (0.38-0.17) | **-3.83**  **(-4.07--3.60)** | <0.001 | 0.76  (1.12-0.44) | 0.21  (0.30-0.13) | **-4.24**  **(-4.45--4.02)** | <0.001 | 36.27  (53.24-20.61) | 9.87  (14.53-6.36) | **-4.20**  **(-4.42--3.98)** | <0.001 |
| Mali | 0.47  (0.64-0.32) | 0.45  (0.68-0.28) | -0.09  (-0.33-0.16) | 0.486 | 0.43  (0.59-0.30) | 0.41  (0.62-0.26) | -0.12  (-0.36-0.13) | 0.347 | 20.18  (27.62-13.81) | 19.27  (29.00-12.26) | -0.11  (-0.35-0.13) | 0.365 |
| Malta | 0.46  (0.57-0.37) | 0.59  (0.74-0.46) | 0.76  (-0.49-2.02) | 0.235 | 0.38  (0.47-0.31) | 0.40  (0.50-0.31) | **0.27**  **(0.04-0.49)** | 0.021 | 17.82  (21.84-14.37) | 18.38  (23.20-14.33) | **0.28**  **(0.06-0.49)** | 0.014 |
| Marshall Islands | 0.65  (1.01-0.39) | 0.70  (1.11-0.40) | **0.29**  **(0.15-0.42)** | <0.001 | 0.59  (0.93-0.35) | 0.62  (0.99-0.37) | **0.22**  **(0.09-0.36)** | 0.001 | 28.62  (44.81-17.05) | 30.43  (48.22-18.02) | **0.25**  **(0.12-0.38)** | <0.001 |
| Mauritania | 0.63  (0.91-0.39) | 0.81  (1.32-0.48) | **0.86**  **(0.57-1.14)** | <0.001 | 0.58  (0.84-0.37) | 0.74  (1.19-0.44) | **0.79**  **(0.50-1.08)** | <0.001 | 27.00  (39.34-17.02) | 34.53  (55.51-20.36) | **0.80**  **(0.50-1.10)** | <0.001 |
| Mauritius | 0.91  (1.01-0.81) | 1.13  (1.29-0.97) | 1.10  (-0.69-2.92) | 0.231 | 0.79  (0.88-0.71) | 0.93  (1.06-0.79) | 0.90  (-0.89-2.72) | 0.329 | 37.66  (41.89-33.60) | 43.91  (50.21-37.51) | 0.89  (-0.91-2.71) | 0.335 |
| Mexico | 0.44  (0.45-0.43) | 0.35  (0.40-0.30) | **-0.71**  **(-0.95--0.48)** | <0.001 | 0.40  (0.41-0.39) | 0.30  (0.35-0.26) | **-0.82**  **(-1.06--0.59)** | <0.001 | 19.38  (19.99-18.82) | 14.75  (16.77-12.93) | **-0.82**  **(-1.06--0.59)** | <0.001 |
| Micronesia (Federated States of) | 0.88  (1.37-0.54) | 0.77  (1.21-0.45) | **-0.46**  **(-0.52--0.40)** | <0.001 | 0.81  (1.24-0.50) | 0.68  (1.10-0.41) | **-0.53**  **(-0.59--0.47)** | <0.001 | 39.06  (60.05-24.14) | 33.22  (53.63-19.69) | **-0.53**  **(-0.57--0.49)** | <0.001 |
| Monaco | 1.16  (1.67-0.79) | 1.63  (2.43-0.98) | **1.11**  **(1.04-1.17)** | <0.001 | 0.86  (1.24-0.60) | 1.03  (1.55-0.62) | **0.59**  **(0.46-0.71)** | <0.001 | 40.74  (58.45-28.02) | 49.11  (73.71-29.43) | **0.60**  **(0.48-0.72)** | <0.001 |
| Mongolia | 4.31  (5.96-3.05) | 2.54  (3.43-1.80) | **-1.86**  **(-2.88--0.83)** | <0.001 | 4.00  (5.51-2.83) | 2.33  (3.12-1.65) | **-1.90**  **(-2.92--0.87)** | <0.001 | 190.99  (264.05-135.11) | 110.27  (148.36-78.13) | **-1.88**  **(-2.80--0.96)** | <0.001 |
| Montenegro | 0.60  (0.75-0.47) | 0.51  (0.67-0.38) | -0.58  (-1.19-0.03) | 0.063 | 0.53  (0.66-0.42) | 0.44  (0.58-0.33) | **-0.62**  **(-1.24--0.00)** | 0.049 | 24.52  (30.88-19.38) | 20.56  (26.70-15.44) | **-0.64**  **(-1.24--0.05)** | 0.035 |
| Morocco | 0.17  (0.23-0.11) | 0.14  (0.22-0.09) | **-0.56**  **(-0.68--0.44)** | <0.001 | 0.15  (0.21-0.10) | 0.12  (0.19-0.08) | **-0.68**  **(-0.80--0.57)** | <0.001 | 7.13  (9.92-4.91) | 5.70  (9.10-3.58) | **-0.72**  **(-0.84--0.60)** | <0.001 |
| Mozambique | 0.86  (1.25-0.57) | 1.28  (2.01-0.77) | **1.33**  **(1.15-1.52)** | <0.001 | 0.80  (1.15-0.53) | 1.19  (1.87-0.72) | **1.31**  **(1.13-1.50)** | <0.001 | 36.68  (53.10-24.56) | 55.00  (86.61-33.25) | **1.32**  **(1.11-1.52)** | <0.001 |
| Myanmar | 1.17  (1.70-0.72) | 0.55  (0.83-0.35) | **-2.43**  **(-2.56--2.30)** | <0.001 | 1.08  (1.57-0.66) | 0.49  (0.75-0.31) | **-2.55**  **(-2.67--2.42)** | <0.001 | 52.09  (76.21-31.67) | 23.55  (36.19-14.85) | **-2.55**  **(-2.67--2.42)** | <0.001 |
| Namibia | 0.54  (0.80-0.35) | 0.59  (0.96-0.35) | **0.36**  **(0.01-0.72)** | 0.045 | 0.50  (0.73-0.33) | 0.54  (0.88-0.32) | 0.31  (-0.04-0.66) | 0.078 | 23.38  (34.10-15.15) | 25.04  (41.13-14.77) | 0.31  (-0.03-0.65) | 0.075 |
| Nauru | 1.09  (1.68-0.65) | 1.00  (1.59-0.58) | **-0.26**  **(-0.33--0.19)** | <0.001 | 0.99  (1.55-0.59) | 0.89  (1.43-0.52) | **-0.33**  **(-0.40--0.25)** | <0.001 | 47.69  (74.57-28.39) | 43.25  (69.28-25.31) | **-0.31**  **(-0.38--0.24)** | <0.001 |
| Nepal | 1.31  (1.95-0.84) | 0.94  (1.46-0.59) | **-1.05**  **(-1.20--0.89)** | <0.001 | 1.21  (1.81-0.78) | 0.85  (1.33-0.53) | **-1.12**  **(-1.27--0.96)** | <0.001 | 57.58  (86.02-36.94) | 40.47  (63.39-25.27) | **-1.11**  **(-1.27--0.96)** | <0.001 |
| Netherlands | 1.03  (1.21-0.87) | 0.84  (1.02-0.68) | -0.64  (-1.31-0.04) | 0.064 | 0.83  (0.97-0.70) | 0.54  (0.66-0.44) | **-1.33**  **(-1.93--0.72)** | <0.001 | 38.39  (45.11-32.36) | 25.37  (31.03-20.69) | **-1.31**  **(-1.90--0.71)** | <0.001 |
| New Zealand | 0.61  (0.72-0.51) | 0.56  (0.67-0.47) | -0.33  (-0.66-0.00) | 0.051 | 0.44  (0.52-0.37) | 0.33  (0.39-0.28) | **-0.91**  **(-1.41--0.40)** | <0.001 | 20.64  (24.40-17.43) | 15.45  (18.34-12.94) | **-0.91**  **(-1.43--0.38)** | 0.001 |
| Nicaragua | 0.21  (0.28-0.15) | 0.16  (0.22-0.11) | **-1.29**  **(-1.83--0.74)** | <0.001 | 0.19  (0.26-0.14) | 0.14  (0.20-0.09) | **-1.42**  **(-1.95--0.88)** | <0.001 | 9.26  (12.46-6.79) | 6.59  (9.41-4.54) | **-1.42**  **(-1.96--0.88)** | <0.001 |
| Niger | 0.50  (0.73-0.32) | 0.65  (1.03-0.39) | **0.87**  **(0.53-1.21)** | <0.001 | 0.46  (0.68-0.30) | 0.60  (0.96-0.37) | **0.86**  **(0.51-1.21)** | <0.001 | 21.59  (31.90-13.95) | 28.28  (45.22-17.17) | **0.87**  **(0.57-1.17)** | <0.001 |
| Nigeria | 0.49  (0.71-0.35) | 0.74  (1.06-0.46) | **1.27**  **(1.05-1.49)** | <0.001 | 0.46  (0.66-0.32) | 0.68  (0.98-0.42) | **1.23**  **(1.02-1.45)** | <0.001 | 21.38  (30.52-14.99) | 31.85  (45.82-19.69) | **1.26**  **(1.05-1.47)** | <0.001 |
| Niue | 0.53  (0.86-0.31) | 0.52  (0.85-0.31) | -0.09  (-0.42-0.24) | 0.589 | 0.48  (0.76-0.28) | 0.45  (0.73-0.26) | -0.23  (-0.55-0.10) | 0.170 | 23.03  (37.04-13.45) | 21.77  (35.51-12.90) | -0.19  (-0.48-0.11) | 0.21 |
| North Macedonia | 0.35  (0.44-0.28) | 0.25  (0.35-0.18) | **-1.20**  **(-2.05--0.35)** | 0.006 | 0.32  (0.40-0.26) | 0.23  (0.31-0.16) | **-1.26**  **(-2.12--0.40)** | 0.004 | 15.05  (18.74-12.03) | 10.41  (14.28-7.40) | **-1.32**  **(-2.17--0.47)** | 0.002 |
| Northern Mariana Islands | 0.31  (0.51-0.18) | 0.51  (0.76-0.33) | **1.74**  **(0.75-2.74)** | 0.001 | 0.27  (0.44-0.16) | 0.42  (0.63-0.27) | **1.63**  **(0.65-2.61)** | 0.001 | 12.81  (20.86-7.61) | 20.26  (30.49-13.25) | **1.62**  **(0.72-2.54)** | <0.001 |
| Norway | 0.43  (0.46-0.40) | 0.43  (0.47-0.39) | 0.09  (-0.79-0.99) | 0.836 | 0.34  (0.36-0.31) | 0.28  (0.30-0.25) | **-0.51**  **(-0.80--0.22)** | 0.001 | 15.75  (16.84-14.68) | 12.87  (14.00-11.79) | **-0.48**  **(-0.78--0.19)** | 0.001 |
| Oman | 0.28  (0.43-0.18) | 0.20  (0.30-0.13) | **-1.21**  **(-1.49--0.92)** | <0.001 | 0.25  (0.38-0.16) | 0.16  (0.25-0.11) | **-1.50**  **(-1.76--1.24)** | <0.001 | 11.84  (17.87-7.64) | 7.67  (11.52-4.96) | **-1.47**  **(-1.73--1.21)** | <0.001 |
| Pakistan | 1.46  (1.86-1.13) | 1.48  (2.03-1.09) | 0.05  (-0.08-0.18) | 0.449 | 1.35  (1.72-1.04) | 1.35  (1.86-0.99) | 0.02  (-0.11-0.15) | 0.781 | 63.77  (81.78-49.27) | 64.28  (88.91-47.11) | 0.04  (-0.09-0.17) | 0.53 |
| Palau | 0.74  (1.16-0.46) | 0.84  (1.28-0.53) | **0.45**  **(0.03-0.87)** | 0.037 | 0.65  (1.03-0.42) | 0.72  (1.11-0.45) | 0.33  (-0.09-0.75) | 0.122 | 31.93  (50.26-20.29) | 35.58  (54.57-21.98) | **0.35**  **(0.04-0.66)** | 0.028 |
| Palestine | 0.23  (0.35-0.14) | 0.13  (0.18-0.09) | **-1.95**  **(-2.29--1.62)** | <0.001 | 0.20  (0.31-0.13) | 0.11  (0.15-0.08) | **-2.13**  **(-2.45--1.80)** | <0.001 | 9.79  (14.89-6.22) | 5.19  (7.21-3.65) | **-2.09**  **(-2.37--1.81)** | <0.001 |
| Panama | 0.31  (0.35-0.27) | 0.22  (0.28-0.17) | **-1.29**  **(-1.81--0.77)** | <0.001 | 0.28  (0.31-0.24) | 0.19  (0.24-0.14) | **-1.45**  **(-1.98--0.92)** | <0.001 | 13.28  (14.89-11.75) | 9.02  (11.43-6.79) | **-1.42**  **(-1.95--0.89)** | <0.001 |
| Papua New Guinea | 0.43  (0.72-0.24) | 0.33  (0.53-0.20) | **-0.86**  **(-1.02--0.70)** | <0.001 | 0.39  (0.65-0.22) | 0.30  (0.48-0.18) | **-0.89**  **(-1.05--0.73)** | <0.001 | 19.13  (31.93-10.60) | 14.63  (23.42-8.91) | **-0.87**  **(-1.03--0.71)** | <0.001 |
| Paraguay | 0.68  (0.95-0.47) | 0.80  (1.22-0.50) | **0.69**  **(0.46-0.92)** | <0.001 | 0.62  (0.86-0.43) | 0.71  (1.07-0.44) | **0.62**  **(0.39-0.85)** | <0.001 | 28.82  (40.18-20.15) | 33.00  (49.73-20.47) | **0.64**  **(0.29-1.00)** | <0.001 |
| Peru | 0.32  (0.44-0.24) | 0.21  (0.31-0.14) | -1.29  (-2.76-0.20) | 0.090 | 0.30  (0.40-0.22) | 0.18  (0.26-0.11) | **-1.53**  **(-2.99--0.05)** | 0.043 | 14.39  (19.32-10.47) | 8.66  (12.80-5.56) | **-1.54**  **(-2.99--0.06)** | 0.042 |
| Philippines | 0.49  (0.58-0.42) | 0.40  (0.48-0.33) | **-0.70**  **(-0.88--0.52)** | <0.001 | 0.45  (0.52-0.38) | 0.35  (0.42-0.29) | **-0.77**  **(-0.95--0.59)** | <0.001 | 21.88  (25.59-18.49) | 17.00  (20.47-13.96) | **-0.82**  **(-1.14--0.50)** | <0.001 |
| Poland | 1.02  (1.08-0.97) | 0.59  (0.64-0.52) | **-1.85**  **(-2.69--0.99)** | <0.001 | 0.95  (1.00-0.91) | 0.53  (0.58-0.48) | **-1.92**  **(-2.77--1.06)** | <0.001 | 43.89  (46.11-41.78) | 24.60  (26.99-22.05) | **-1.82**  **(-2.20--1.44)** | <0.001 |
| Portugal | 1.18  (1.44-0.98) | 0.82  (1.02-0.66) | **-1.03**  **(-1.42--0.64)** | <0.001 | 1.04  (1.26-0.85) | 0.62  (0.78-0.49) | **-1.52**  **(-1.91--1.13)** | <0.001 | 48.91  (59.54-40.14) | 28.90  (36.07-22.84) | **-1.61**  **(-2.00--1.23)** | <0.001 |
| Puerto Rico | 1.38  (1.68-1.12) | 0.70  (0.93-0.51) | **-2.06**  **(-2.97--1.13)** | <0.001 | 1.21  (1.48-0.98) | 0.56  (0.75-0.41) | **-2.34**  **(-3.25--1.42)** | <0.001 | 56.86  (69.68-46.12) | 26.38  (35.46-19.33) | **-2.52**  **(-3.32--1.72)** | <0.001 |
| Qatar | 0.46  (0.65-0.31) | 0.28  (0.42-0.17) | **-1.53**  **(-2.63--0.42)** | 0.007 | 0.40  (0.57-0.27) | 0.21  (0.33-0.13) | **-1.95**  **(-3.04--0.85)** | 0.001 | 19.35  (27.49-13.06) | 10.22  (15.66-6.30) | **-1.96**  **(-3.06--0.85)** | 0.001 |
| Republic of Korea | 1.10  (1.50-0.81) | 0.41  (0.56-0.29) | **-3.25**  **(-3.67--2.83)** | <0.001 | 0.89  (1.22-0.66) | 0.18  (0.25-0.13) | **-5.09**  **(-5.55--4.63)** | <0.001 | 40.93  (55.97-30.26) | 8.40  (11.58-6.04) | **-5.03**  **(-5.48--4.58)** | <0.001 |
| Republic of Moldova | 0.59  (0.67-0.52) | 0.31  (0.36-0.26) | **-2.25**  **(-3.26--1.23)** | <0.001 | 0.54  (0.61-0.47) | 0.27  (0.31-0.22) | **-2.46**  **(-3.49--1.42)** | <0.001 | 24.70  (27.97-21.75) | 12.17  (14.20-10.29) | **-2.46**  **(-3.47--1.44)** | <0.001 |
| Romania | 0.55  (0.66-0.45) | 0.65  (0.84-0.49) | 0.68  (-0.40-1.77) | 0.216 | 0.50  (0.61-0.41) | 0.58  (0.75-0.44) | 0.58  (-0.50-1.67) | 0.294 | 23.45  (28.40-19.27) | 26.52  (34.32-20.28) | 0.51  (-0.56-1.58) | 0.352 |
| Russian Federation | 1.23  (1.28-1.19) | 0.86  (0.94-0.78) | **-1.14**  **(-2.24--0.04)** | 0.043 | 1.09  (1.13-1.05) | 0.70  (0.76-0.63) | **-1.44**  **(-2.58--0.29)** | 0.014 | 49.83  (51.76-47.89) | 32.44  (35.33-29.34) | **-1.38**  **(-2.49--0.27)** | 0.015 |
| Rwanda | 5.17  (7.64-3.05) | 2.11  (3.18-1.31) | **-2.88**  **(-3.16--2.60)** | <0.001 | 4.80  (7.05-2.82) | 1.93  (2.92-1.19) | **-2.93**  **(-3.25--2.62)** | <0.001 | 225.66  (332.58-132.21) | 91.04  (138.28-56.09) | **-2.92**  **(-3.23--2.61)** | <0.001 |
| Saint Kitts and Nevis | 1.14  (1.31-0.98) | 0.64  (0.85-0.47) | **-1.72**  **(-2.90--0.53)** | 0.005 | 1.04  (1.20-0.90) | 0.57  (0.75-0.42) | **-1.82**  **(-2.97--0.65)** | 0.002 | 48.78  (56.19-42.16) | 26.05  (34.77-19.09) | **-1.89**  **(-3.00--0.75)** | 0.001 |
| Saint Lucia | 1.35  (1.55-1.17) | 1.10  (1.40-0.86) | **-0.72**  **(-1.42--0.02)** | 0.045 | 1.23  (1.42-1.06) | 0.98  (1.22-0.76) | **-0.81**  **(-1.52--0.09)** | 0.027 | 57.20  (65.99-49.59) | 45.83  (57.21-35.66) | **-0.82**  **(-1.49--0.14)** | 0.018 |
| Saint Vincent and the Grenadines | 0.64  (0.75-0.55) | 0.73  (0.89-0.60) | **0.39**  **(0.05-0.74)** | 0.027 | 0.58  (0.68-0.50) | 0.66  (0.80-0.54) | 0.34  (-0.01-0.69) | 0.055 | 27.14  (31.55-23.29) | 30.46  (36.85-24.98) | 0.33  (-0.01-0.66) | 0.055 |
| Samoa | 0.22  (0.33-0.14) | 0.23  (0.34-0.14) | **0.12**  **(0.08-0.17)** | <0.001 | 0.20  (0.30-0.13) | 0.20  (0.30-0.12) | -0.00  (-0.05-0.05) | 0.946 | 9.27  (14.12-6.00) | 9.31  (14.18-5.80) | 0.02  (-0.03-0.07) | 0.403 |
| San Marino | 0.34  (0.52-0.22) | 0.21  (0.36-0.10) | **-1.93**  **(-2.28--1.58)** | <0.001 | 0.26  (0.39-0.17) | 0.14  (0.23-0.07) | **-2.34**  **(-2.58--2.11)** | <0.001 | 12.18  (18.46-7.74) | 6.39  (10.94-3.10) | **-2.32**  **(-2.54--2.09)** | <0.001 |
| Sao Tome and Principe | 0.42  (0.63-0.27) | 0.79  (1.29-0.47) | **2.03**  **(1.85-2.20)** | <0.001 | 0.39  (0.58-0.25) | 0.72  (1.17-0.43) | **1.99**  **(1.82-2.17)** | <0.001 | 18.23  (27.50-11.58) | 33.41  (54.73-20.00) | **1.96**  **(1.78-2.13)** | <0.001 |
| Saudi Arabia | 0.37  (0.56-0.23) | 0.36  (0.56-0.23) | -0.05  (-0.25-0.16) | 0.636 | 0.33  (0.51-0.21) | 0.30  (0.46-0.19) | **-0.39**  **(-0.58--0.20)** | <0.001 | 15.78  (24.14-9.89) | 14.22  (21.87-8.98) | **-0.33**  **(-0.53--0.13)** | 0.001 |
| Senegal | 0.58  (0.83-0.39) | 0.91  (1.38-0.57) | **1.47**  **(1.05-1.89)** | <0.001 | 0.54  (0.78-0.36) | 0.83  (1.28-0.52) | **1.45**  **(1.04-1.87)** | <0.001 | 25.09  (36.28-16.83) | 38.94  (59.81-24.22) | **1.46**  **(1.04-1.88)** | <0.001 |
| Serbia | 0.56  (0.83-0.38) | 0.42  (0.62-0.28) | **-0.79**  **(-1.12--0.47)** | <0.001 | 0.51  (0.75-0.34) | 0.36  (0.52-0.24) | **-1.02**  **(-1.34--0.69)** | <0.001 | 23.77  (35.13-15.99) | 16.32  (23.79-10.98) | **-1.07**  **(-1.39--0.74)** | <0.001 |
| Seychelles | 1.68  (2.38-1.17) | 1.44  (2.08-0.97) | -0.47  (-1.07-0.13) | 0.123 | 1.5  (2.13-1.04) | 1.21  (1.75-0.81) | **-0.68**  **(-1.27--0.09)** | 0.025 | 71.09  (100.73-49.09) | 57.03  (82.80-38.31) | **-0.69**  **(-1.23--0.14)** | 0.014 |
| Sierra Leone | 0.50  (0.74-0.31) | 0.91  (1.45-0.55) | **1.95**  **(1.72-2.19)** | <0.001 | 0.46  (0.70-0.29) | 0.84  (1.33-0.50) | **1.95**  **(1.72-2.19)** | <0.001 | 21.60  (32.60-13.46) | 39.17  (62.52-23.55) | **1.96**  **(1.72-2.20)** | <0.001 |
| Singapore | 0.52  (0.63-0.42) | 0.32  (0.39-0.25) | **-1.48**  **(-2.48--0.48)** | 0.004 | 0.38  (0.46-0.31) | 0.14  (0.17-0.11) | **-3.08**  **(-3.65--2.50)** | <0.001 | 17.54  (21.55-14.35) | 6.64  (8.14-5.36) | **-3.06**  **(-3.62--2.50)** | <0.001 |
| Slovakia | 1.51  (2.17-1.03) | 0.77  (1.15-0.50) | **-2.27**  **(-3.23--1.31)** | <0.001 | 1.33  (1.90-0.91) | 0.64  (0.97-0.41) | **-2.43**  **(-3.40--1.46)** | <0.001 | 60.78  (86.85-41.50) | 29.50  (44.83-19.06) | **-2.39**  **(-3.24--1.55)** | <0.001 |
| Slovenia | 0.90  (1.10-0.74) | 0.48  (0.63-0.36) | **-2.23**  **(-3.04--1.41)** | <0.001 | 0.79  (0.96-0.64) | 0.37  (0.49-0.28) | **-2.64**  **(-3.45--1.83)** | <0.001 | 36.49  (44.41-29.72) | 17.34  (22.61-12.97) | **-2.62**  **(-3.42--1.81)** | <0.001 |
| Solomon Islands | 0.70  (1.14-0.36) | 0.71  (1.16-0.41) | -0.00  (-0.30-0.30) | 0.987 | 0.64  (1.03-0.32) | 0.64  (1.03-0.38) | -0.05  (-0.35-0.25) | 0.736 | 31.04  (49.59-15.30) | 31.22  (49.75-18.37) | -0.03  (-0.33-0.27) | 0.853 |
| Somalia | 4.71  (7.65-2.78) | 3.05  (4.85-1.82) | **-1.38**  **(-1.50--1.25)** | <0.001 | 4.39  (7.15-2.57) | 2.83  (4.53-1.69) | **-1.38**  **(-1.51--1.25)** | <0.001 | 204.55  (333.04-120.23) | 132.71  (212.92-79.47) | **-1.36**  **(-1.49--1.23)** | <0.001 |
| South Africa | 4.91  5.75-4.18) | 2.49  (2.94-2.11) | **-2.23**  **(-2.83--1.63)** | <0.001 | 4.46  (5.24-3.80) | 2.23  (2.64-1.90) | **-2.28**  **(-2.89--1.67)** | <0.001 | 212.47  (249.87-180.31) | 104.70  (123.98-88.86) | **-2.28**  **(-2.90--1.67)** | <0.001 |
| South Sudan | 3.48  (5.49-2.13) | 3.02  (4.55-1.85) | **-0.41**  **(-0.64--0.18)** | <0.001 | 3.22  (5.04-1.97) | 2.78  (4.18-1.69) | **-0.43**  **(-0.66--0.20)** | <0.001 | 150.44  (235.82-91.84) | 130.53  (196.88-79.47) | **-0.41**  **(-0.65--0.18)** | 0.001 |
| Spain | 1.39  (1.68-1.14) | 0.58  (0.73-0.46) | **-2.81**  **(-3.41--2.21)** | <0.001 | 1.11  (1.33-0.91) | 0.36  (0.45-0.29) | **-3.56**  **(-4.12--3.00)** | <0.001 | 51.56  (62.17-42.20) | 17.28  (21.32-13.73) | **-3.51**  **(-4.05--2.96)** | <0.001 |
| Sri Lanka | 1.45  (2.01-1.02) | 0.99  (1.52-0.56) | **-1.20**  **(-2.26--0.13)** | 0.028 | 1.29  (1.78-0.91) | 0.77  (1.17-0.44) | **-1.62**  **(-2.65--0.58)** | 0.002 | 62.23  (86.02-43.66) | 36.63  (55.87-20.78) | **-1.67**  **(-2.70--0.63)** | 0.002 |
| Sudan | 1.19  (1.83-0.60) | 0.88  (1.47-0.43) | **-0.96**  **(-1.02--0.90)** | <0.001 | 1.10  (1.70-0.54) | 0.79  (1.30-0.39) | **-1.05**  **(-1.11--0.99)** | <0.001 | 52.22  (81.37-25.90) | 37.88  (62.69-18.43) | **-1.03**  **(-1.09--0.96)** | <0.001 |
| Suriname | 0.40  (0.55-0.28) | 0.41  (0.62-0.26) | 0.36  (-1.09-1.83) | 0.630 | 0.37  (0.51-0.26) | 0.37  (0.56-0.24) | 0.08  (-1.73-1.93) | 0.929 | 17.40  (23.89-12.09) | 17.52  (26.33-11.37) | 0.29  (-1.14-1.74) | 0.693 |
| Sweden | 0.41  (0.47-0.35) | 0.35  (0.42-0.28) | -0.63  (-1.49-0.23) | 0.152 | 0.30  (0.35-0.26) | 0.23  (0.29-0.19) | -0.88  (-1.90-0.14) | 0.091 | 14.14  (16.38-12.20) | 10.97  (13.50-8.91) | -0.89  (-1.87-0.10) | 0.076 |
| Switzerland | 1.07  (1.29-0.89) | 0.58  (0.73-0.46) | **-2.32**  **(-3.29--1.33)** | <0.001 | 0.82  (0.98-0.67) | 0.35  (0.44-0.28) | **-3.03**  **(-4.04--2.01)** | <0.001 | 38.00  (45.82-31.42) | 16.64  (20.67-12.99) | **-3.00**  **(-4.00--1.99)** | <0.001 |
| Syrian Arab Republic | 0.22  (0.30-0.15) | 0.17  (0.23-0.11) | **-0.96**  **(-1.20--0.72)** | <0.001 | 0.20  (0.27-0.13) | 0.14  (0.20-0.10) | **-1.20**  **(-1.45--0.95)** | <0.001 | 9.55  (12.84-6.49) | 6.58  (9.35-4.57) | **-1.22**  **(-1.45--0.98)** | <0.001 |
| Taiwan (Province of China) | 2.72  (3.07-2.38) | 4.27  (4.95-3.62) | **1.25**  **(0.73-1.76)** | <0.001 | 2.20  (2.48-1.93) | 2.74  (3.15-2.37) | 0.49  (-0.02-1.02) | 0.062 | 102.78  (115.91-90.00) | 126.58  (145.50-109.12) | 0.44  (-0.08-0.95) | 0.096 |
| Tajikistan | 2.48  (3.16-1.88) | 1.01  (1.46-0.69) | **-2.84**  **(-3.03--2.66)** | <0.001 | 2.29  (2.93-1.74) | 0.93  (1.34-0.64) | **-2.86**  **(-3.05--2.67)** | <0.001 | 108.97  (139.35-82.62) | 44.97  (64.97-30.90) | **-2.82**  **(-3.00--2.65)** | <0.001 |
| Thailand | 1.22  (1.84-0.80) | 2.03  (3.03-1.30) | **1.81**  **(1.37-2.24)** | <0.001 | 1.08  (1.61-0.71) | 1.59  (2.35-1.02) | **1.39**  **(0.94-1.83)** | <0.001 | 50.55  (75.34-33.22) | 75.65  (111.99-48.61) | **1.45**  **(1.00-1.90)** | <0.001 |
| Timor-Leste | 0.61  (0.93-0.36) | 0.46  (0.68-0.29) | **-0.94**  **(-1.38--0.50)** | <0.001 | 0.56  (0.86-0.33) | 0.41  (0.61-0.26) | **-1.02**  **(-1.46--0.58)** | <0.001 | 26.87  (41.67-16.01) | 19.67  (29.44-12.34) | **-1.02**  **(-1.49--0.55)** | <0.001 |
| Togo | 0.57  (0.83-0.38) | 1.16  (1.83-0.69) | **2.37**  **(2.16-2.58)** | <0.001 | 0.53  (0.77-0.35) | 1.07  (1.68-0.63) | **2.36**  **(2.15-2.57)** | <0.001 | 24.82  (35.89-16.33) | 49.64  (78.11-29.06) | **2.34**  **(2.13-2.55)** | <0.001 |
| Tokelau | 0.4  (0.70-0.24) | 0.42  (0.67-0.25) | 0.02  (-0.32-0.35) | 0.926 | 0.38  (0.63-0.22) | 0.37  (0.58-0.22) | -0.11  (-0.33-0.11) | 0.323 | 18.70  (30.53-10.64) | 18.04  (28.41-10.77) | -0.07  (-0.27-0.13) | 0.500 |
| Tonga | 0.39  (0.56-0.26) | 0.41  (0.65-0.24) | **0.13**  **(0.00-0.26)** | 0.047 | 0.34  (0.50-0.23) | 0.35  (0.55-0.21) | 0.04  (-0.07-0.15) | 0.475 | 16.45  (23.76-10.98) | 16.92  (26.64-10.14) | 0.06  (-0.05-0.18) | 0.279 |
| Trinidad and Tobago | 0.49  (0.56-0.43) | 0.52  (0.70-0.37) | 0.57  (-0.37-1.51) | 0.235 | 0.45  (0.51-0.39) | 0.46  (0.63-0.33) | 0.46  (-0.48-1.41) | 0.334 | 20.80  (23.59-18.18) | 21.57  (29.36-15.36) | 0.50  (-0.41-1.42) | 0.281 |
| Tunisia | 0.11  (0.15-0.08) | 0.11  (0.18-0.07) | 0.11  (-0.01-0.22) | 0.062 | 0.10  (0.14-0.07) | 0.09  (0.14-0.06) | **-0.16**  **(-0.27--0.06)** | 0.002 | 4.60  (6.41-3.21) | 4.42  (6.82-2.71) | **-0.16**  **(-0.26--0.06)** | 0.002 |
| Turkey | 0.75  (1.05-0.48) | 0.37  (0.52-0.24) | **-2.32**  **(-2.71--1.93)** | <0.001 | 0.68  (0.95-0.43) | 0.30  (0.42-0.20) | **-2.67**  **(-3.07--2.26)** | <0.001 | 32.74  (46.33-20.89) | 14.26  (20.17-9.42) | **-2.68**  **(-3.10--2.26)** | <0.001 |
| Turkmenistan | 5.54  (6.03-5.04) | 1.78  (2.33-1.34) | **-3.79**  **(-4.64--2.94)** | <0.001 | 5.11  (5.55-4.65) | 1.62  (2.14-1.23) | **-3.83**  **(-4.70--2.96)** | <0.001 | 243.28  (264.76-221.07) | 77.87  (102.67-59.02) | **-3.80**  **(-4.63--2.96)** | <0.001 |
| Tuvalu | 0.62  (0.93-0.39) | 0.58  (0.91-0.35) | **-0.19**  **(-0.26--0.12)** | <0.001 | 0.57  (0.85-0.36) | 0.52  (0.81-0.31) | **-0.28**  **(-0.36--0.20)** | <0.001 | 27.42  (41.26-17.29) | 25.08  (39.48-15.05) | **-0.28**  **(-0.35--0.21)** | <0.001 |
| Uganda | 2.75  (4.03-1.82) | 3.06  (4.62-1.88) | **0.30**  **(0.13-0.47)** | 0.001 | 2.55  (3.73-1.68) | 2.81  (4.26-1.73) | **0.27**  **(0.10-0.45)** | 0.002 | 119.04  (174.77-78.33) | 132.15  (200.76-81.42) | **0.30**  **(0.13-0.47)** | 0.001 |
| Ukraine | 1.18  (1.42-0.97) | 0.81  (1.21-0.51) | **-1.29**  **(-2.42--0.15)** | 0.027 | 0.97  (1.17-0.80) | 0.63  (0.94-0.39) | **-1.48**  **(-2.58--0.37)** | 0.009 | 44.36  (53.61-36.50) | 29.27  (43.53-18.16) | **-1.44**  **(-2.53--0.33)** | 0.011 |
| United Arab Emirates | 0.34  (0.52-0.20) | 0.20  (0.29-0.12) | **-1.62**  **(-1.89--1.35)** | <0.001 | 0.30  (0.47-0.18) | 0.17  (0.25-0.11) | **-1.77**  **(-2.04--1.50)** | <0.001 | 14.41  (22.44-8.71) | 8.42  (12.50-5.34) | **-1.62**  **(-1.87--1.38)** | <0.001 |
| United Kingdom | 1.13  (1.15-1.10) | 1.17  (1.22-1.13) | 0.14  (-0.44-0.72) | 0.643 | 0.91  (0.94-0.89) | 0.83  (0.86-0.80) | -0.33  (-0.92-0.26) | 0.277 | 42.85  (43.96-41.77) | 39.31  (40.84-37.93) | -0.29  (-0.90-0.32) | 0.350 |
| United Republic of Tanzania | 3.45  (5.05-2.23) | 2.21  (3.32-1.40) | **-1.42**  **(-1.55--1.29)** | <0.001 | 3.18  (4.68-2.04) | 2.02  (3.03-1.28) | **-1.45**  **(-1.58--1.32)** | <0.001 | 149.84  (221.16-96.04) | 95.31  (143.50-60.57) | **-1.44**  **(-1.56--1.32)** | <0.001 |
| United States of America | 0.90  (0.92-0.88) | 0.81  (0.84-0.77) | -0.31  (-0.90-0.28) | 0.306 | 0.66  (0.67-0.64) | 0.53  (0.55-0.50) | **-0.65**  **(-1.19--0.12)** | 0.017 | 30.70  (31.46-29.85) | 25.10  (26.17-24.09) | **-0.57**  **(-1.13--0.00)** | 0.048 |
| United States Virgin Islands | 0.79  (1.15-0.54) | 0.82  (1.28-0.51) | 0.22  (-0.19-0.63) | 0.289 | 0.71  (1.03-0.49) | 0.71  (1.09-0.44) | **0.42**  **(0.19-0.65)** | <0.001 | 33.29  (48.25-22.83) | 33.73  (51.57-20.94) | 0.17  (-0.22-0.57) | 0.398 |
| Uruguay | 1.25  (1.52-1.02) | 0.85  (1.04-0.68) | -1.01  (-2.24-0.25) | 0.115 | 1.10  (1.33-0.90) | 0.69  (0.85-0.56) | -1.23  (-2.48-0.03) | 0.055 | 51.55  (62.23-41.98) | 32.98  (40.72-26.65) | -1.19  (-2.39-0.03) | 0.056 |
| Uzbekistan | 2.87  (3.39-2.38) | 0.85  (1.11-0.64) | **-3.72**  **(-5.46--1.94)** | <0.001 | 2.65  (3.12-2.20) | 0.77  (1.01-0.59) | **-3.76**  **(-5.52--1.98)** | <0.001 | 125.53  (148.14-104.41) | 37.75  (49.14-28.64) | **-3.72**  **(-5.77--1.63)** | 0.001 |
| Vanuatu | 0.61  (1.01-0.34) | 0.59  (0.92-0.35) | -0.12  (-0.25-0.02) | 0.088 | 0.55  (0.92-0.31) | 0.53  (0.83-0.32) | **-0.15**  **(-0.28--0.02)** | 0.028 | 26.68  (44.33-14.91) | 25.85  (40.60-15.40) | -0.13  (-0.27-0.01) | 0.073 |
| Bolivarian Republic of Venezuela | 0.42  (0.46-0.37) | 0.33  (0.46-0.23) | -0.61  (-1.95-0.76) | 0.383 | 0.38  (0.42-0.34) | 0.29  (0.40-0.20) | -0.72  (-2.09-0.67) | 0.309 | 18.06  (20.11-16.22) | 14.28  (19.68-9.94) | -0.63  (-1.99-0.76) | 0.373 |
| Viet Nam | 0.40  (0.62-0.25) | 0.51  (0.82-0.31) | **0.79**  **(0.56-1.01)** | <0.001 | 0.36  (0.56-0.23) | 0.42  (0.67-0.25) | **0.48**  **(0.26-0.70)** | <0.001 | 16.98  (25.95-10.62) | 19.33  (30.84-11.71) | **0.43**  **(0.30-0.56)** | <0.001 |
| Yemen | 1.13  (1.82-0.55) | 0.76  (1.25-0.35) | **-1.28**  **(-1.54--1.02)** | <0.001 | 1.04  (1.69-0.51) | 0.69  (1.13-0.31) | **-1.34**  **(-1.62--1.07)** | <0.001 | 48.82  (79.20-23.85) | 32.38  (53.34-14.67) | **-1.32**  **(-1.60--1.03)** | <0.001 |
| Zambia | 4.41  (6.24-2.94) | 3.65  (6.46-2.04) | **-0.60**  **(-0.75--0.44)** | <0.001 | 4.09  (5.81-2.71) | 3.35  (6.00-1.86) | **-0.62**  **(-0.77--0.46)** | <0.001 | 193.01  (274.61-127.46) | 157.69  (285.22-87.27) | **-0.63**  **(-0.78--0.48)** | <0.001 |
| Zimbabwe | 2.29  (3.24-1.57) | 4.13  (6.28-2.52) | **1.96**  **(1.24-2.69)** | <0.001 | 2.10  (2.95-1.44) | 3.79  (5.67-2.31) | **1.96**  **(1.21-2.72)** | <0.001 | 97.99  (137.68-66.86) | 178.33  (266.69-108.89) | **1.99**  **(1.25-2.74)** | <0.001 |

Estimates are for individuals aged 20–49 years. Values in parentheses are 95% uncertainty intervals (UI) or confidence intervals (CI). Rates are reported per 100000 population. Bold within the table means the average annual percent change (AAPC) is statistically significant (*P*≤0.05). UI=uncertainty interval. CI=confidence interval. AAPC=average annual percent change. DALY=disability-adjusted life year. *P*=*P*-value for the significant test of AAPC.

**Supplementary Table 5. Age-Specific AAPC of the Global Burden of Early-Onset Esophageal Cancer from 1990 to 2021, by SDI Categories**

| **Age** | **Location** | **Global** | | **High SDI** | | **High-middle SDI** | | **Middle SDI** | | **Low-middle SDI** | | **Low SDI** | |
| --- | --- | --- | --- | --- | --- | --- | --- | --- | --- | --- | --- | --- | --- |
|  | **Sex** | **Men** | **women** | **Men** | **women** | **Men** | **women** | **Men** | **women** | **Men** | **women** | **Men** | **women** |
| **Age-specific incidence rate** | | | | | | | | | | | | | |
| **20 to 24** | Rates in 1990  (95% UI) | 0.14  (0.12-0.16) | 0.10  (0.07-0.11) | 0.06  (0.06-0.06) | 0.02  (0.02-0.03) | 0.21  (0.17-0.26) | 0.08  (0.05-0.11) | 0.19  (0.16-0.23) | 0.11  (0.07-0.13) | 0.07  (0.06-0.08) | 0.12  (0.09-0.15) | 0.07  (0.05-0.08) | 0.14  (0.09-0.17) |
|  | Rates in 2021  (95% UI) | 0.08  (0.08-0.10) | 0.07  (0.05-0.09) | 0.06  (0.06-0.07) | 0.02  (0.02-0.03) | 0.13  (0.10-0.17) | 0.04  (0.03-0.05) | 0.10  (0.08-0.12) | 0.06  (0.04-0.07) | 0.07  (0.06-0.08) | 0.08  (0.06-0.14) | 0.07  (0.06-0.08) | 0.10  (0.07-0.13) |
|  | AAPC, %  (95% CI) | **-1.70**  **(-2.04--1.36)** | **-1.17**  **(-1.35--0.98)** | 0.02  (-0.49-0.54) | -0.02  (-0.47-0.44) | **-1.64**  **(-2.34--0.95)** | **-2.28**  **(-2.56--2.00)** | **-2.10**  **(-2.47--1.73)** | **-2.01**  **(-2.22--1.81)** | 0.01  (-0.37-0.39) | **-1.22**  **(-1.60--0.85)** | **0.34**  **(-0.12-0.79)** | **-1.00**  **(-1.21--0.80)** |
|  | *P* | <0.001 | <0.001 | 0.927 | 0.942 | <0.001 | <0.001 | <0.001 | <0.001 | 0.977 | <0.001 | 0.144 | <0.001 |
| **25 to 29** | Rates in 1990  (95% UI) | 0.25  (0.22-0.28) | 0.18  (0.13-0.20) | 0.12  (0.12-0.13) | 0.05  (0.05-0.05) | 0.35  (0.28-0.42) | 0.13  (0.09-0.16) | 0.35  (0.29-0.42) | 0.19  (0.13-0.23) | 0.14  (0.12-0.17) | 0.25  (0.19-0.30) | 0.13  (0.11-0.16) | 0.28  (0.19-0.35) |
|  | Rates in 2021  (95% UI) | 0.16  (0.14-0.18) | 0.13  (0.10-0.16) | 0.14  (0.13-0.15) | 0.05  (0.05-0.06) | 0.22  (0.17-0.28) | 0.07  (0.05-0.08) | 0.19  (0.16-0.22) | 0.11  (0.08-0.13) | 0.12  (0.11-0.14) | 0.17  (0.13-0.25) | 0.12  (0.10-0.14) | 0.20  (0.14-0.27) |
|  | AAPC, %  (95% CI) | **-1.47**  **(-1.83--1.11)** | **-1.04**  **(-1.31--0.77)** | 0.47  (-0.07-1.02) | 0.09  (-0.26-0.44) | **-1.56**  **(-1.91--1.21)** | **-2.08**  **(-2.57--1.58)** | **-2.04**  **(-2.57--1.51)** | **-1.87**  **(-2.16--1.58)** | **-0.48**  **(-0.85--0.12)** | **-1.21**  **(-1.61--0.81)** | **-0.48**  **(-0.77--0.19)** | **-1.14**  **(-1.32--0.96)** |
|  | *P* | <0.001 | <0.001 | 0.086 | 0.611 | <0.001 | <0.001 | <0.001 | <0.001 | 0.009 | <0.001 | 0.001 | <0.001 |
| **30 to 34** | Rates in 1990  (95% UI) | 0.74  (0.66-0.84) | 0.30  (0.21-0.33) | 0.33  (0.32-0.35) | 0.09  (0.09-0.10) | 1.10  (0.90-1.33) | 0.24  (0.16-0.29) | 1.09  (0.92-1.29) | 0.37  (0.25-0.44) | 0.32  (0.28-0.38) | 0.37  (0.28-0.46) | 0.36  (0.29-0.43) | 0.44  (0.28-0.54) |
|  | Rates in 2021  (95% UI) | 0.48  (0.43-0.54) | 0.21  (0.17-0.26) | 0.34  (0.32-0.36) | 0.10  (0.09-0.11) | 0.74  (0.58-0.92) | 0.13  (0.10-0.17) | 0.58  (0.49-0.68) | 0.19  (0.15-0.23) | 0.31  (0.28-0.35) | 0.27  (0.21-0.39) | 0.34  (0.28-0.40) | 0.33  (0.24-0.45) |
|  | AAPC, %  (95% CI) | **-1.26**  **(-1.81--0.70)** | **-1.14**  **(-1.28--1.00)** | 0.17  (-0.49-0.83) | 0.25  (-0.14-0.63) | **-1.19**  **(-1.84--0.54)** | **-1.74**  **(-2.46--1.00)** | **-1.85**  **(-2.60--1.10)** | **-2.04**  **(-2.38--1.69)** | -0.15  (-0.33-0.04) | **-1.07**  **(-1.41--0.72)** | **-0.20**  **(-0.37--0.03)** | **-0.99**  **(-1.25--0.73)** |
|  | *P* | <0.001 | <0.001 | 0.622 | 0.208 | <0.001 | <0.001 | <0.001 | <0.001 | 0.116 | <0.001 | 0.019 | <0.001 |
| **35 to 39** | Rates in 1990  (95% UI) | 2.54  (2.19-2.95) | 0.73  (0.50-0.85) | 1.07  (1.02-1.12) | 0.25  (0.24-0.26) | 3.85  (3.19-4.72) | 0.68  (0.41-0.85) | 3.81  (3.09-4.75) | 0.96  (0.57-1.17) | 0.81  (0.70-0.95) | 0.80  (0.61-0.98) | 1.03  (0.85-1.24) | 1.05  (0.66-1.29) |
|  | Rates in 2021  (95% UI) | 1.24  (1.10-1.42) | 0.45  (0.37-0.54) | 0.88  (0.83-0.93) | 0.24  (0.23-0.26) | 1.94  (1.52-2.46) | 0.31  (0.24-0.40) | 1.46  (1.22-1.76) | 0.41  (0.33-0.48) | 0.77  (0.68-0.89) | 0.60  (0.49-0.81) | 0.94  (0.76-1.15) | 0.74  (0.53-1.01) |
|  | AAPC, %  (95% CI) | **-2.33**  **(-2.87--1.79)** | **-1.61**  **(-1.86--1.35)** | **-0.48**  **(-0.80--0.15)** | -0.02  (-0.68-0.65) | **-2.31**  **(-2.88--1.74)** | **-2.49**  **(-2.91--2.06)** | **-3.26**  **(-4.01--2.51)** | **-2.76**  **(-3.04--2.48)** | -0.14  (-0.63-0.35) | **-0.94**  **(-1.31--0.57)** | **-0.29**  **(-0.37--0.21)** | **-1.15**  **(-1.42--0.89)** |
|  | *P* | <0.001 | <0.001 | 0.004 | 0.964 | <0.001 | <0.001 | <0.001 | <0.001 | 0.573 | <0.001 | <0.001 | <0.001 |
| **40 to 44** | Rates in 1990  (95% UI) | 6.80  (5.85-7.89) | 1.82  (1.16-2.12) | 3.29  (3.14-3.48) | 0.59  (0.56-0.62) | 10.53  (8.55-12.88) | 1.79  (0.96-2.32) | 10.33  (8.27-12.81) | 2.61  (1.38-3.29) | 2.08  (1.81-2.39) | 1.81  (1.49-2.35) | 3.05  (2.50-3.75) | 2.43  (1.61-2.95) |
|  | Rates in 2021  (95% UI) | 3.36  (2.92-3.86) | 1.00  (0.85-1.19) | 2.38  (2.25-2.53) | 0.55  (0.52-0.61) | 5.50  (4.27-7.18) | 0.70  (0.56-0.87) | 3.70  (3.02-4.43) | 0.95  (0.77-1.13) | 2.06  (1.82-2.36) | 1.30  (1.06-1.74) | 2.71  (2.20-3.36) | 1.64  (1.19-2.18) |
|  | AAPC, %  (95% CI) | **-2.26**  **(-2.48--2.04)** | **-1.92**  **(-2.12--1.72)** | **-1.04**  **(-1.26--0.82)** | -0.19  (-0.60-0.23) | **-2.05**  **(-2.44--1.67)** | **-3.00**  **(-3.37--2.62)** | **-3.29**  **(-3.62--2.95)** | **-3.29**  **(-3.63--2.96)** | 0.01  (-0.21-0.24) | **-1.11**  **(-1.43--0.78)** | **-0.38**  **(-0.61--0.14)** | **-1.26**  **(-1.40--1.12)** |
|  | *P* | <0.001 | <0.001 | <0.001 | 0.374 | <0.001 | <0.001 | <0.001 | <0.001 | 0.911 | <0.001 | 0.002 | <0.001 |
| **45 to 49** | Rates in 1990  (95% UI) | 13.30  (11.76-15.26) | 3.72  (2.50-4.32) | 8.57  (8.15-8.99) | 1.44  (1.38-1.50) | 21.30  (17.66-25.15) | 3.67  (1.95-4.63) | 18.00  (14.55-21.99) | 4.98  (2.68-6.28) | 4.74  (4.18-5.46) | 3.85  (3.14-5.08) | 7.09  (5.89-8.43) | 5.33  (3.60-6.48) |
|  | Rates in 2021  (95% UI) | 7.82  (6.60-9.16) | 2.13  (1.79-2.52) | 5.68  (5.33-6.02) | 1.25  (1.19-1.34) | 12.53  (9.68-16.16) | 1.52  (1.19-1.97) | 8.41  (6.66-10.58) | 2.10  (1.64-2.54) | 4.50  (3.95-5.12) | 2.72  (2.27-3.69) | 6.18  (5.07-7.54) | 3.73  (2.56-4.79) |
|  | AAPC, %  (95% CI) | **-1.74**  **(-2.01--1.48)** | **-1.82**  **(-2.12--1.52)** | **-1.33**  **(-1.57--1.09)** | **-0.49**  **(-0.77--0.21)** | **-1.80**  **(-2.29--1.30)** | **-2.87**  **(-3.39--2.35)** | **-2.52**  **(-2.92--2.12)** | **-2.75**  **(-3.02--2.48)** | -0.14  (-0.33-0.04) | **-1.08**  **(-1.48--0.67)** | **-0.44**  **(-0.51--0.38)** | **-1.14**  **(-1.23--1.05)** |
|  | *P* | <0.001 | <0.001 | 0.001 | <0.001 | <0.001 | <0.001 | <0.001 | <0.001 | 0.128 | <0.001 | <0.001 | <0.001 |
| **Age-specific mortality rate** | | | | | | | | | | | | | |
| **20 to 24** | Rates in 1990  (95% UI) | 0.14  (0.12-0.16) | 0.09  (0.06-0.11) | 0.05  (0.05-0.06) | 0.02  (0.02-0.02) | 0.21  (0.17-0.25) | 0.08  (0.05-0.10) | 0.19  (0.16-0.23) | 0.10  (0.07-0.12) | 0.07  (0.06-0.08) | 0.12  (0.10-0.15) | 0.07  (0.05-0.08) | 0.14  (0.09-0.18) |
|  | Rates in 2021  (95% UI) | 0.08  (0.07-0.09) | 0.06  (0.05-0.08) | 0.05  (0.04-0.05) | 0.01  (0.01-0.02) | 0.10  (0.08-0.13) | 0.03  (0.02-0.03) | 0.09  (0.08-0.10) | 0.05  (0.04-0.06) | 0.07  (0.06-0.08) | 0.08  (0.06-0.14) | 0.07  (0.06-0.09) | 0.10  (0.07-0.14) |
|  | AAPC, %  (95% CI) | **-2.01**  **(-2.33--1.69)** | **-1.34**  **(-1.51--1.17)** | **-0.57**  **(-1.12--0.01)** | **-0.96**  **(-1.40--0.51)** | **-2.41**  **(-3.07--1.74)** | **-3.34**  **(-3.80--2.88)** | **-2.50**  **(-2.84--2.16)** | **-2.49**  **(-2.66--2.32)** | -0.05  (-0.43-0.34) | **-1.30**  **(-1.68--0.93)** | **0.31**  **(-0.14-0.77)** | -1.04  (-1.25--0.83) |
|  | *P* | <0.001 | <0.001 | 0.045 | <0.001 | <0.001 | <0.001 | <0.001 | <0.001 | 0.803 | <0.001 | <0.001 | 0.175 |
| **25 to 29** | Rates in 1990  (95% UI) | 0.23  (0.20-0.26) | 0.16  (0.11-0.18) | 0.10  (0.09-0.10) | 0.04  (0.03-0.04) | 0.31  (0.26-0.38) | 0.11  (0.08-0.14) | 0.32  (0.27-0.38) | 0.17  (0.11-0.20) | 0.13  (0.11-0.16) | 0.23  (0.18-0.28) | 0.12  (0.10-0.15) | 0.26  (0.17-0.32) |
|  | Rates in 2021  (95% UI) | 0.13  (0.12-0.14) | 0.11  (0.09-0.14) | 0.09  (0.09-0.10) | 0.03  (0.03-0.03) | 0.16  (0.13-0.20) | 0.04  (0.03-0.05) | 0.15  (0.14-0.18) | 0.08  (0.06-0.10) | 0.11  (0.10-0.13) | 0.15  (0.12-0.23) | 0.11  (0.09-0.13) | 0.18  (0.13-0.25) |
|  | AAPC, %  (95% CI) | **-1.87**  **(-2.27--1.46)** | **-1.23**  **(-1.51--0.96)** | -0.08  (-0.62-0.46) | **-0.61**  **(-1.03--0.20)** | **-2.40**  **(-2.79--2.00)** | **-3.09**  **(-3.60--2.58)** | **-2.38**  **(-2.72--2.03)** | **-2.27**  **(-2.59--1.95)** | **-0.54**  **(-0.90--0.18)** | **-1.28**  **(-1.68--0.89)** | **-0.51**  **(-0.80--0.22)** | **-1.18**  **(-1.36--1.00)** |
|  | *P* | <0.001 | <0.001 | 0.767 | 0.004 | <0.001 | <0.001 | <0.001 | <0.001 | 0.004 | <0.001 | 0.001 | <0.001 |
| **30 to 34** | Rates in 1990  (95% UI) | 0.66  (0.59-0.75) | 0.26  (0.18-0.29) | 0.26  (0.25-0.28) | 0.07  (0.06-0.07) | 0.97  (0.80-1.18) | 0.20  (0.14-0.25) | 0.98  (0.83-1.16) | 0.31  (0.21-0.37) | 0.30  (0.26-0.35) | 0.34  (0.26-0.42) | 0.33  (0.27-0.40) | 0.41  (0.26-0.50) |
|  | Rates in 2021  (95% UI) | 0.38  (0.34-0.42) | 0.16  (0.14-0.21) | 0.23  (0.22-0.24) | 0.05  (0.05-0.06) | 0.51  (0.40-0.63) | 0.08  (0.06-0.10) | 0.45  (0.39-0.53) | 0.14  (0.11-0.16) | 0.28  (0.25-0.32) | 0.24  (0.19-0.35) | 0.31  (0.26-0.38) | 0.30  (0.21-0.41) |
|  | AAPC, %  (95% CI) | **-1.69**  **(-2.20--1.18)** | **-1.46**  **(-1.61--1.32)** | -0.40  (-0.96-0.16) | **-0.59**  **(-0.93--0.25)** | **-1.99**  **(-2.60--1.38)** | **-2.81**  **(-3.51--2.11)** | **-2.30**  **(-3.02--1.58)** | **-2.63**  **(-3.00--2.25)** | **-0.21**  **(-0.39--0.02)** | **-1.15**  **(-1.50--0.80)** | **-0.23**  **(-0.40--0.06)** | **-1.04**  **(-1.25--0.83)** |
|  | *P* | <0.001 | <0.001 | 0.164 | 0.001 | <0.001 | <0.001 | <0.001 | <0.001 | 0.031 | <0.001 | 0.008 | <0.001 |
| **35 to 39** | Rates in 1990  (95% UI) | 2.14  (1.85-2.48) | 0.60  (0.40-0.69) | 0.80  (0.76-0.85) | 0.16  (0.15-0.17) | 3.24  (2.69-3.98) | 0.54  (0.33-0.67) | 3.26  (2.64-4.08) | 0.77  (0.46-0.93) | 0.71  (0.61-0.83) | 0.69  (0.53-0.86) | 0.91  (0.75-1.09) | 2.21  (1.45-2.69) |
|  | Rates in 2021  (95% UI) | 0.92  (0.82-1.04) | 0.33  (0.28-0.41) | 0.56  (0.52-0.59) | 0.12  (0.11-0.14) | 1.29  (1.02-1.62) | 0.18  (0.15-0.23) | 1.09  (0.93-1.30) | 0.28  (0.23-0.32) | 0.67  (0.58-0.77) | 0.50  (0.41-0.69) | 0.82  (0.66-1.01) | 0.63  (0.45-0.87) |
|  | AAPC, %  (95% CI) | **-2.75**  **(-3.43--2.07)** | **-1.88**  **(-2.13--1.64)** | **-1.16**  **(-1.60--0.72)** | **-0.89**  **(-1.49--0.29)** | **-3.07**  **(-3.62--2.52)** | **-3.48**  **(-3.83--3.13)** | **-3.69**  **(-4.40--2.98)** | **-3.29**  **(-3.58--3.01)** | -0.20  (-0.68-0.29) | **-1.04**  **(-1.40--0.67)** | **-0.31**  **(-0.39--0.23)** | **-1.20**  **(-1.47--0.93)** |
|  | *P* | <0.001 | <0.001 | <0.001 | 0.004 | <0.001 | <0.001 | <0.001 | <0.001 | 0.429 | <0.001 | <0.001 | <0.001 |
| **40 t0 44** | Rates in 1990  (95% UI) | 5.97  (5.14-6.94) | 1.53  (0.98-1.77) | 2.55  (2.43-2.70) | 0.39  (0.37-0.41) | 9.26  (7.51-11.38) | 1.49  (0.81-1.93) | 9.21  (7.36-11.42) | 2.16  (1.18-2.69) | 1.91  (1.65-2.19) | 1.64  (1.34-2.14) | 2.81  (2.30-3.43) | 2.21  (1.45-2.69) |
|  | Rates in 2021  (95% UI) | 2.58  (2.25-2.95) | 0.76  (0.66-0.92) | 1.55  (1.47-1.65) | 0.28  (0.27-0.32) | 3.84  (3.00-4.98) | 0.42  (0.35-0.52) | 2.89  (2.36-3.38) | 0.67  (0.56-0.77) | 1.85  (1.63-2.11) | 1.14  (0.93-1.52) | 2.48  (2.01-3.07) | 1.47  (1.07-1.95) |
|  | AAPC, %  (95% CI) | **-2.70**  **(-2.93--2.46)** | **-2.22**  **(-2.40--2.05)** | **-1.59**  **(-1.83--1.34)** | **-0.97**  **(-1.17--0.77)** | **-2.83**  **(-3.23--2.42)** | **-4.00**  **(-4.32--3.67)** | **-3.69**  **(-4.03--3.35)** | **-3.74**  **(-4.12--3.36)** | -0.04  (-0.27-0.18) | **-1.19**  **(-1.52--0.87)** | **-0.40**  **(-0.64--0.17)** | **-1.30**  **(-1.44--1.15)** |
|  | *P* | <0.001 | <0.001 | <0.001 | <0.001 | <0.001 | <0.001 | <0.001 | <0.001 | 0.708 | <0.001 | 0.001 | <0.001 |
| **45 to 49** | Rates in 1990  (95% UI) | 11.89  (10.47-13.67) | 3.20  (2.15-3.71) | 6.72  (6.39-7.05) | 0.97  (0.93-1.02) | 19.13  (15.91-22.64) | 3.14  (1.67-3.97) | 16.46  (13.35-20.04) | 4.23  (2.35-5.28) | 4.44  (3.92-5.13) | 3.57  (2.89-4.73) | 6.68  (5.55-8.02) | 4.98  (3.37-6.06) |
|  | Rates in 2021  (95% UI) | 6.07  (5.17-7.08) | 1.62  (1.37-1.94) | 3.71  (3.49-3.94) | 0.66  (0.63-0.72) | 8.94  (7.02-11.50) | 0.94  (0.76-1.18) | 6.67  (5.35-8.29) | 1.49  (1.20-1.77) | 4.14  (3.63-4.70) | 2.45  (2.04-3.32) | 5.78  (4.73-7.03) | 3.44  (2.35-4.45) |
|  | AAPC, %  (95% CI) | **-2.19**  **(-2.47--1.91)** | **-2.21**  **(-2.48--1.94)** | **-1.90**  **(-2.15--1.65)** | **-1.19**  **(-1.44--0.94)** | **-2.50**  **(-3.04--1.97)** | **-3.93**  **(-4.40--3.46)** | **-2.98**  **(-3.39--2.57)** | **-3.33**  **(-3.66--3.00)** | **-0.20**  **(-0.39--0.02)** | **-1.17**  **(-1.57--0.77)** | **-0.47**  **(-0.54--0.41)** | **-1.18**  **(-1.27--1.09)** |
|  | *P* | <0.001 | <0.001 | <0.001 | <0.001 | <0.001 | <0.001 | <0.001 | <0.001 | 0.032 | <0.001 | <0.001 | <0.001 |
| **Age-specific DALYs rate** | | | | | | | | | | | | | |
| **20 to 24** | Rates in 1990  (95% UI) | 9.50  (8.25-10.82) | 6.32  (4.39-7.35) | 3.67  (3.50-3.86) | 1.26  (1.18-1.37) | 14.27  (11.69-17.12) | 5.33  (3.53-6.80) | 13.09  (10.96-15.59) | 6.83  (4.48-8.35) | 4.75  (4.11-5.45) | 8.37  (6.49-10.41) | 4.57  (3.67-5.52) | 9.42  (5.95-11.93) |
|  | Rates in 2021  (95% UI) | 5.19  (4.68-5.85) | 4.15  (3.23-5.72) | 3.13  (2.97-3.29) | 0.94  (0.88-1.06) | 6.80  (5.35-8.80) | 1.87  (1.40-2.33) | 5.95  (5.11-6.98) | 3.15  (2.43-3.83) | 4.62  (4.06-5.17) | 5.61  (4.18-9.24) | 4.96  (4.09-5.91) | 6.87  (4.67-9.20) |
|  | AAPC, %  (95% CI) | **-2.01**  **(-2.32--1.69)** | **-1.34**  **(-1.51--1.17)** | **-0.57**  **(-1.12--0.02)** | **-0.95**  **(-1.39--0.51)** | **-2.41**  **(-3.07--1.74)** | **-3.33**  **(-3.80--2.87)** | **-2.50**  **(-2.84--2.16)** | **-2.49**  **(-2.65--2.32)** | **-0.05**  **(-0.44-0.34)** | **-1.30**  **(-1.68--0.93)** | 0.31  (-0.14-0.77) | **-1.04**  **(-1.25--0.83)** |
|  | *P* | 0.044 | <0.001 | <0.001 | <0.001 | <0.001 | <0.001 | <0.001 | <0.001 | <0.001 | <0.001 | 0.175 | <0.001 |
| **25 to 29** | Rates in 1990  (95% UI) | 14.48  (12.82-16.28) | 9.82  (7.07-11.21) | 6.18  (5.86-6.53) | 2.26  (2.15-2.38) | 19.89  (16.24-24.03) | 7.11  (4.75-8.84) | 20.31  (17.11-23.97) | 10.58  (7.13-12.39) | 8.39  (7.22-9.93) | 14.53  (11.12-17.82) | 7.74  (6.26-9.36) | 16.72  (10.80-20.44) |
|  | Rates in 2021  (95% UI) | 8.16  (7.38-9.02) | 6.69  (5.46-8.67) | 5.98  (5.71-6.32) | 1.81  (1.69-1.99) | 9.96  (7.92-12.60) | 2.67  (2.05-3.32) | 9.72  (8.58-11.06) | 5.21  (4.04-6.34) | 7.06  (6.25-8.15) | 9.78  (7.67-14.36) | 6.82  (5.70-8.43) | 11.60  (8.23-15.66) |
|  | AAPC, %  (95% CI) | **-1.87**  **(-2.27--1.47)** | **-1.23**  **(-1.51--0.96)** | -0.08  (-0.61-0.46) | **-0.60**  **(-1.01--0.19)** | **-2.40**  **(-2.80--2.00)** | **-3.06**  **(-3.56--2.57)** | **-2.38**  **(-2.72--2.03)** | **-2.27**  **(-2.60--1.95)** | **-0.54**  **(-0.90--0.18)** | **-1.29**  **(-1.68--0.89)** | **-0.51**  **(-0.80--0.22)** | **-1.18**  **(-1.36--1.01)** |
|  | *P* | <0.001 | <0.001 | 0.775 | 0.004 | <0.001 | <0.001 | <0.001 | <0.001 | 0.003 | <0.001 | 0.001 | <0.001 |
| **30 to 34** | Rates in 1990  (95% UI) | 38.44  (34.18-43.45) | 14.88  (10.71-16.85) | 15.31  (14.58-16.17) | 3.81  (3.60-4.04) | 56.47  (46.56-68.32) | 11.74  (8.12-14.51) | 56.78  (48.31-67.11) | 18.30  (12.35-21.77) | 17.36  (15.10-20.22) | 19.81  (15.02-24.17) | 19.23  (15.87-23.41) | 23.82  (15.13-29.29) |
|  | Rates in 2021  (95% UI) | 21.92  (19.53-24.60) | 9.45  (7.89-11.97) | 13.33  (12.59-14.18) | 3.17  (2.95-3.52) | 29.89  (23.48-36.73) | 4.75  (3.71-5.92) | 26.43  (22.74-30.69) | 8.04  (6.46-9.45) | 16.34  (14.55-18.39) | 13.92  (10.95-20.39) | 18.12  (15.02-21.82) | 17.21  (12.39-23.89) |
|  | AAPC, %  (95% CI) | **-1.69**  **(-2.18--1.20)** | **-1.46**  **(-1.61--1.31)** | -0.39  (-0.95-0.16) | **-0.57**  **(-0.91--0.23)** | **-1.98**  **(-2.58--1.38)** | **-2.80**  **(-3.50--2.10)** | **-2.30**  **(-2.98--1.62)** | **-2.62**  **(-2.99--2.25)** | **-0.21**  **(-0.39--0.02)** | **-1.15**  **(-1.50--0.80)** | **-0.23**  **(-0.40--0.06)** | **-1.04**  **(-1.26--0.83)** |
|  | *P* | <0.001 | <0.001 | 0.166 | 0.001 | <0.001 | <0.001 | <0.001 | <0.001 | 0.031 | <0.001 | 0.009 | <0.001 |
| **35 to 39** | Rates in 1990  (95% UI) | 113.98  (98.24-131.95) | 31.69  (21.42-36.54) | 42.65  (40.63-45.04) | 8.47  (8.07-8.97) | 172.01  (142.81-211.33) | 28.81  (17.77-35.79) | 173.30  (140.24-216.95) | 40.84  (24.76-49.55) | 37.59  (32.57-44.09) | 36.81  (28.24-45.89) | 48.34  (39.94-57.93) | 48.88  (30.28-60.07) |
|  | Rates in 2021  (95% UI) | 48.95  (43.47-55.29) | 17.85  (15.04-21.75) | 29.66  (27.92-31.40) | 6.52  (6.07-7.36) | 68.74  (54.25-86.34) | 9.78(  12.09-7.75) | 57.89  (49.34-69.20) | 14.90  (12.20-17.33) | 35.41  (30.99-40.88) | 26.81  (21.87-36.54) | 43.62  (35.16-53.61) | 33.69  (24.16-46.35) |
|  | AAPC, %  (95% CI) | **-2.75**  **(-3.43--2.06)** | **-1.88**  **(-2.12--1.64)** | **-1.15**  **(-1.59--0.72)** | **-0.88**  **(-1.47--0.28)** | **-3.05**  **(-3.59--2.51)** | **-3.46**  **(-3.80--3.12)** | **-3.67**  **(-4.36--2.98)** | **-3.28**  **(-3.56--3.00)** | -0.19  (-0.68-0.29) | **-1.04**  **(-1.41--0.67)** | **-0.31**  **(-0.39--0.23)** | **-1.20**  **(-1.47--0.93)** |
|  | *P* | <0.001 | <0.001 | <0.001 | 0.004 | <0.001 | <0.001 | <0.001 | <0.001 | 0.434 | <0.001 | <0.001 | <0.001 |
| **40 t0 44** | Rates in 1990  (95% UI) | 287.98  (247.88-334.97) | 73.74  (47.43-85.57) | 123.43  (117.52-130.54) | 18.91  (18.06-19.86) | 446.66  (362.09-548.72) | 72.11  (39.25-93.14) | 444.52  (355.11-550.72) | 104.52  (56.98-130.23) | 91.79  (79.52-105.65) | 79.14  (64.43-103.30) | 135.36  (110.98-165.12) | 106.77  (69.97-129.64) |
|  | Rates in 2021  (95% UI) | 124.58  (108.72-142.33) | 36.82  (31.96-44.27) | 75.14  (70.93-79.95) | 13.81  (12.92-15.52) | 185.48  (144.84-240.73) | 20.44  (16.96-25.08) | 139.50  (114.19-162.98) | 32.48  (27.15-37.27) | 89.33  (78.78-101.96) | 55.09  (44.86-73.30) | 119.53  (96.68-148.14) | 71.11  (51.52-94.06) |
|  | AAPC, %  (95% CI) | **-2.70**  **(-2.93--2.46)** | **-2.22**  **(-2.40--2.05)** | **-1.58**  **(-1.82--1.34)** | **-0.95**  **(-1.15--0.75)** | **-2.81**  **(-3.20--2.42)** | **-3.99**  **(-4.30--3.67)** | **-3.69**  **(-4.03--3.35)** | **-3.74**  **(-4.12--3.35)** | -0.04  (-0.27-0.18) | **-1.19**  **(-1.52--0.87)** | **-0.40**  **(-0.62--0.18)** | **-1.30**  **(-1.44--1.16)** |
|  | *P* | <0.001 | <0.001 | <0.001 | <0.001 | <0.001 | <0.001 | <0.001 | <0.001 | 0.717 | <0.001 | <0.001 | <0.001 |
| **45 to 49** | Rates in 1990  (95% UI) | 514.28  (452.73-591.55) | 138.44  (93.14-160.14) | 291.11  (276.90-305.59) | 42.32  (40.54-44.22) | 827.46  (688.98-980.78) | 135.83  (72.21-171.71) | 711.96  (577.32-865.39) | 183.16  (101.93-229.02) | 192.24  (169.78-221.88) | 154.41  (125.47-204.89) | 289.49  (240.82-347.95) | 215.52  (145.82-262.38) |
|  | Rates in 2021  (95% UI) | 262.83  (224.08-306.67) | 70.51  (59.62-84.25) | 161.11  (151.78-171.24) | 28.94  (27.47-31.66) | 387.40  (303.76-497.80) | 40.76  (32.90-51.41) | 288.72  (231.61-358.79) | 64.81  (51.86-76.80) | 179.40  (157.61-203.69) | 106.19  (88.33-144.14) | 250.68  (205.14-305.06) | 149.13  (101.77-192.97) |
|  | AAPC, %  (95% CI) | **-2.20**  **(-2.48--1.91)** | **-2.20**  **(-2.48--1.93)** | **-1.89**  **(-2.07--1.71)** | **-1.17**  **(-1.42--0.92)** | **-2.53**  **(-3.05--2.01)** | **-3.92**  **(-4.39--3.45)** | **-2.93**  **(-3.48--2.38)** | **-3.32**  **(-3.65--2.98)** | **-0.20**  **(-0.39--0.01)** | **-1.17**  **(-1.57--0.77)** | **-0.47**  **(-0.54--0.39)** | **-1.17**  **(-1.27--1.08)** |
|  | *P* | <0.001 | <0.001 | <0.001 | <0.001 | <0.001 | <0.001 | <0.001 | <0.001 | 0.035 | <0.001 | <0.001 | <0.001 |

Estimates are for individuals aged 20-49 years. Values in parentheses are 95% uncertainty intervals (UI) or confidence intervals (CI). Bold within the table means the average annual percent change (AAPC) is statistically significant (*P*≤0.05). AAPC=average annual percent change. UI=uncertainty interval. CI=confidence interval. SDI=Socio-demographic Index. DALY=disability-adjusted life year. *P*=*P*-value for the significant test of AAPC.

**Supplementary Table 6. Frontier Analysis of SDI and ASR for Early-Onset Esophageal Cancer Across 204 Countries and Territories from 1990 to 2021**

| **Location** | **SDI** | **DALYs** | | | | **Deaths** | | | |
| --- | --- | --- | --- | --- | --- | --- | --- | --- | --- |
|  |  | **Rate of DALYs**  **(95%UI)** | **Frontier DALYs** | **Effective difference** | **Effective difference rank (Age-standardized DALYs rank)** | **Rate of Deaths**  **(95%UI)** | **Frontier Deaths** | **Effective difference** | **Effective difference rank (Age-standardized Deaths rank)** |
| Afghanistan | 0.34 | 79.98  (33.31 to 138.48) | 9.67 | 70.32 | 177 (179) | 1.68  (0.7 to 2.91) | 0.21 | 1.47 | 175 (179) |
| Albania | 0.71 | 13.11  (8.61 to 19.4) | 4.47 | 8.64 | 41 (39) | 0.28  (0.18 to 0.41) | 0.09 | 0.18 | 43 (41) |
| Algeria | 0.66 | 4.96  (3.32 to 7.01) | 4.53 | 0.43 | 3 (2) | 0.1  (0.07 to 0.15) | 0.1 | 0.01 | 3 (2) |
| American Samoa | 0.72 | 16.9  (10.05 to 25.72) | 4.45 | 12.45 | 65 (60) | 0.35  (0.21 to 0.53) | 0.09 | 0.25 | 61 (58) |
| Andorra | 0.87 | 9.56  (5.37 to 15.71) | 4.43 | 5.12 | 29 (26) | 0.2  (0.11 to 0.34) | 0.09 | 0.11 | 31 (27) |
| Angola | 0.45 | 67.15  (39.3 to 103.7) | 6.65 | 60.5 | 171 (171) | 1.43  (0.84 to 2.2) | 0.14 | 1.29 | 171 (171) |
| Antigua and Barbuda | 0.75 | 15.82  (13.47 to 18.33) | 4.4 | 11.42 | 60 (57) | 0.34  (0.29 to 0.39) | 0.09 | 0.25 | 60 (57) |
| Argentina | 0.72 | 22.12  (17.97 to 27.09) | 4.44 | 17.68 | 87 (85) | 0.47  (0.38 to 0.57) | 0.09 | 0.37 | 85 (83) |
| Armenia | 0.70 | 5.05  (4.42 to 5.74) | 4.46 | 0.59 | 5 (3) | 0.11  (0.09 to 0.12) | 0.09 | 0.01 | 5 (4) |
| Australia | 0.84 | 22.87  (18.57 to 28.34) | 4.43 | 18.44 | 90 (87) | 0.49  (0.4 to 0.6) | 0.09 | 0.39 | 90 (88) |
| Austria | 0.85 | 14.26  (11.29 to 17.81) | 4.38 | 9.88 | 51 (46) | 0.3  (0.24 to 0.38) | 0.09 | 0.21 | 53 (49) |
| Azerbaijan | 0.69 | 34.12  (25.51 to 43.73) | 4.44 | 29.68 | 138 (135) | 0.72  (0.54 to 0.92) | 0.09 | 0.63 | 138 (134) |
| Bahamas | 0.81 | 66.9  (50.31 to 88.63) | 4.39 | 62.51 | 173 (170) | 1.42  (1.06 to 1.89) | 0.09 | 1.32 | 173 (170) |
| Bahrain | 0.75 | 9.85  (6.53 to 14.66) | 4.45 | 5.39 | 31 (27) | 0.2  (0.13 to 0.3) | 0.09 | 0.11 | 29 (26) |
| Bangladesh | 0.49 | 33.28  (19.91 to 54.48) | 4.73 | 28.55 | 131 (131) | 0.7  (0.42 to 1.13) | 0.1 | 0.6 | 130 (130) |
| Barbados | 0.75 | 41.81  (30.73 to 55.66) | 4.44 | 37.37 | 157 (154) | 0.89  (0.65 to 1.19) | 0.09 | 0.8 | 157 (153) |
| Belarus | 0.78 | 28.04  (20.36 to 37.56) | 4.45 | 23.59 | 114 (111) | 0.61  (0.45 to 0.82) | 0.09 | 0.52 | 116 (113) |
| Belgium | 0.85 | 26.9  (21.5 to 33.13) | 4.44 | 22.46 | 112 (110) | 0.57  (0.46 to 0.71) | 0.09 | 0.48 | 111 (109) |
| Belize | 0.61 | 23.12  (19 to 27.71) | 4.71 | 18.41 | 89 (89) | 0.49  (0.4 to 0.59) | 0.1 | 0.4 | 91 (89) |
| Benin | 0.37 | 39.5  (23.44 to 60.32) | 7.66 | 31.84 | 146 (149) | 0.84  (0.5 to 1.29) | 0.18 | 0.67 | 143 (149) |
| Bermuda | 0.82 | 31.59  (22.72 to 42.95) | 4.42 | 27.17 | 127 (124) | 0.68  (0.49 to 0.92) | 0.09 | 0.59 | 127 (125) |
| Bhutan | 0.47 | 34.56  (21.18 to 54.01) | 5.04 | 29.52 | 137 (138) | 0.73  (0.45 to 1.13) | 0.13 | 0.59 | 129 (138) |
| Bolivia (Plurinational State of) | 0.60 | 13.39  (8.23 to 20.63) | 4.72 | 8.67 | 42 (42) | 0.28  (0.17 to 0.43) | 0.1 | 0.18 | 40 (40) |
| Bosnia and Herzegovina | 0.72 | 17.19  (11.25 to 23.28) | 4.45 | 12.75 | 66 (63) | 0.37  (0.24 to 0.5) | 0.09 | 0.28 | 70 (66) |
| Botswana | 0.64 | 73.25  (44.78 to 110.05) | 4.58 | 68.67 | 175 (174) | 1.57  (0.97 to 2.36) | 0.1 | 1.48 | 176 (174) |
| Brazil | 0.65 | 52.65  (48.69 to 56.69) | 4.57 | 48.08 | 166 (164) | 1.12  (1.04 to 1.21) | 0.1 | 1.02 | 166 (164) |
| Brunei Darussalam | 0.81 | 12.68  (8.97 to 17.79) | 4.44 | 8.24 | 39 (37) | 0.27  (0.19 to 0.38) | 0.09 | 0.18 | 41 (38) |
| Bulgaria | 0.77 | 26.13  (21.19 to 31.56) | 4.46 | 21.67 | 108 (105) | 0.56  (0.45 to 0.68) | 0.09 | 0.47 | 109 (107) |
| Burkina Faso | 0.29 | 42.83  (25.11 to 66.75) | 9.71 | 33.12 | 149 (156) | 0.92  (0.54 to 1.43) | 0.21 | 0.71 | 151 (157) |
| Burundi | 0.29 | 92.12  (58.64 to 140.51) | 9.8 | 82.31 | 182 (184) | 1.95  (1.25 to 2.97) | 0.21 | 1.74 | 182 (184) |
| Cabo Verde | 0.53 | 133  (81.51 to 206.1) | 4.75 | 128.25 | 198 (198) | 2.85  (1.75 to 4.42) | 0.1 | 2.75 | 198 (198) |
| Cambodia | 0.47 | 29.78  (19 to 45.31) | 6.08 | 23.7 | 115 (117) | 0.62  (0.4 to 0.94) | 0.13 | 0.49 | 114 (114) |
| Cameroon | 0.48 | 56.5  (31.61 to 93.38) | 5.47 | 51.02 | 167 (166) | 1.21  (0.68 to 2) | 0.1 | 1.11 | 167 (166) |
| Canada | 0.87 | 24.76  (19.97 to 30.55) | 4.4 | 20.36 | 100 (96) | 0.52  (0.42 to 0.64) | 0.09 | 0.43 | 99 (95) |
| Central African Republic | 0.31 | 106.92  (60.31 to 170.53) | 9.78 | 97.14 | 191 (191) | 2.3  (1.3 to 3.65) | 0.21 | 2.09 | 191 (192) |
| Chad | 0.24 | 41.86  (25.74 to 65.32) | 9.65 | 32.21 | 148 (155) | 0.9  (0.55 to 1.4) | 0.21 | 0.69 | 150 (155) |
| Chile | 0.77 | 15.59  (12.86 to 18.98) | 4.41 | 11.18 | 59 (55) | 0.32  (0.27 to 0.39) | 0.09 | 0.23 | 57 (53) |
| China | 0.72 | 84.81  (65.25 to 108.68) | 4.44 | 80.37 | 181 (180) | 1.79  (1.38 to 2.3) | 0.09 | 1.7 | 181 (180) |
| Colombia | 0.66 | 13.24  (9.98 to 17.21) | 4.55 | 8.69 | 43 (40) | 0.27  (0.2 to 0.35) | 0.1 | 0.17 | 39 (37) |
| Comoros | 0.48 | 107.76  (69.87 to 162.61) | 5.37 | 102.39 | 193 (192) | 2.28  (1.48 to 3.44) | 0.1 | 2.18 | 193 (191) |
| Congo | 0.58 | 94.03  (55.1 to 153.96) | 4.69 | 89.34 | 188 (186) | 2.01  (1.18 to 3.29) | 0.1 | 1.91 | 188 (186) |
| Cook Islands | 0.78 | 21.72  (12.83 to 34.87) | 4.44 | 17.28 | 83 (81) | 0.45  (0.27 to 0.72) | 0.09 | 0.36 | 82 (80) |
| Costa Rica | 0.70 | 12.22  (9.54 to 15.46) | 4.45 | 7.77 | 38 (36) | 0.25  (0.2 to 0.32) | 0.09 | 0.16 | 37 (35) |
| Coted'Ivoire | 0.43 | 10.78  (6.49 to 16.82) | 6.64 | 4.15 | 22 (31) | 0.23  (0.14 to 0.36) | 0.14 | 0.09 | 24 (31) |
| Croatia | 0.80 | 21.92  (16.7 to 28.71) | 4.49 | 17.43 | 85 (83) | 0.47  (0.36 to 0.62) | 0.09 | 0.38 | 88 (86) |
| Cuba | 0.67 | 41.52  (31.27 to 54.71) | 4.5 | 37.03 | 156 (153) | 0.91  (0.68 to 1.2) | 0.1 | 0.81 | 158 (156) |
| Cyprus | 0.84 | 7.49  (4.98 to 11.03) | 4.39 | 3.1 | 17 (14) | 0.16  (0.11 to 0.24) | 0.09 | 0.07 | 17 (14) |
| Czechia | 0.83 | 21.96  (16.83 to 28.01) | 4.43 | 17.53 | 86 (84) | 0.47  (0.36 to 0.6) | 0.09 | 0.38 | 87 (85) |
| Democratic People's Republic of Korea | 0.57 | 93.63  (52.85 to 153.46) | 4.69 | 88.94 | 187 (185) | 1.99  (1.13 to 3.25) | 0.1 | 1.89 | 187 (185) |
| Democratic Republic of the Congo | 0.38 | 64.42  (38.27 to 99.48) | 7.05 | 57.37 | 169 (169) | 1.38  (0.82 to 2.12) | 0.15 | 1.23 | 169 (169) |
| Denmark | 0.90 | 23.89  (19.22 to 29.38) | 4.45 | 19.44 | 94 (92) | 0.51  (0.41 to 0.63) | 0.09 | 0.42 | 96 (92) |
| Djibouti | 0.49 | 98.03  (57.46 to 158.38) | 5.9 | 92.13 | 189 (188) | 2.1  (1.23 to 3.38) | 0.11 | 1.99 | 190 (188) |
| Dominica | 0.75 | 41.15  (27.41 to 61.97) | 4.43 | 36.72 | 155 (152) | 0.89  (0.59 to 1.33) | 0.09 | 0.79 | 155 (152) |
| Dominican Republic | 0.62 | 25.77  (16.88 to 37.47) | 4.72 | 21.05 | 104 (102) | 0.55  (0.36 to 0.8) | 0.1 | 0.45 | 106 (104) |
| Ecuador | 0.66 | 8.97  (6.38 to 11.95) | 4.52 | 4.45 | 24 (22) | 0.18  (0.13 to 0.24) | 0.1 | 0.09 | 23 (21) |
| Egypt | 0.61 | 7.27  (4.91 to 10.36) | 4.73 | 2.55 | 15 (13) | 0.15  (0.1 to 0.22) | 0.1 | 0.05 | 15 (13) |
| El Salvador | 0.56 | 17.88  (13.01 to 23.85) | 4.76 | 13.12 | 71 (67) | 0.37  (0.27 to 0.49) | 0.1 | 0.27 | 67 (65) |
| Equatorial Guinea | 0.66 | 73.89  (39.83 to 124.05) | 4.55 | 69.34 | 176 (175) | 1.57  (0.85 to 2.64) | 0.1 | 1.48 | 177 (175) |
| Eritrea | 0.40 | 139.48  (80.39 to 227.59) | 7.35 | 132.13 | 199 (199) | 2.98  (1.72 to 4.86) | 0.18 | 2.8 | 199 (199) |
| Estonia | 0.84 | 15.12  (11.26 to 19.47) | 4.38 | 10.74 | 57 (52) | 0.33  (0.25 to 0.43) | 0.09 | 0.24 | 59 (55) |
| Eswatini | 0.59 | 208.56  (115.09 to 330.71) | 4.76 | 203.8 | 203 (203) | 4.51  (2.49 to 7.16) | 0.1 | 4.41 | 203 (203) |
| Ethiopia | 0.36 | 38.71  (28.79 to 53.18) | 9.51 | 29.2 | 135 (145) | 0.82  (0.61 to 1.12) | 0.2 | 0.62 | 137 (145) |
| Fiji | 0.68 | 22.57  (13.51 to 35.99) | 4.47 | 18.1 | 88 (86) | 0.47  (0.28 to 0.75) | 0.1 | 0.37 | 84 (82) |
| Finland | 0.86 | 17.96  (15.76 to 20.35) | 4.42 | 13.55 | 72 (68) | 0.38  (0.33 to 0.43) | 0.09 | 0.28 | 73 (69) |
| France | 0.84 | 23.85  (18.88 to 29.93) | 4.43 | 19.42 | 93 (91) | 0.5  (0.4 to 0.63) | 0.09 | 0.41 | 93 (91) |
| Gabon | 0.63 | 87.46  (49.3 to 141.09) | 4.58 | 82.88 | 183 (181) | 1.88  (1.06 to 3.02) | 0.1 | 1.78 | 183 (181) |
| Gambia | 0.41 | 15.79  (9.97 to 23.81) | 7.05 | 8.74 | 44 (56) | 0.34  (0.21 to 0.51) | 0.14 | 0.19 | 46 (56) |
| Georgia | 0.73 | 7.57  (6.51 to 8.79) | 4.44 | 3.13 | 18 (15) | 0.16  (0.14 to 0.19) | 0.09 | 0.07 | 19 (17) |
| Germany | 0.90 | 24.19  (19.53 to 29.98) | 4.44 | 19.75 | 95 (93) | 0.52  (0.42 to 0.64) | 0.09 | 0.42 | 97 (94) |
| Ghana | 0.56 | 28.14  (17.19 to 42.05) | 4.79 | 23.35 | 113 (112) | 0.59  (0.36 to 0.88) | 0.1 | 0.49 | 113 (111) |
| Greece | 0.79 | 14.07  (12.29 to 16.08) | 4.44 | 9.63 | 48 (43) | 0.3  (0.26 to 0.34) | 0.09 | 0.21 | 50 (47) |
| Greenland | 0.83 | 77.39  (49.92 to 112.37) | 4.43 | 72.96 | 179 (177) | 1.67  (1.08 to 2.42) | 0.09 | 1.57 | 180 (178) |
| Grenada | 0.67 | 41.15  (30.34 to 54.65) | 4.52 | 36.63 | 154 (151) | 0.88  (0.65 to 1.17) | 0.1 | 0.79 | 154 (151) |
| Guam | 0.80 | 30  (22.19 to 39.32) | 4.4 | 25.6 | 121 (119) | 0.62  (0.46 to 0.82) | 0.09 | 0.53 | 119 (116) |
| Guatemala | 0.54 | 15.04  (12.28 to 17.99) | 4.68 | 10.36 | 55 (51) | 0.31  (0.25 to 0.37) | 0.1 | 0.21 | 52 (50) |
| Guinea | 0.34 | 10.81  (6.67 to 16.79) | 9.76 | 1.06 | 8 (32) | 0.23  (0.14 to 0.36) | 0.21 | 0.02 | 8 (32) |
| Guinea-Bissau | 0.35 | 69.98  (42.56 to 107.58) | 9.47 | 60.51 | 172 (172) | 1.5  (0.91 to 2.3) | 0.2 | 1.29 | 172 (173) |
| Guyana | 0.65 | 25.63  (17.44 to 36.45) | 4.58 | 21.05 | 105 (101) | 0.55  (0.37 to 0.78) | 0.1 | 0.45 | 104 (102) |
| Haiti | 0.45 | 37.91  (22.85 to 58.33) | 6.65 | 31.26 | 143 (144) | 0.81  (0.49 to 1.24) | 0.14 | 0.67 | 144 (144) |
| Honduras | 0.51 | 7.7  (4.49 to 12.25) | 4.73 | 2.97 | 16 (17) | 0.16  (0.1 to 0.26) | 0.1 | 0.06 | 16 (15) |
| Hungary | 0.79 | 22.97  (18.04 to 28.82) | 4.45 | 18.52 | 91 (88) | 0.49  (0.39 to 0.62) | 0.09 | 0.4 | 92 (90) |
| Iceland | 0.88 | 33.47  (26.32 to 41.93) | 4.38 | 29.08 | 134 (133) | 0.71  (0.56 to 0.89) | 0.09 | 0.61 | 134 (131) |
| India | 0.58 | 36.59  (31.57 to 43.67) | 4.79 | 31.81 | 145 (140) | 0.77  (0.66 to 0.91) | 0.1 | 0.67 | 145 (141) |
| Indonesia | 0.66 | 15.22  (11.44 to 20.17) | 4.55 | 10.67 | 56 (53) | 0.32  (0.24 to 0.42) | 0.1 | 0.22 | 55 (52) |
| Iran (Islamic Republic of) | 0.70 | 26.68  (23.54 to 30.16) | 4.44 | 22.25 | 111 (109) | 0.55  (0.48 to 0.62) | 0.09 | 0.46 | 107 (105) |
| Iraq | 0.66 | 9.26  (6.09 to 14.03) | 4.52 | 4.74 | 28 (24) | 0.2  (0.13 to 0.3) | 0.1 | 0.1 | 28 (25) |
| Ireland | 0.87 | 31.49  (25.11 to 39.25) | 4.43 | 27.07 | 125 (123) | 0.67  (0.53 to 0.83) | 0.09 | 0.57 | 125 (123) |
| Israel | 0.81 | 8.94  (7.13 to 11) | 4.45 | 4.48 | 25 (21) | 0.19  (0.15 to 0.23) | 0.09 | 0.1 | 27 (23) |
| Italy | 0.81 | 11.24  (10.49 to 12.05) | 4.41 | 6.83 | 35 (34) | 0.24  (0.22 to 0.26) | 0.09 | 0.14 | 35 (34) |
| Jamaica | 0.68 | 24.29  (16.09 to 34.54) | 4.45 | 19.84 | 96 (94) | 0.52  (0.34 to 0.74) | 0.09 | 0.42 | 98 (96) |
| Japan | 0.87 | 13.36  (12.89 to 13.86) | 4.45 | 8.9 | 45 (41) | 0.28  (0.27 to 0.29) | 0.09 | 0.19 | 44 (42) |
| Jordan | 0.73 | 6.37  (4.14 to 9.37) | 4.43 | 1.94 | 11 (8) | 0.13  (0.09 to 0.2) | 0.09 | 0.04 | 11 (8) |
| Kazakhstan | 0.73 | 34.33  (29.88 to 38.75) | 4.44 | 29.89 | 141 (136) | 0.72  (0.63 to 0.82) | 0.09 | 0.63 | 140 (137) |
| Kenya | 0.52 | 89.22  (64.47 to 127.18) | 4.79 | 84.43 | 185 (182) | 1.89  (1.37 to 2.68) | 0.1 | 1.79 | 185 (182) |
| Kiribati | 0.53 | 72.67  (41.68 to 117.09) | 4.64 | 68.02 | 174 (173) | 1.49  (0.86 to 2.39) | 0.1 | 1.39 | 174 (172) |
| Kuwait | 0.85 | 5.38  (3.99 to 7.16) | 4.43 | 0.95 | 7 (5) | 0.11  (0.08 to 0.15) | 0.09 | 0.02 | 6 (5) |
| Kyrgyzstan | 0.60 | 20.15  (14.91 to 25.91) | 4.63 | 15.52 | 77 (75) | 0.42  (0.31 to 0.54) | 0.1 | 0.32 | 77 (76) |
| Lao People's Democratic Republic | 0.49 | 26.41  (16.39 to 41.28) | 4.75 | 21.66 | 107 (107) | 0.55  (0.34 to 0.85) | 0.1 | 0.44 | 103 (103) |
| Latvia | 0.83 | 21.58  (16.3 to 27.99) | 4.45 | 17.14 | 82 (80) | 0.47  (0.36 to 0.61) | 0.09 | 0.38 | 86 (84) |
| Lebanon | 0.74 | 5.74  (3.8 to 8.21) | 4.44 | 1.3 | 9 (7) | 0.12  (0.08 to 0.17) | 0.09 | 0.03 | 9 (6) |
| Lesotho | 0.51 | 179.93  (107.94 to 272.11) | 4.78 | 175.15 | 202 (202) | 3.9  (2.34 to 5.88) | 0.1 | 3.8 | 202 (202) |
| Liberia | 0.35 | 48.46  (27.94 to 78.23) | 9.64 | 38.82 | 159 (161) | 1.03  (0.6 to 1.67) | 0.2 | 0.83 | 161 (161) |
| Libya | 0.73 | 14.79  (9.18 to 22.54) | 4.45 | 10.34 | 54 (50) | 0.32  (0.2 to 0.48) | 0.09 | 0.22 | 56 (51) |
| Lithuania | 0.86 | 29.91  (23.12 to 38.9) | 4.39 | 25.52 | 120 (118) | 0.66  (0.51 to 0.86) | 0.09 | 0.57 | 124 (122) |
| Luxembourg | 0.88 | 18.83  (15.84 to 22.4) | 4.43 | 14.4 | 75 (71) | 0.41  (0.34 to 0.49) | 0.09 | 0.31 | 75 (71) |
| Madagascar | 0.40 | 100.37  (60.93 to 156.51) | 7.4 | 92.97 | 190 (189) | 2.12  (1.29 to 3.3) | 0.17 | 1.95 | 189 (189) |
| Malawi | 0.38 | 271.01  (180.94 to 399.36) | 7.5 | 263.51 | 204 (204) | 5.76  (3.86 to 8.45) | 0.17 | 5.59 | 204 (204) |
| Malaysia | 0.74 | 20.45  (14.23 to 28.82) | 4.44 | 16.01 | 79 (77) | 0.44  (0.31 to 0.62) | 0.09 | 0.34 | 79 (77) |
| Maldives | 0.65 | 9.87  (6.36 to 14.53) | 4.57 | 5.31 | 30 (28) | 0.21  (0.13 to 0.3) | 0.1 | 0.11 | 30 (28) |
| Mali | 0.27 | 19.27  (12.26 to 29) | 9.7 | 9.57 | 46 (72) | 0.41  (0.26 to 0.62) | 0.21 | 0.2 | 47 (73) |
| Malta | 0.80 | 18.38  (14.33 to 23.2) | 4.42 | 13.96 | 74 (70) | 0.4  (0.31 to 0.5) | 0.09 | 0.3 | 74 (70) |
| Marshall Islands | 0.57 | 30.43  (18.02 to 48.22) | 4.78 | 25.64 | 122 (120) | 0.62  (0.37 to 0.99) | 0.1 | 0.52 | 117 (117) |
| Mauritania | 0.50 | 34.53  (20.36 to 55.51) | 4.79 | 29.74 | 139 (137) | 0.74  (0.44 to 1.19) | 0.1 | 0.64 | 141 (139) |
| Mauritius | 0.72 | 43.91  (37.51 to 50.21) | 4.44 | 39.47 | 160 (158) | 0.93  (0.79 to 1.06) | 0.09 | 0.83 | 160 (158) |
| Mexico | 0.66 | 14.75  (12.93 to 16.77) | 4.53 | 10.22 | 53 (49) | 0.3  (0.26 to 0.35) | 0.1 | 0.21 | 51 (48) |
| Micronesia (Federated States of) | 0.59 | 33.22  (19.69 to 53.63) | 4.73 | 28.49 | 130 (130) | 0.68  (0.41 to 1.1) | 0.1 | 0.59 | 128 (126) |
| Monaco | 0.91 | 49.11  (29.43 to 73.71) | 4.44 | 44.67 | 164 (162) | 1.03  (0.62 to 1.55) | 0.1 | 0.94 | 164 (162) |
| Mongolia | 0.62 | 110.27  (78.13 to 148.36) | 4.62 | 105.66 | 194 (193) | 2.33  (1.65 to 3.12) | 0.1 | 2.23 | 194 (193) |
| Montenegro | 0.80 | 20.56  (15.44 to 26.7) | 4.41 | 16.16 | 80 (78) | 0.44  (0.33 to 0.58) | 0.09 | 0.35 | 80 (78) |
| Morocco | 0.56 | 5.7  (3.58 to 9.1) | 4.79 | 0.92 | 6 (6) | 0.12  (0.08 to 0.19) | 0.1 | 0.02 | 7 (7) |
| Mozambique | 0.33 | 55  (33.25 to 86.61) | 9.67 | 45.34 | 165 (165) | 1.19  (0.72 to 1.87) | 0.21 | 0.98 | 165 (165) |
| Myanmar | 0.53 | 23.55  (14.85 to 36.19) | 4.65 | 18.9 | 92 (90) | 0.49  (0.31 to 0.75) | 0.1 | 0.38 | 89 (87) |
| Namibia | 0.62 | 25.04  (14.77 to 41.13) | 4.69 | 20.36 | 99 (97) | 0.54  (0.32 to 0.88) | 0.1 | 0.44 | 102 (100) |
| Nauru | 0.63 | 43.25  (25.31 to 69.28) | 4.7 | 38.55 | 158 (157) | 0.89  (0.52 to 1.43) | 0.1 | 0.8 | 156 (154) |
| Nepal | 0.43 | 40.47  (25.27 to 63.39) | 6.64 | 33.83 | 152 (150) | 0.85  (0.53 to 1.33) | 0.14 | 0.71 | 152 (150) |
| Netherlands | 0.89 | 25.37  (20.69 to 31.03) | 4.43 | 20.94 | 103 (100) | 0.54  (0.44 to 0.66) | 0.09 | 0.45 | 105 (101) |
| New Zealand | 0.85 | 15.45  (12.94 to 18.34) | 4.39 | 11.06 | 58 (54) | 0.33  (0.28 to 0.39) | 0.09 | 0.23 | 58 (54) |
| Nicaragua | 0.52 | 6.59  (4.54 to 9.41) | 4.64 | 1.95 | 12 (11) | 0.14  (0.09 to 0.2) | 0.1 | 0.03 | 10 (10) |
| Niger | 0.17 | 28.28  (17.17 to 45.22) | 18.25 | 10.04 | 52 (113) | 0.6  (0.37 to 0.96) | 0.39 | 0.21 | 54 (112) |
| Nigeria | 0.50 | 31.85  (19.69 to 45.82) | 4.69 | 27.16 | 126 (125) | 0.68  (0.42 to 0.98) | 0.1 | 0.58 | 126 (124) |
| Niue | 0.73 | 21.77  (12.9 to 35.51) | 4.45 | 17.32 | 84 (82) | 0.45  (0.26 to 0.73) | 0.09 | 0.35 | 81 (79) |
| North Macedonia | 0.75 | 10.41  (7.4 to 14.28) | 4.43 | 5.98 | 33 (30) | 0.23  (0.16 to 0.31) | 0.09 | 0.13 | 33 (30) |
| Northern Mariana Islands | 0.77 | 20.26  (13.25 to 30.49) | 4.44 | 15.81 | 78 (76) | 0.42  (0.27 to 0.63) | 0.09 | 0.32 | 78 (74) |
| Norway | 0.92 | 12.87  (11.79 to 14) | 4.45 | 8.42 | 40 (38) | 0.28  (0.25 to 0.3) | 0.09 | 0.18 | 42 (39) |
| Oman | 0.77 | 7.67  (4.96 to 11.52) | 4.42 | 3.24 | 19 (16) | 0.16  (0.11 to 0.25) | 0.09 | 0.07 | 18 (16) |
| Pakistan | 0.50 | 64.28  (47.11 to 88.91) | 4.79 | 59.49 | 170 (168) | 1.35  (0.99 to 1.86) | 0.1 | 1.25 | 170 (168) |
| Palau | 0.75 | 35.58  (21.98 to 54.57) | 4.43 | 31.15 | 142 (139) | 0.72  (0.45 to 1.11) | 0.09 | 0.63 | 139 (136) |
| Palestine | 0.63 | 5.19  (3.65 to 7.21) | 4.61 | 0.58 | 4 (4) | 0.11  (0.08 to 0.15) | 0.1 | 0.01 | 4 (3) |
| Panama | 0.71 | 9.02  (6.79 to 11.43) | 4.43 | 4.6 | 26 (23) | 0.19  (0.14 to 0.24) | 0.09 | 0.09 | 25 (22) |
| Papua New Guinea | 0.42 | 14.63  (8.91 to 23.42) | 7.78 | 6.85 | 36 (48) | 0.3  (0.18 to 0.48) | 0.14 | 0.15 | 36 (46) |
| Paraguay | 0.64 | 33  (20.47 to 49.73) | 4.59 | 28.42 | 129 (129) | 0.71  (0.44 to 1.07) | 0.1 | 0.61 | 133 (132) |
| Peru | 0.66 | 8.66  (5.56 to 12.8) | 4.51 | 4.15 | 23 (20) | 0.18  (0.11 to 0.26) | 0.1 | 0.08 | 21 (19) |
| Philippines | 0.65 | 17  (13.96 to 20.47) | 4.58 | 12.42 | 64 (62) | 0.35  (0.29 to 0.42) | 0.1 | 0.25 | 63 (59) |
| Poland | 0.81 | 24.6  (22.05 to 26.99) | 4.45 | 20.15 | 98 (95) | 0.53  (0.48 to 0.58) | 0.09 | 0.44 | 101 (98) |
| Portugal | 0.74 | 28.9  (22.84 to 36.07) | 4.45 | 24.46 | 116 (114) | 0.62  (0.49 to 0.78) | 0.09 | 0.53 | 118 (115) |
| Puerto Rico | 0.83 | 26.38  (19.33 to 35.46) | 4.39 | 21.99 | 109 (106) | 0.56  (0.41 to 0.75) | 0.09 | 0.47 | 108 (106) |
| Qatar | 0.85 | 10.22  (6.3 to 15.66) | 4.4 | 5.82 | 32 (29) | 0.21  (0.13 to 0.33) | 0.09 | 0.12 | 32 (29) |
| Republic of Korea | 0.89 | 8.4  (6.04 to 11.58) | 4.44 | 3.96 | 20 (18) | 0.18  (0.13 to 0.25) | 0.09 | 0.08 | 22 (20) |
| Republic of Moldova | 0.73 | 12.17  (10.29 to 14.2) | 4.43 | 7.74 | 37 (35) | 0.27  (0.22 to 0.31) | 0.09 | 0.17 | 38 (36) |
| Romania | 0.77 | 26.52  (20.28 to 34.32) | 4.44 | 22.08 | 110 (108) | 0.58  (0.44 to 0.75) | 0.09 | 0.48 | 112 (110) |
| Russian Federation | 0.81 | 32.44  (29.34 to 35.33) | 4.48 | 27.96 | 128 (127) | 0.7  (0.63 to 0.76) | 0.09 | 0.6 | 132 (129) |
| Rwanda | 0.44 | 91.04  (56.09 to 138.28) | 6.64 | 84.4 | 184 (183) | 1.93  (1.19 to 2.92) | 0.14 | 1.79 | 184 (183) |
| Saint Kitts and Nevis | 0.75 | 26.05  (19.09 to 34.77) | 4.41 | 21.64 | 106 (104) | 0.57  (0.42 to 0.75) | 0.09 | 0.47 | 110 (108) |
| Saint Lucia | 0.67 | 45.83  (35.66 to 57.21) | 4.52 | 41.31 | 162 (160) | 0.98  (0.76 to 1.22) | 0.1 | 0.88 | 162 (160) |
| Saint Vincent and the Grenadines | 0.64 | 30.46  (24.98 to 36.85) | 4.59 | 25.87 | 124 (121) | 0.66  (0.54 to 0.8) | 0.1 | 0.56 | 123 (121) |
| Samoa | 0.59 | 9.31  (5.8 to 14.18) | 4.71 | 4.6 | 27 (25) | 0.2  (0.12 to 0.3) | 0.1 | 0.1 | 26 (24) |
| San Marino | 0.89 | 6.39  (3.1 to 10.94) | 4.42 | 1.97 | 13 (9) | 0.14  (0.07 to 0.23) | 0.09 | 0.04 | 13 (9) |
| Sao Tome and Principe | 0.51 | 33.41  (20 to 54.73) | 4.69 | 28.72 | 133 (132) | 0.72  (0.43 to 1.17) | 0.1 | 0.62 | 135 (135) |
| Saudi Arabia | 0.82 | 14.22  (8.98 to 21.87) | 4.44 | 9.78 | 49 (44) | 0.3  (0.19 to 0.46) | 0.09 | 0.2 | 49 (45) |
| Senegal | 0.41 | 38.94  (24.22 to 59.81) | 7.39 | 31.55 | 144 (146) | 0.83  (0.52 to 1.28) | 0.14 | 0.69 | 149 (147) |
| Serbia | 0.79 | 16.32  (10.98 to 23.79) | 4.43 | 11.89 | 61 (58) | 0.36  (0.24 to 0.52) | 0.09 | 0.26 | 65 (62) |
| Seychelles | 0.73 | 57.03  (38.31 to 82.8) | 4.44 | 52.59 | 168 (167) | 1.21  (0.81 to 1.75) | 0.09 | 1.12 | 168 (167) |
| Sierra Leone | 0.36 | 39.17  (23.55 to 62.52) | 9.4 | 29.76 | 140 (147) | 0.84  (0.5 to 1.33) | 0.19 | 0.64 | 142 (148) |
| Singapore | 0.86 | 6.64  (5.36 to 8.14) | 4.44 | 2.2 | 14 (12) | 0.14  (0.11 to 0.17) | 0.09 | 0.05 | 14 (12) |
| Slovakia | 0.81 | 29.5  (19.06 to 44.83) | 4.41 | 25.09 | 119 (116) | 0.64  (0.41 to 0.97) | 0.09 | 0.55 | 122 (119) |
| Slovenia | 0.84 | 17.34  (12.97 to 22.61) | 4.41 | 12.93 | 68 (65) | 0.37  (0.28 to 0.49) | 0.09 | 0.28 | 72 (67) |
| Solomon Islands | 0.43 | 31.22  (18.37 to 49.75) | 6.67 | 24.55 | 117 (122) | 0.64  (0.38 to 1.03) | 0.14 | 0.5 | 115 (120) |
| Somalia | 0.08 | 132.71  (79.47 to 212.92) | 132.71 | 0 | 1 (197) | 2.83  (1.69 to 4.53) | 2.83 | 0 | 1 (197) |
| South Africa | 0.68 | 104.7  (88.86 to 123.98) | 4.49 | 100.21 | 192 (190) | 2.23  (1.9 to 2.64) | 0.1 | 2.14 | 192 (190) |
| South Sudan | 0.28 | 130.53  (79.47 to 196.88) | 9.81 | 120.72 | 195 (195) | 2.78  (1.69 to 4.18) | 0.21 | 2.57 | 195 (195) |
| Spain | 0.77 | 17.28  (13.73 to 21.32) | 4.43 | 12.85 | 67 (64) | 0.36  (0.29 to 0.45) | 0.09 | 0.27 | 68 (63) |
| Sri Lanka | 0.70 | 36.63  (20.78 to 55.87) | 4.49 | 32.14 | 147 (141) | 0.77  (0.44 to 1.17) | 0.09 | 0.68 | 146 (140) |
| Sudan | 0.54 | 37.88  (18.43 to 62.69) | 4.73 | 33.15 | 150 (143) | 0.79  (0.39 to 1.3) | 0.1 | 0.69 | 148 (143) |
| Suriname | 0.63 | 17.52  (11.37 to 26.33) | 4.58 | 12.94 | 69 (66) | 0.37  (0.24 to 0.56) | 0.1 | 0.28 | 71 (68) |
| Sweden | 0.89 | 10.97  (8.91 to 13.5) | 4.41 | 6.56 | 34 (33) | 0.23  (0.19 to 0.29) | 0.09 | 0.14 | 34 (33) |
| Switzerland | 0.93 | 16.64  (12.99 to 20.67) | 4.45 | 12.19 | 62 (59) | 0.35  (0.28 to 0.44) | 0.1 | 0.26 | 64 (61) |
| Syrian Arab Republic | 0.62 | 6.58  (4.57 to 9.35) | 4.69 | 1.89 | 10 (10) | 0.14  (0.1 to 0.2) | 0.1 | 0.04 | 12 (11) |
| Taiwan (Province of China) | 0.87 | 126.58  (109.12 to 145.5) | 4.44 | 122.15 | 196 (194) | 2.74  (2.37 to 3.15) | 0.09 | 2.65 | 196 (194) |
| Tajikistan | 0.54 | 44.97  (30.9 to 64.97) | 4.71 | 40.26 | 161 (159) | 0.93  (0.64 to 1.34) | 0.1 | 0.83 | 159 (159) |
| Thailand | 0.68 | 75.65  (48.61 to 111.99) | 4.45 | 71.2 | 178 (176) | 1.59  (1.02 to 2.35) | 0.1 | 1.49 | 178 (176) |
| Timor-Leste | 0.44 | 19.67  (12.34 to 29.44) | 6.65 | 13.02 | 70 (74) | 0.41  (0.26 to 0.61) | 0.14 | 0.27 | 66 (72) |
| Togo | 0.41 | 49.64  (29.06 to 78.11) | 7.26 | 42.38 | 163 (163) | 1.07  (0.63 to 1.68) | 0.14 | 0.92 | 163 (163) |
| Tokelau | 0.69 | 18.04  (10.77 to 28.41) | 4.43 | 13.61 | 73 (69) | 0.37  (0.22 to 0.58) | 0.09 | 0.27 | 69 (64) |
| Tonga | 0.63 | 16.92  (10.14 to 26.64) | 4.68 | 12.24 | 63 (61) | 0.35  (0.21 to 0.55) | 0.1 | 0.25 | 62 (60) |
| Trinidad and Tobago | 0.77 | 21.57  (15.36 to 29.36) | 4.44 | 17.13 | 81 (79) | 0.46  (0.33 to 0.63) | 0.09 | 0.36 | 83 (81) |
| Tunisia | 0.68 | 4.42  (2.71 to 6.82) | 4.42 | 0 | 2 (1) | 0.09  (0.06 to 0.14) | 0.09 | 0 | 2 (1) |
| Turkey | 0.71 | 14.26  (9.42 to 20.17) | 4.42 | 9.84 | 50 (45) | 0.3  (0.2 to 0.42) | 0.09 | 0.2 | 48 (44) |
| Turkmenistan | 0.68 | 77.87  (59.02 to 102.67) | 4.46 | 73.41 | 180 (178) | 1.62  (1.23 to 2.14) | 0.1 | 1.53 | 179 (177) |
| Tuvalu | 0.58 | 25.08  (15.05 to 39.48) | 4.68 | 20.4 | 101 (98) | 0.52  (0.31 to 0.81) | 0.1 | 0.42 | 95 (93) |
| Uganda | 0.42 | 132.15  (81.42 to 200.76) | 7.24 | 124.91 | 197 (196) | 2.81  (1.73 to 4.26) | 0.14 | 2.66 | 197 (196) |
| Ukraine | 0.76 | 29.27  (18.16 to 43.53) | 4.47 | 24.81 | 118 (115) | 0.63  (0.39 to 0.94) | 0.09 | 0.53 | 120 (118) |
| United Arab Emirates | 0.85 | 8.42  (5.34 to 12.5) | 4.44 | 3.97 | 21 (19) | 0.17  (0.11 to 0.25) | 0.09 | 0.07 | 20 (18) |
| United Kingdom | 0.86 | 39.31  (37.93 to 40.84) | 4.44 | 34.87 | 153 (148) | 0.83  (0.8 to 0.86) | 0.09 | 0.73 | 153 (146) |
| United Republic of Tanzania | 0.45 | 95.31  (60.57 to 143.5) | 6.63 | 88.68 | 186 (187) | 2.02  (1.28 to 3.03) | 0.14 | 1.87 | 186 (187) |
| United States Virgin Islands | 0.82 | 33.73  (20.94 to 51.57) | 4.43 | 29.31 | 136 (134) | 0.71  (0.44 to 1.09) | 0.09 | 0.62 | 136 (133) |
| United States of America | 0.86 | 25.1  (24.09 to 26.17) | 4.39 | 20.71 | 102 (99) | 0.53  (0.5 to 0.55) | 0.09 | 0.43 | 100 (97) |
| Uruguay | 0.72 | 32.98  (26.65 to 40.72) | 4.43 | 28.55 | 132 (128) | 0.69  (0.56 to 0.85) | 0.09 | 0.6 | 131 (128) |
| Uzbekistan | 0.66 | 37.75  (28.64 to 49.14) | 4.53 | 33.22 | 151 (142) | 0.77  (0.59 to 1.01) | 0.1 | 0.68 | 147 (142) |
| Vanuatu | 0.47 | 25.85  (15.4 to 40.6) | 5.98 | 19.87 | 97 (103) | 0.53  (0.32 to 0.83) | 0.12 | 0.41 | 94 (99) |
| Venezuela (Bolivarian Republic of) | 0.60 | 14.28  (9.94 to 19.68) | 4.7 | 9.59 | 47 (47) | 0.29  (0.2 to 0.4) | 0.1 | 0.19 | 45 (43) |
| Viet Nam | 0.63 | 19.33  (11.71 to 30.84) | 4.62 | 14.71 | 76 (73) | 0.42  (0.25 to 0.67) | 0.1 | 0.32 | 76 (75) |
| Yemen | 0.45 | 32.38  (14.67 to 53.34) | 6.67 | 25.72 | 123 (126) | 0.69  (0.31 to 1.13) | 0.14 | 0.55 | 121 (127) |
| Zambia | 0.51 | 157.69  (87.27 to 285.22) | 4.66 | 153.03 | 200 (200) | 3.35  (1.86 to 6) | 0.1 | 3.25 | 200 (200) |
| Zimbabwe | 0.47 | 178.33  (108.89 to 266.69) | 6.08 | 172.25 | 201 (201) | 3.79  (2.31 to 5.67) | 0.13 | 3.67 | 201 (201) |

UI=uncertainty interval. DALY=disability-adjusted life year. ASR=age-standardized rate.

**Supplementary Table 7. Global DALYs attributable to Level 2 risk factors and AAPC in 1990 and 2021, for both sexes**

| **Categories** | **DALYs** | | | | |
| --- | --- | --- | --- | --- | --- |
|  | **1990** | | **2021** | |  |
|  | **Counts**  **(95%UI)** | **ASDR**  **(95%UI)** | **Counts**  **(95%UI)** | **ASDR**  **(95%UI)** | **AAPC**  **(95%CI)** |
| Tobacco | 541172.39  (437725.03-663067.39) | 29.20  (23.63-35.78) | 398929.79  (315939.67-496386.28) | 11.51  (9.12-14.31) | -3.00  (-3.21--2.80) |
| Dietary risks | 351310.37  (77856.22-694170.68) | 18.73  (4.15-36.98) | 212069.65  (431081.44-45102.68) | 6.16  (1.31-12.52) | -3.56  (-3.74--3.39) |
| Alcohol use | 333199.51  (240195.64-441218.90) | 17.69  (12.77-23.40) | 285968.11  (208690.39-376596.53) | 8.29  (6.05-10.91) | -2.44  (-2.72--2.16) |

UI=uncertainty interval. CI=confidence interval. DALY=disability-adjusted life year. ASDR=age-standardized DALYs rate. AAPC=average annual percent change.

**Supplementary Table 8. Proportion of Attributable Risk Factors for Early-Onset Esophageal Cancer DALYs in 1990 and 2021, by Sex**

| **Categories** | **Smoking** | | | | **Alcohol use** | | | | **Diet low in vegetables** | | | | **Chewing tobacco** | | | |
| --- | --- | --- | --- | --- | --- | --- | --- | --- | --- | --- | --- | --- | --- | --- | --- | --- |
|  | **1990** | | **2021** | | **1990** | | **2021** | | **1990** | | **2021** | | **1990** | | **2021** | |
|  | **ASDR**  **(95%UI)** | **Proportion**  **%** | **ASDR**  **(95%UI)** | **Proportion**  **%** | **ASDR**  **(95%UI)** | **Proportion**  **%** | **ASDR**  **(95%UI)** | **Proportion**  **%** | **ASDR**  **(95%UI)** | **Proportion**  **%** | **ASDR**  **(95%UI)** | **Proportion**  **%** | **ASDR**  **(95%UI)** | **Proportion**  **%** | **ASDR**  **(95%UI)** | **Proportion**  **%** |
| Global | 26.56  (21.11-32.80) | 28.2 | 9.44  (7.18-12.02) | 21.1 | 17.69  (12.77-23.40) | 19.1 | 8.29  (6.05-10.91) | 18.4 | 18.73  (-4.15-36.98) | 20.1 | 6.16  (-1.31-12.52) | 13.7 | 3.42  (2.25-5.05) | 3.7 | 2.42  (1.58-3.37) | 5.4 |
| Central Asia | 23.66  (19.10-28.42) | 18.4 | 6.24  (4.79-7.77) | 15.9 | 18.66  (12.84-24.75) | 15.7 | 5.80  (3.96-7.74) | 14.9 | 18.75  (-4.19-39.68) | 15.8 | 2.01  (-0.38-4.89) | 5.2 | 2.27  (1.18-3.88) | 1.8 | 0.82  (0.43-1.43) | 2.1 |
| Central Europe | 14.93  (12.22-17.56) | 38.1 | 6.96  (5.46-8.53) | 30.7 | 11.58  (8.69-14.29) | 29.6 | 7.25  (5.43-9.12) | 31.2 | 6.76  (-1.48-13.93) | 17.3 | 2.37  (-0.51-5.07) | 10.2 | 0.19  (0.10-0.31) | 0.5 | 0.17  (0.09-0.27) | 0.7 |
| Eastern Europe | 17.84  (14.86-20.95) | 37.7 | 10.70  (8.44-12.99) | 35.0 | 13.97  (10.25-17.56) | 29.9 | 9.05  (6.59-11.64) | 29.2 | 8.25  (-1.87-16.76) | 17.7 | 5.35  (-1.11-11.03) | 17.3 | 0.20  (0.10-0.37) | 0.4 | 0.13  (0.06-0.24) | 0.4 |
| Australasia | 5.76  (4.40-7.32) | 26.6 | 4.13  (2.92-5.64) | 19.3 | 6.95  (5.00-9.00) | 32.1 | 6.89  (4.91-9.23) | 31.7 | 4.55  (-1.08-9.09) | 21.1 | 4.41  (-1.04-9.38) | 20.4 | 0.16  (0.07-0.32) | 0.8 | 0.17  (0.07-0.36) | 0.8 |
| High-income Asia Pacific | 11.19  (9.05-13.66) | 38.8 | 2.78  (2.15-3.47) | 24.8 | 8.26  (6.02-10.51) | 28.4 | 2.95  (2.09-3.82) | 25.3 | 3.82  (-0.80-8.37) | 13.2 | 1.44  (-0.27-3.35) | 12.4 | 0.29  (0.12-0.55) | 1.0 | 0.13  (0.06-0.23) | 1.1 |
| High-income North America | 11.75  (9.37-14.01) | 39.2 | 5.69  (4.35-7.11) | 23.1 | 7.67  (5.41-9.89) | 25.9 | 6.63  (4.74-8.44) | 26.3 | 5.35  (-1.15-10.71) | 18.1 | 4.39  (-0.97-8.80) | 17.6 | 1.32  (0.64-2.23) | 4.4 | 1.19  (0.54-2.26) | 4.8 |
| Southern Latin America | 14.11  (10.72-17.66) | 28.4 | 4.51  (3.34-5.82) | 21.9 | 14.39  (10.63-18.41) | 29.2 | 5.15  (3.59-6.89) | 24.7 | 10.70  (-2.44-21.81) | 21.6 | 4.20  (-0.99-8.52) | 20.2 | 0.13  (0.07-0.22) | 0.3 | 0.05  (0.03-0.08) | 0.2 |
| Western Europe | 16.01  (12.75-19.00) | 34.6 | 5.23  (4.11-6.32) | 24.1 | 15.64  (11.90-19.19) | 33.7 | 6.83  (5.10-8.61) | 30.4 | 8.44  (-1.90-17.15) | 18.2 | 4.20  (-0.93-8.53) | 18.8 | 0.12  (0.07-0.19) | 0.3 | 0.07  (0.04-0.11) | 0.3 |
| Andean Latin America | 1.25  (0.90-1.70) | 7.2 | 0.68  (0.45-1.01) | 7.0 | 2.83  (1.61-4.07) | 17.5 | 2.02  (1.29-3.02) | 21.2 | 3.80  (-0.86-7.62) | 22.9 | 2.11  (-0.48-4.31) | 22.0 | 0.11  (0.06-0.19) | 0.7 | 0.06  (0.03-0.10) | 0.6 |
| Caribbean | 7.00  (5.35-8.83) | 19.2 | 5.27  (3.83-6.80) | 15.7 | 6.57  (4.58-8.65) | 18.6 | 6.92  (4.81-9.49) | 20.5 | 8.32  (-1.95-16.35) | 23.4 | 7.39  (-1.66-15.29) | 21.8 | 0.24  (0.14-0.39) | 0.7 | 0.28  (0.15-0.47) | 0.8 |
| Central Latin America | 3.10  (2.43-3.81) | 13.2 | 1.07  (0.80-1.36) | 7.8 | 4.29  (2.99-5.58) | 19.9 | 2.84  (1.93-3.73) | 20.4 | 4.86  (-1.13-9.52) | 21.9 | 3.00  (-0.72-6.17) | 21.6 | 0.15  (0.08-0.24) | 0.7 | 0.10  (0.06-0.17) | 0.7 |
| Tropical Latin America | 23.07  (18.39-28.30) | 29.7 | 6.36  (4.68-8.24) | 12.3 | 15.30  (10.87-20.14) | 20.3 | 11.54  (8.14-15.09) | 22.1 | 17.89  (-4.15-34.46) | 23.6 | 11.57  (-2.59-22.73) | 22.2 | 0.37  (0.16-0.74) | 0.5 | 0.28  (0.12-0.53) | 0.5 |
| North Africa and Middle East | 4.77  (3.52-6.27) | 17.1 | 2.81  (2.10-3.62) | 16.7 | 0.63  (0.37-0.95) | 2.4 | 0.35  (0.21-0.51) | 2.1 | 3.96  (-0.84-8.49) | 14.6 | 2.30  (-0.48-4.89) | 13.6 | 0.66  (0.38-1.07) | 2.4 | 0.51  (0.29-0.82) | 3.0 |
| South Asia | 6.29  (4.82-7.91) | 12.0 | 3.37  (2.51-4.34) | 8.4 | 2.49  (0.90-3.86) | 4.9 | 3.17  (1.87-4.53) | 8.0 | 11.65  (-2.84-23.05) | 23.0 | 8.80  (-1.91-18.14) | 22.3 | 11.07  (7.46-15.42) | 21.5 | 7.74  (4.95-10.85) | 19.5 |
| East Asia | 78.28  (59.17-100.87) | 32.2 | 27.38  (19.13-36.90) | 32.9 | 49.47  (33.38-68.46) | 20.8 | 20.38  (13.84-29.22) | 23.9 | 47.23  (-10.30-95.64) | 19.6 | 2.56  (-0.41-8.16) | 3.0 | 3.70  (1.42-7.82) | 1.5 | 1.58  (0.59-3.38) | 1.9 |
| Oceania | 3.69  (2.26-5.64) | 16.6 | 2.92  (1.87-4.41) | 16.4 | 1.50  (0.65-2.66) | 7.2 | 1.18  (0.55-1.98) | 6.8 | 4.99  (-0.96-10.64) | 23.5 | 4.11  (-0.83-8.82) | 23.5 | 1.58  (0.79-2.91) | 7.4 | 1.42  (0.76-2.58) | 8.1 |
| Southeast Asia | 5.79  (4.20-7.89) | 17.6 | 4.52  (3.32-5.93) | 18.5 | 2.52  (1.49-3.78) | 8.0 | 3.85  (2.55-5.48) | 15.7 | 7.35  (-1.69-14.60) | 23.5 | 5.40  (-1.19-10.93) | 22.0 | 1.30  (0.79-1.98) | 4.0 | 0.79  (0.50-1.18) | 3.2 |
| Central Sub-Saharan Africa | 8.48  (5.33-12.52) | 8.1 | 5.43  (3.43-8.22) | 7.6 | 11.09  (3.08-18.69) | 11.1 | 8.99  (3.82-14.49) | 13 | 25.90  (-5.74-53.78) | 25.9 | 17.71  (-3.70-36.30) | 25.5 | 1.82  (0.80-3.60) | 1.7 | 1.14  (0.54-2.19) | 1.6 |
| Eastern Sub-Saharan Africa | 9.33  (6.64-12.30) | 7.0 | 6.27  (4.44-8.43) | 6.3 | 12.26  (4.44-18.36) | 9.6 | 11.52  (6.10-16.91) | 12 | 32.19  (-7.14-61.87) | 25.1 | 23.52  (-4.71-45.63) | 24.5 | 3.95  (2.45-5.97) | 3.0 | 2.82  (1.75-4.28) | 2.9 |
| Southern Sub-Saharan Africa | 40.29  (30.48-50.87) | 21.0 | 16.43  (12.16-21.20) | 13.9 | 38.72  (22.04-51.74) | 20.9 | 22.13  (12.11-29.99) | 19.2 | 43.44  (-9.62-83.99) | 23.5 | 27.68  (-6.27-53.42) | 24.0 | 1.94  (0.98-3.40) | 1.0 | 1.09  (0.58-1.87) | 0.9 |
| Western Sub-Saharan Africa | 1.38  (0.99-1.90) | 6.1 | 1.96  (1.37-2.63) | 5.7 | 2.52(1.24-3.85) | 11.6 | 5.22(3.09-7.69) | 15.8 | 5.02  (-1.06-10.04) | 23.0 | 7.30  (-1.67-14.87) | 21.8 | 0.30  (0.18-0.46) | 1.3 | 0.43  (0.24-0.68) | 1.3 |

UI=uncertainty interval. DALY=disability-adjusted life year. ASDR=age-standardized DALYs rate.

**Supplementary Table 9. Proportion of Attributable Risk Factors for Early-Onset Esophageal Cancer DALYs in 1990 and 2021, by SDI Category and Sex**

| **Categories** | **Smoking** | | | | **Alcohol use** | | | | **Diet low in vegetables** | | | | **Chewing tobacco** | | | |
| --- | --- | --- | --- | --- | --- | --- | --- | --- | --- | --- | --- | --- | --- | --- | --- | --- |
|  | **1990** | | **2021** | | **1990** | | **2021** | | **1990** | | **2021** | | **1990** | | **2021** | |
|  | **ASDR**  **(95%UI)** | **Proportion**  **%** | **ASDR**  **(95%UI)** | **Proportion**  **%** | **ASDR**  **(95%UI)** | **Proportion**  **%** | **ASDR**  **(95%UI)** | **Proportion**  **%** | **ASDR**  **(95%UI)** | **Proportion**  **%** | **ASDR**  **(95%UI)** | **Proportion**  **%** | **ASDR**  **(95%UI)** | **Proportion**  **%** | **ASDR**  **(95%UI)** | **Proportion**  **%** |
| **Both sexes** | | | | | | | | | | | | | | | | |
| Global | 26.56  (21.11-32.80) | 28.16 | 9.44  (7.18-12.02) | 21.12 | 17.69  (12.77-23.40) | 19.08 | 8.29  (6.05-10.91) | 18.43 | 18.73  (-4.15-36.98) | 20.12 | 6.16  (-1.31-12.52) | 13.67 | 3.42  (2.25-5.05) | 3.65 | 2.42  (1.58-3.37) | 5.38 |
| High SDI | 14.50  (11.80-17.08) | 35.85 | 6.34  (5.06-7.66) | 25.6 | 11.83  (9.09-14.61) | 29.34 | 6.73  (5.08-8.44) | 26.51 | 7.35  (-1.62-14.83) | 18.25 | 3.90  (-0.84-7.99) | 15.42 | 0.62  (0.33-0.98) | 1.55 | 0.52  (0.28-0.89) | 2.09 |
| High-middle SDI | 43.50  (33.74-55.03) | 32.47 | 17.80  (12.86-24.03) | 32.48 | 30.43  (21.49-41.05) | 23.1 | 13.86  (9.64-19.50) | 24.78 | 24.37  (-5.21-49.45) | 18.33 | 2.37  (-0.50-5.83) | 4.23 | 1.87  (0.77-3.98) | 1.42 | 1.01  (0.41-2.06) | 1.81 |
| Middle SDI | 40.04  (30.44-52.43) | 29.35 | 10.97  (8.05-14.50) | 23.19 | 24.55  (16.73-34.34) | 18.43 | 9.43  (6.68-12.84) | 19.7 | 27.73  (-6.18-55.67) | 20.64 | 5.45  (-1.15-11.61) | 11.37 | 3.48  (2.00-5.69) | 2.57 | 2.04  (1.22-2.98) | 4.28 |
| Low-middle SDI | 7.00  (5.40-8.64) | 13.66 | 4.30  (3.25-5.37) | 10.46 | 3.31  (1.90-4.68) | 6.7 | 4.18  (2.76-5.75) | 10.37 | 11.13  (-2.69-22.05) | 22.49 | 8.89  (-1.90-17.97) | 22.01 | 7.69  (5.21-10.62) | 15.23 | 5.19  (3.35-7.26) | 12.74 |
| Low SDI | 5.56  (4.17-7.17) | 7.87 | 3.69  (2.71-4.74) | 6.61 | 5.12  (1.97-7.47) | 7.51 | 5.14  (2.86-7.24) | 9.56 | 16.94  (-3.89-33.33) | 24.8 | 13.17  (-2.74-25.69) | 24.4 | 4.89  (3.32-6.95) | 7.04 | 3.23  (2.10-4.52) | 5.89 |
| **Men** | | | | | | | | | | | | | | | | |
| Global | 50.46  (39.99-62.60) | 34.89 | 18.18  (13.82-23.24) | 26.8 | 33.27  (23.93-44.06) | 23.39 | 15.67  (11.48-20.68) | 22.97 | 28.62  (-6.27-57.28) | 19.98 | 8.34  (-1.79-17.32) | 12.21 | 4.92  (2.79-7.97) | 3.43 | 3.56  (2.09-5.33) | 5.22 |
| High SDI | 26.45  (21.54-31.14) | 38.2 | 11.31  (9.01-13.69) | 27.67 | 21.53  (16.61-26.40) | 31.22 | 11.84  (8.91-14.79) | **28.3** | 12.63  (-2.79-25.57) | 18.32 | 6.35  (-1.37-13.03) | 15.25 | 1.19  (0.62-1.92) | 1.73 | 0.98  (0.50-1.70) | 2.38 |
| High-middle SDI | 84.36  (65.31-106.93) | 37.22 | 34.61  (24.90-46.91) | **35.52** | 58.34  (41.14-79.02) | 26.18 | 26.73  (18.52-37.77) | 26.94 | 41.53  (-8.87-85.15) | 18.44 | 3.97  (-0.83-10.04) | 4.01 | 3.46  (1.29-7.66) | 1.55 | 1.90  (0.73-3.99) | 1.92 |
| Middle SDI | 75.93  (57.49-99.65) | 35.87 | 21.44  (15.64-28.35) | 28.29 | 46.26  (31.28-64.95) | 22.34 | 18.21  (12.92-24.91) | 23.77 | 43.27  (-9.61-89.00) | 20.68 | 8.05  (-1.69-17.61) | 10.52 | 5.67  (2.88-9.94) | 2.7 | 3.28  (1.74-5.17) | 4.29 |
| Low-middle SDI | 12.54  (9.71-15.49) | 23.75 | 8.14  (6.17-10.25) | 16.59 | 5.92  (3.47-8.36) | 11.57 | 7.76  (5.13-10.64) | 16.07 | 11.62  (-2.64-23.48) | 22.5 | 10.68  (-2.34-21.27) | 22.04 | 8.93  (5.41-13.25) | 17.15 | 6.82  (4.04-10.22) | **14.04** |
| Low SDI | 9.71  (7.19-12.62) | 13.02 | 6.75  (4.94-8.71) | 10.22 | 8.27  (3.34-12.09) | 11.4 | 8.78  (5.01-12.54) | 13.73 | 18.48  (-4.43-36.23) | 25.24 | 15.95  (-3.41-31.56) | **24.7** | 6.45  (3.90-9.75) | 8.8 | 4.64  (2.79-6.83) | 7.13 |
| **Women** | | | | | | | | | | | | | | | | |
| Global | 1.70  (1.28-2.15) | 4.03 | 0.59  (0.44-0.76) | 2.78 | 1.50  (0.93-2.10) | 3.67 | 0.81  (0.52-1.16) | 3.75 | 8.44  (-1.73-17.14) | 20.62 | 3.95  (-0.84-8.04) | 18.35 | 1.85  (0.97-2.96) | 4.43 | 1.26  (0.67-2.11) | 5.9 |
| High SDI | 2.38  (1.90-2.86) | 21.23 | 1.11  (0.86-1.38) | **14.2** | 1.98  (1.39-2.63) | 17.63 | 1.35  (0.92-1.81) | **16.68** | 1.99  (-0.43-4.01) | 17.77 | 1.32  (-0.28-2.72) | 16.36 | 0.04  (0.02-0.07) | 0.37 | 0.04  (0.02-0.07) | 0.5 |
| High-middle SDI | 1.71  (1.20-2.34) | 4.34 | 0.64  (0.47-0.86) | 5.68 | 1.86  (1.16-2.72) | 4.88 | 0.67  (0.44-0.97) | 5.69 | 6.83(-1.35-14.86) | 17.66 | 0.73  (-0.15-1.64) | 6.21 | 0.24  (0.09-0.52) | 0.61 | 0.10  (0.04-0.20) | 0.87 |
| Middle SDI | 1.62  (1.07-2.24) | 2.89 | 0.40  (0.28-0.55) | 2.17 | 1.37  (0.69-2.16) | 2.55 | 0.55  (0.34-0.80) | 2.89 | 11.12  (-2.30-23.56) | 20.46 | 2.81  (-0.63-5.79) | 14.88 | 1.13  (0.51-1.91) | 2.03 | 0.78  (0.35-1.43) | 4.22 |
| Low-middle SDI | 1.17  (0.77-1.71) | 2.36 | 0.47  (0.30-0.72) | 1.41 | 0.59  (0.24-0.97) | 1.26 | 0.63  (0.34-1.01) | 1.94 | 10.61  (-2.59-21.26) | 22.48 | 7.11  (-1.52-15.08) | 21.96 | 6.39  (3.41-10.59) | 13.08 | 3.55  (1.85-6.10) | **10.8** |
| Low SDI | 1.36  (0.88-1.97) | 2.03 | 0.66  (0.40-0.95) | 1.44 | 1.92  (0.47-3.25) | 3.1 | 1.54  (0.70-2.68) | 3.59 | 15.35  (-3.35-30.65) | 24.31 | 10.41  (-2.12-20.85) | **23.97** | 3.31  (1.68-5.65) | 5.05 | 1.85  (0.92-3.18) | 4.12 |

UI=uncertainty interval. DALY=disability-adjusted life year. ASDR=age-standardized DALYs rate.

**Supplementary Table 10. Global Age-Specific DALYs Rates and Proportional DALY Burden of Early-Onset Esophageal Cancer by Age Group and Sex in 2021**

| **Attributable risk factor** | **20 to 24** | | **25 to 29** | | **30 to 34** | | **35 to 39** | | **40 to 44** | | **45 to 49** | |
| --- | --- | --- | --- | --- | --- | --- | --- | --- | --- | --- | --- | --- |
|  | **Age-specific**  **DALY rate**  **(95% UI)** | **Proportion**  **%** | **Age-specific**  **DALY rate**  **(95% UI)** | **Proportion**  **%** | **Age-specific**  **DALY rate**  **(95% UI)** | **Proportion**  **%** | **Age-specific**  **DALY rate**  **(95% UI)** | **Proportion**  **%** | **Age-specific**  **DALY rate**  **(95% UI)** | **Proportion**  **%** | **Age-specific**  **DALY rate**  **(95% UI)** | **Proportion**  **%** |
| **Smoking** | | | | | | | | | | | | |
| Both | NA | NA | NA | NA | 1.78(1.37-2.26) | 11.28 | 5.35(4.13-6.86) | 15.94 | 16.85(12.84-21.36) | 20.76 | 42.80(32.40-54.57) | 25.56 |
| Men | NA | NA | NA | NA | 3.39(2.61-4.31) | 15.44 | 10.26(7.91-13.21) | 20.92 | 32.42(24.74-41.18) | 25.98 | 82.56(62.46-105.72) | 31.36 |
| Women | NA | NA | NA | NA | 0.13(0.10-0.17) | 1.4 | 0.35(0.27-0.44) | 2.00 | 1.03(0.77-1.32) | 2.81 | 2.67(1.97-3.45) | 3.79 |
| **Alcohol use** | | | | | | | | | | | | |
| Both | 0.58(0.41-0.76) | 12.45 | 1.01(0.72-1.32) | 13.57 | 2.67(1.92-3.42) | 16.93 | 5.96(4.26-7.77) | 17.75 | 15.03(10.87-19.60) | 18.52 | 32.07(23.69-42.65) | 19.17 |
| Men | 1.02(0.73-1.33) | 19.58 | 1.78(1.29-2.32) | 21.84 | 4.96(3.56-6.36) | 22.63 | 11.18(7.97-14.58) | 22.83 | 28.44(20.63-36.95) | 22.81 | 61.08(45.38-81.82) | 23.22 |
| Women | 0.13(0.07-0.21) | 3.19 | 0.22(0.12-0.34) | 3.24 | 0.32(0.19-0.48) | 3.42 | 0.63(0.40-0.93) | 3.55 | 1.40(0.92-2.00) | 3.8 | 2.79(1.82-3.87) | 3.96 |
| **Diet low in vegetables** | | | | | | | | | | | | |
| Both | NA | NA | 1.35(-0.27-2.68) | 18.08 | 2.35(-0.50-4.72) | 14.91 | 4.95(-1.01-10.06) | 14.73 | 11.47(-2.33-23.51) | 14.13 | 22.13(-4.89-44.93) | 13.24 |
| Men | NA | NA | 1.27(-0.26-2.50) | 15.55 | 2.83(-0.62-5.72) | 12.92 | 6.41(-1.30-13.40) | 13.07 | 15.80(-3.18-32.93) | 12.68 | 31.24(-7.04-65.08) | 11.9 |
| Women | NA | NA | 1.43(-0.29-2.88) | 21.26 | 1.86(-0.38-3.77) | 19.66 | 3.47(-0.72-6.95) | 19.37 | 7.06(-1.46-14.49) | 19.13 | 12.94(-2.82-26.40) | 18.32 |
| **Chewing tobacco** | | | | | | | | | | | | |
| Both | NA | NA | NA | NA | 0.89(0.58-1.22) | 5.67 | 1.96(1.28-2.76) | 5.84 | 4.87(3.10-6.88) | 6.01 | 9.03(6.03-12.51) | 5.41 |
| Men | NA | NA | NA | NA | 1.33(0.79-1.89) | 6.09 | 2.95(1.69-4.45) | 6.02 | 7.28(4.24-11.24) | 5.85 | 13.08(7.75-19.29) | 4.99 |
| Women | NA | NA | NA | NA | 0.44(0.20-0.82) | 4.65 | 0.96(0.49-1.71) | 5.34 | 2.42(1.29-4.02) | 6.56 | 4.95(2.70-8.09) | 7.01 |

UI=uncertainty interval. DALYs=disability-adjusted life year.

**Supplementary Table 11. Forecast of Global and Regional ASIR, ASMR (per 100,000), and Total Cases for Both Sexes, 2022-2050**

| **Categories** | **Incidence** | | | | | | **Mortality** | | | | | |
| --- | --- | --- | --- | --- | --- | --- | --- | --- | --- | --- | --- | --- |
|  | **Age-standardized rate (per 100000)** | | | **Cases** | | | **Age-standardized rate (per 100000)** | | | **Cases** | | |
|  | **2030** | **2040** | **2050** | **2030** | **2040** | **2050** | **2030** | **2040** | **2050** | **2030** | **2040** | **2050** |
| Global | 1.30  (0.88-1.72) | 1.50  (0.14-2.90) | 2.00  (0.00-6.88) | 51101.12  (34755.54-67446.70) | 61623.28  (5995.23-118820.50) | 84909.94  (0.00-286787.15) | 0.97  (0.64-1.30) | 1.11  (0.05-2.21) | 1.50  (0.00-5.50) | 38162.49  (25331.48-50993.51) | 45552.74  (2109.66-90480.29) | 63692.63  (0.00-228833.27) |
| Central Asia | 0.74  (0.31-1.19) | 0.68  (0.00-1.64) | 0.79  (0.00-3.91) | 370.72  (159.07-585.92) | 340.98  (0.00-822.78) | 385.24  (0.00-1904.95) | 0.67  (0.27-1.08) | 0.61  (0.00-1.49) | 0.71  (0.00-3.57) | 333.30  (138.90-531.45) | 307.16  (0.00-746.63) | 347.42  (0.00-1736.57) |
| Central Europe | 0.54  (0.26-0.84) | 0.56  (0.00-1.24) | 0.70  (0.00-2.73) | 306.67  (155.53-464.37) | 252.35  (0.00-546.98) | 274.74  (0.00-1045.56) | 0.47  (0.22-0.74) | 0.49(0.00-1.09) | 0.61  (0.00-2.42) | 264.61  (129.90-406.11) | 218.17  (0.00-479.56) | 239.52  (0.00-924.38) |
| Eastern Europe | 0.80  (0.24-1.36) | 0.84  (0.00-3.01) | 1.78  (0.00-25.40) | 903.04  (287.53-1525.11) | 667.02  (0.00-2392.31) | 1325.82  (0.00-18841.42) | 0.61  (0.22-1.01) | 0.57  (0.00-1.81) | 0.91  (0.00-9.44) | 688.90  (254.01-1130.46) | 451.90  (0.00-1436.33) | 673.18  (0.00-6983.46) |
| Australasia | 0.61  (0.18-1.09) | 0.60  (0.00-1.49) | 0.71  (0.00-3.36) | 88.69  (27.21-157.85) | 94.10  (0.00-231.28) | 115.99  (0.00-539.78) | 0.41  (0.10-0.80) | 0.41  (0.00-1.12) | 0.51  (0.00-2.95) | 60.81  (14.80-115.37) | 64.61  (0.00-174.36) | 83.97  (0.00-474.27) |
| High-income Asia Pacific | 0.55  (0.28-0.83) | 0.57  (0.00-1.22) | 0.72  (0.00-2.61) | 437.71  (230.55-650.10) | 404.36  (1.82-848.51) | 435.82  (0.00-1551.40) | 0.21  (0.09-0.36) | 0.21  (0.00-0.48) | 0.25  (0.00-1.02) | 171.43  (72.75-276.85) | 147.23  (0.00-334.70) | 149.22  (0.00-602.06) |
| High-income North America | 0.87  (0.52-1.22) | 0.98(0.06-1.95) | 1.23  (0.00-4.13) | 1485.66  (899.19-2073.23) | 1756.15  (116.08-3486.88) | 2227.42  (0.00-7398.63) | 0.57  (0.32-0.81) | 0.65  (0.03-1.33) | 0.86  (0.00-3.00) | 965.68  (557.24-1376.20) | 1167.06  (56.22-2364.64) | 1542.04  (0.00-5336.84) |
| Southern Latin America | 0.43  (0.14-0.74) | 0.37(0.00-0.91) | 0.41  (0.00-1.86) | 144.11  (48.09-246.17) | 130.75  (0.00-316.06) | 136.99  (0.00-618.34) | 0.35  (0.10-0.62) | 0.30  (0.00-0.76) | 0.33  (0.00-1.63) | 116.44  (34.62-205.20) | 104.87  (0.00-262.30) | 111.35  (0.00-538.66) |
| Western Europe | 0.74  (0.45-1.04) | 0.73  (0.03-1.45) | 0.80  (0.00-2.71) | 1448.38  (879.16-2019.07) | 1354.07  (67.17-2682.34) | 1473.07  (0.00-4917.31) | 0.45  (0.27-0.64) | 0.43  (0.04-0.86) | 0.47  (0.00-1.50) | 883.16  (529.27-1240.01) | 811.88  (77.86-1586.24) | 860.22  (0.00-2720.72) |
| Andean Latin America | 0.23  (0.03-0.45) | 0.27  (0.00-0.73) | 0.40  (0.00-2.30) | 71.98  (10.88-140.99) | 92.22  (0.00-253.72) | 145.48  (0.00-836.51) | 0.18  (0.02-0.37) | 0.19  (0.00-0.54) | 0.27  (0.00-1.56) | 56.08  (6.70-114.50) | 66.55  (0.00-187.99) | 97.09  (0.00-567.39) |
| Caribbean | 0.84  (0.32-1.41) | 0.95  (0.00-2.28) | 1.35  (0.00-6.39) | 190.57  (72.80-315.17) | 221.38  (0.00-525.71) | 294.00  (0.00-1368.62) | 0.76  (0.28-1.28) | 0.88  (0.00-2.10) | 1.28  (0.00-5.93) | 171.11  (63.45-285.89) | 203.79  (0.00-483.45) | 276.81  (0.00-1266.55) |
| Central Latin America | 0.32  (0.16-0.48) | 0.34  (0.02-0.69) | 0.40  (0.00-1.34) | 422.59  (214.44-632.63) | 487.15  (26.04-990.76) | 589.07  (0.00-1945.19) | 0.27  (0.13-0.42) | 0.28  (0.01-0.59) | 0.33  (0.00-1.14) | 359.68  (175.67-546.02) | 409.33  (15.41-843.38) | 489.54  (0.00-1648.27) |
| Tropical Latin America | 1.20  (0.75-1.64) | 1.44  (0.18-2.75) | 2.05  (0.00-6.19) | 1444.30  (919.50-1970.62) | 1697.62  (221.94-3208.74) | 2250.51  (0.00-6734.49) | 1.04  (0.64-1.43) | 1.23  (0.14-2.35) | 1.70  (0.00-5.20) | 1251.90  (784.63-1720.97) | 1442.45  (171.58-2748.56) | 1869.53  (0.00-5663.61) |
| North Africa and Middle East | 0.38  (0.24-0.53) | 0.39  (0.06-0.72) | 0.44  (0.00-1.27) | 1309.98  (826.27-1793.70) | 1358.71  (225.52-2525.55) | 1694.66  (0.00-4858.60) | 0.32  (0.20-0.45) | 0.32  (0.05-0.61) | 0.37  (0.00-1.09) | 1103.03  (679.41-1526.66) | 1137.94  (172.85-2139.76) | 1425.51  (0.00-4175.12) |
| South Asia | 0.84  (0.59-1.09) | 0.77  (0.16-1.37) | 0.77  (0.00-2.10) | 7956.86  (5609.52-10304.20) | 8302.73  (1746.12-14871.24) | 8789.13  (0.00-23895.74) | 0.74  (0.52-0.97) | 0.67  (0.14-1.21) | 0.67  (0.00-1.84) | 7047.74  (4948.98-9146.50) | 7286.55  (1513.44-13071.76) | 7694.94  (0.00-20976.92) |
| East Asia | 3.42  (1.71-5.13) | 5.76  (0.00-14.87) | 14.15  (0.00-88.19) | 27208.82  (13686.83-40730.80) | 37428.67  (0.00-96314.69) | 72662.58  (0.00-449580.16) | 2.19  (1.01-3.37) | 3.54  (0.00-9.80) | 8.96  (0.00-66.69) | 17436.32  (8103.86-26768.83) | 23082.28  (0.00-63613.69) | 46041.28  (0.00-339952.29) |
| Oceania | 0.38  (0.00-1.96) | 0.45  (0.00-3.53) | 0.83  (0.00-14.59) | 23.25  (0.00-130.92) | 33.24  (0.00-298.37) | 73.43  (0.00-1402.74) | 0.35  (0.00-1.86) | 0.43  (0.00-3.41) | 0.83  (0.00-14.46) | 21.68  (0.00-123.97) | 32.06  (0.00-288.72) | 73.40  (0.00-1392.35) |
| Southeast Asia | 0.57  (0.38-0.76) | 0.54  (0.11-0.97) | 0.58  (0.00-1.56) | 2005.65  (1340.57-2670.73) | 1947.32  (413.43-3496.70) | 2137.44  (0.00-5702.50) | 0.46  (0.30-0.63) | 0.43  (0.08-0.79) | 0.46  (0.00-1.25) | 1636.37  (1070.10-2202.65) | 1561.61  (299.49-2840.24) | 1682.20  (0.00-4570.90) |
| Central Sub-Saharan Africa | 1.60  (0.92-2.28) | 1.79  (0.07-3.56) | 2.35  (0.00-7.71) | 875.94  (493.38-1259.15) | 1347.76  (49.66-2700.20) | 2438.11  (0.00-8028.60) | 1.45  (0.82-2.08) | 1.58  (0.06-3.15) | 2.01  (0.00-6.62) | 792.98  (440.30-1146.27) | 1187.75  (39.78-2386.58) | 2087.39  (0.00-6888.79) |
| Eastern Sub-Saharan Africa | 2.11  (1.46-2.77) | 2.16  (0.47-3.84) | 2.45  (0.00-6.52) | 3786.15  (2592.81-4979.49) | 5454.24  (1174.05-9748.85) | 8409.83  (0.00-22389.14) | 1.93  (1.32-2.54) | 1.97  (0.41-3.54) | 2.26  (0.00-6.07) | 3458.76  (2348.46-4569.06) | 4992.67  (1026.72-8974.93) | 7756.30  (0.00-20854.62) |
| Southern Sub-Saharan Africa | 3.17  (0.55-6.51) | 10.49  (0.00-159.03) | 1644.77  (0.00-179483.26) | 1372.46  (238.95-2814.57) | 4849.67  (0.00-73201.08) | 804769.47  (0.00-87675936.27) | 2.80  (0.48-5.69) | 8.46  (0.00-115.61) | 982.70  (0.00-100321.60) | 1210.52  (210.24-2458.97) | 3914.87  (0.00-53261.55) | 480486.16  (0.00-48959637.81) |
| Western Sub-Saharan Africa | 0.84  (0.47-1.21) | 1.09  (0.00-2.33) | 1.78  (0.00-7.07) | 1619.89  (898.57-2341.33) | 2931.25  (8.86-6302.85) | 6697.41  (0.00-26727.77) | 0.77  (0.43-1.12) | 1.00  (0.00-2.16) | 1.63  (0.00-6.60) | 1489.29  (814.43-2164.26) | 2690.60  (9.70-5826.19) | 6148.55  (0.00-24962.92) |

ASMR=age-standardized mortality rate. ASIR=Age-standardized incidence rate.

**Supplementary Figure 1. Age-standardized incidence rates (A), mortality rates (B), and DALY rates (C) of early-onset esophageal cancer per 100,000 population in 2021, presented by countries and territories.** The global maps highlight variations in these rates across different regions, with detailed insets showing specific areas such as the Caribbean and Central America, Persian Gulf, Balkan Peninsula, Southeast Asia, West Africa, Eastern Europe, and Northern Europe. The color gradients represent the magnitude of the rates, with darker shades indicating higher values.

**Supplementary Figure 2. Global temporal trends of early-onset esophageal cancer (1990-2021).** The panels illustrate the incidence (top row), mortality (middle row), and DALYs (Disability-Adjusted Life Years) (bottom row) trends, reported as the total number of cases (left), crude rates per 100,000 population (middle), and age-standardized rates per 100,000 population (right). Data are stratified by sex, with both sexes combined, women, and men. Shaded areas represent 95% uncertainty intervals. The analysis reflects global patterns from 1990 to 2021.

**Supplementary Figure 3. Joinpoint regression analysis of the temporal trends in the burden of early-onset esophageal cancer from 1990 to 2021.** (A) Age-standardized incidence rates; (B) Age-standardized mortality rates; (C) Age-standardized DALY rates. Each joinpoint indicates a significant change in trend, with corresponding annual percentage changes (APCs) between the identified periods. The observed points and average annual percentage changes (AAPCs) are marked, reflecting the overall trend in each burden metric.


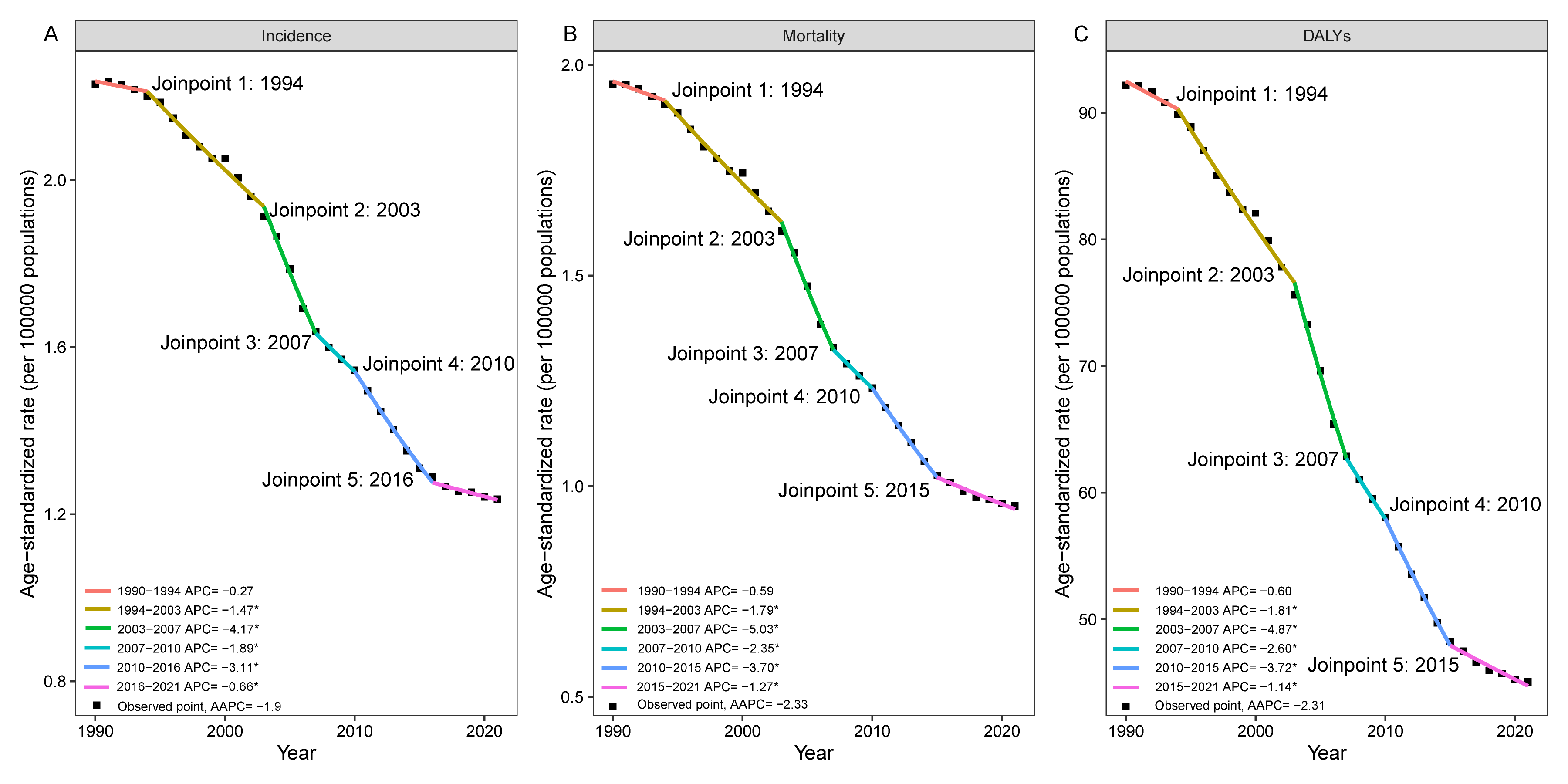


**Supplementary Figure 4. Hierarchical clustering of the average annual percentage changes (AAPC) in age-standardized incidence rates (ASIR) (A), age-standardized mortality rates (ASMR) (B), and age-standardized DALY rates (ASDR) (C) for early-onset esophageal cancer across the 21 Global Burden of Disease (GBD) regions from 1990 to 2021.**

**
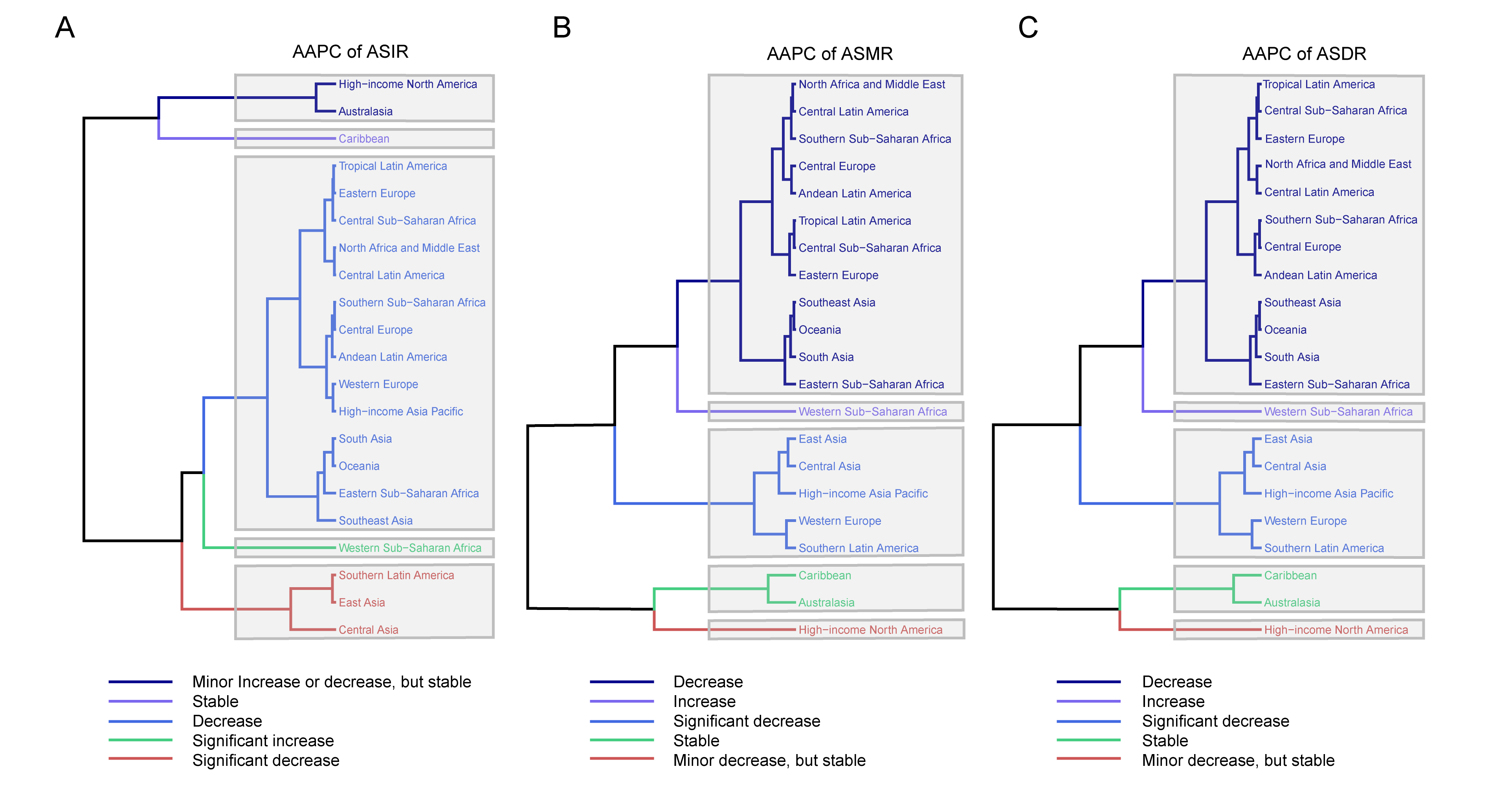
**

**Supplementary Figure 5.** **Joinpoint regression analysis of age-standardized incidence rates (A), mortality rates (B), and DALY rates (C) for early-onset esophageal cancer in the top three countries for each metric from 1990 to 2021.** (A) The countries with the highest incidence rates are Kazakhstan, Kyrgyzstan, and Armenia. Each joinpoint indicates a significant change in trend, with colored segments representing the annual percentage change (APC) for each period. (B) The countries with the highest mortality rates are the Republic of Korea, Kazakhstan, and Kyrgyzstan. The analysis displays joinpoints and corresponding trend changes for each country. (C) The countries with the highest DALY rates are the Republic of Korea, Kazakhstan, and Kyrgyzstan. The joinpoint analysis illustrates significant shifts in trend over the years for each country.

**Supplementary Figure 6. The average annual percentage change (AAPC) in age-standardized incidence rates (A), mortality rates (B), and DALY rates (C) for early-onset esophageal cancer from 1990 to 2021.** The global maps illustrate geographical variations in AAPC for each metric, with detailed insets for specific regions such as the Caribbean and Central America, Persian Gulf, Balkan Peninsula, Southeast Asia, West Africa, Eastern Europe, and Northern Europe. Different shades indicate the magnitude of change, with darker colors representing more significant increases or decreases in the respective rates.

**Supplementary Figure 7. Age-standardized incidence rates and mortality rates for early-onset esophageal cancer across 21 GBD regions (A, B) and 204 countries and territories (C, D) by SDI (Socio-Demographic Index) from 1990 to 2021.**

**Supplementary Figure 8. Frontier analysis based on the SDI and age-standardized mortality rate (ASMR) and Disability-Adjusted Life Years (DALYs) rate for early-onset esophageal cancer in 2021.** The frontier is represented by a solid black line, with countries and territories depicted as dots. In panels A and C, the color gradient illustrates the progression of years, from lighter shades representing 1990 to darker shades indicating 2021. Panels B and D highlight the top 15 countries with the largest deviation from the frontier in terms of early-onset esophageal cancer DALYs, marked in black. Countries with low SDI (<0.466) and minimal deviation from the frontier are labeled in blue, while those with high SDI (>0.81) and a relatively high deviation for their level of development are labeled in red. Red dots indicate an increase in age-standardized ASMR and DALYs rates from 1990 to 2021, whereas blue dots indicate a decrease. SDI=Socio-demographic Index.

**Supplementary Figure 9. Global trends in Disability-Adjusted Life Years (DALYs) attributed to Level 2 risk factors for early-onset esophageal cancer from 1990 to 2021.** The left panel shows the total DALYs counts (in millions) for tobacco use (blue), dietary risks (red), and alcohol use (green). The right panel presents the age-standardized DALYs rates (per 100,000 population) for the same risk factors over time. Shaded areas represent 95% uncertainty intervals, and the solid lines reflect the temporal trend for each risk factor. Tobacco use remains the largest contributor to DALYs throughout the study period, followed by dietary risks and alcohol use.


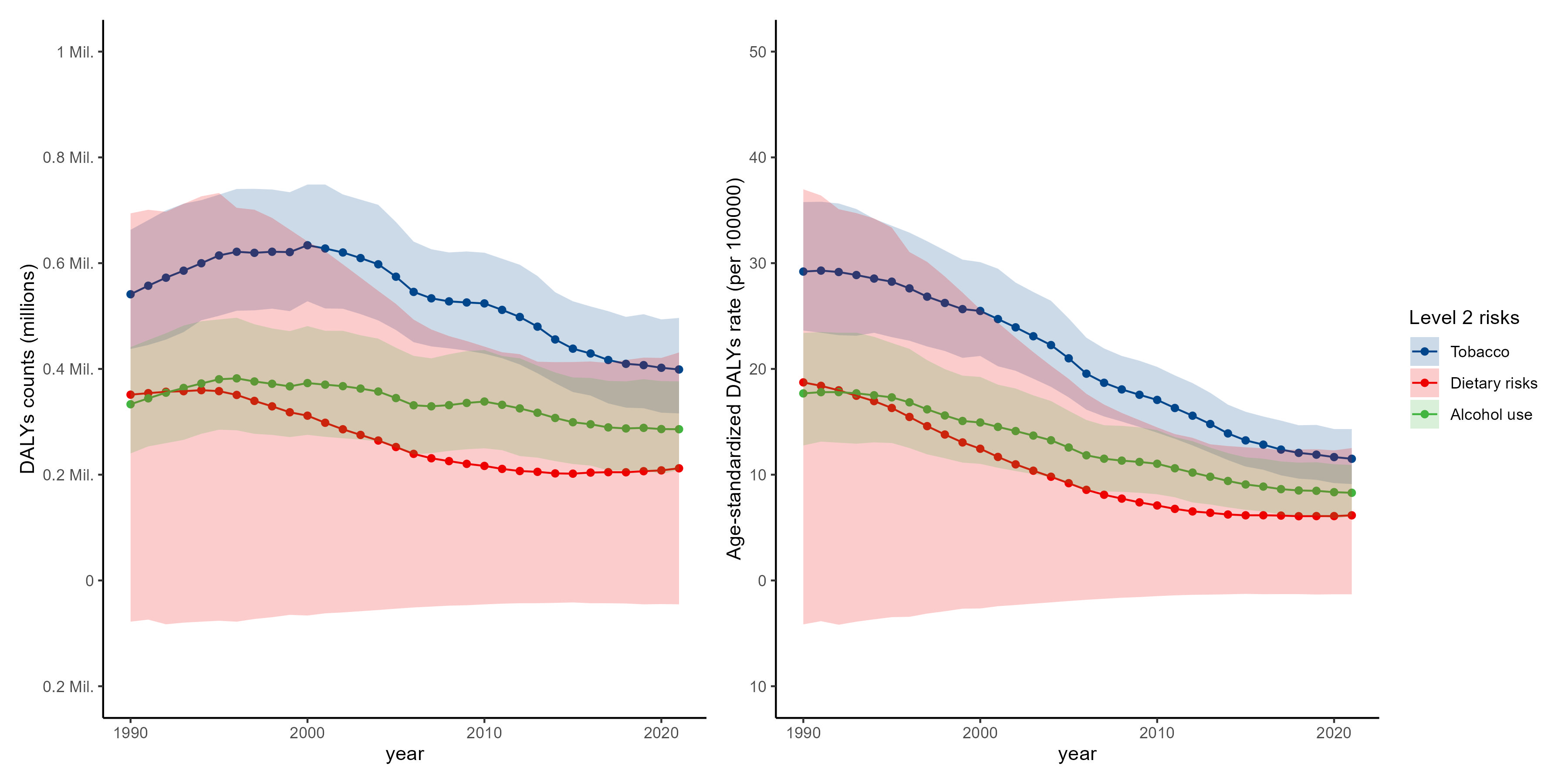


**Supplementary Figure 10.** **Global maps illustrating risk-attributable Disability-Adjusted Life Year (DALY) rates (per 100,000 population) for Level 2 risk factors contributing to early-onset esophageal cancer in 2021.** (A) Risk-attributable DALY rates due to tobacco use. (B) Risk-attributable DALY rates due to alcohol use.

(C) Risk-attributable DALY rates due to dietary risks. The color gradient represents the magnitude of DALY rates, with darker shades indicating higher risk-attributable DALYs. Insets provide a more detailed view of specific regions, including the Caribbean and Central America, Persian Gulf, Balkan Peninsula, Southeast Asia, West Africa, and Eastern Europe, highlighting regional differences in the burden of these risk factors.

**Supplementary Figure 11. Changes in risk factors contributing to early-onset esophageal cancer DALYs across age groups globally from 1990 to 2021.** (A) Proportional contribution of four risk factors (smoking, alcohol use, diet low in vegetables, and chewing tobacco) to early-onset esophageal cancer DALYs in 1990 and 2021, stratified by age and gender. Each stacked area illustrates the proportion of DALYs attributable to each risk factor for different age groups, ranging from 20-24 to 45-49 years, for both women and men. (B) Temporal trends in the proportion of DALYs linked to each risk factor from 1990 to 2021, depicted separately for each age group. The line graphs reveal how the contribution of these risk factors to the disease burden has evolved over the three decades. DALYs = disability-adjusted life years.

**Supplementary Figure 12.** **Global trends in the proportion of Disability-Adjusted Life Years (DALYs) attributable to four risk factors for early-onset esophageal cancer, stratified by sex (men, women, and both) from 1990 to 2021.** The color bars represent trends for both sexes combined (blue), women (red), and men (green). Over time, variations in the burden of each risk factor can be observed across different genders, with smoking and alcohol use remaining significant contributors to DALYs, especially among men.

**Supplementary Figure 13. Trends in the proportion of Disability-Adjusted Life Years (DALYs) attributable to four risk factors for early-onset esophageal cancer, stratified by sex and Socio-Demographic Index (SDI) from 1990 to 2021.** The colored bars represent trends for women (yellow) and men (blue) across different SDI regions. The graphs demonstrate how the burden of these risk factors varies by sex and region, with smoking and alcohol use contributing significantly in higher SDI regions, while dietary risks and chewing tobacco show more regional variation.

**Supplementary Figure 14.** **Projected number of cases and age-standardized rates for early-onset esophageal cancer globally from 2022 to 2050, by sex, using the Bayesian Age-Period-Cohort (BAPC) model.** (A) Projected number of cases and age-standardized incidence rates (ASIR) for both sexes combined, men, and women. The solid line represents observed data, while the dashed line represents predicted values. (B) Projected number of deaths and age-standardized mortality rates (ASMR) for both sexes combined, men, and women. The solid line represents observed data, while the dashed line represents predicted values. The bars illustrate the number of cases (left axis), while the lines represent the age-standardized rates (right axis). The shaded areas indicate the uncertainty intervals for the projections.
